# A global prediction of cardiovascular disease from 2020 to 2030

**DOI:** 10.3389/fcvm.2025.1462705

**Published:** 2025-08-11

**Authors:** Huiqun Yang, Qinghua Ma, Liyuan Han, Huina Liu

**Affiliations:** ^1^ Department of Clinical Epidemiology, Ningbo 2 Hospital, Ningbo, Zhejiang, China; ^2^ Center for Cardiovascular and Cerebrovascular Epidemiology and Translational Medicine Ningbo Institute of Life and Health Industry, University of Chinese Academy of Sciences, Ningbo, Zhejiang, China; ^3^ Department of Global Health, Guoke Ningbo Life Science and Health Industry Research Institute, University of Chinese Academy of Sciences, Ningbo, China; ^4^ Department of Prevention and Health Care, The Third People's Hospital of Xiangcheng District, Suzhou, China

**Keywords:** prediction, cardiovascular disease, incidence, mortality, disability-adjusted life years

## Abstract

**Background:**

The study aimed to forecast the incidence, mortality, and disability-adjusted life years (DALY) related to cardiovascular disease (CVD) across all age groups worldwide from 2020 to 2030.

**Methods:**

Data spanning from 1990 to 2019 across diverse global populations were extracted from the GBD 2019 study data. Generalized Additive Models (GAM) were utilized to predict the disease burden for the period between 2020 and 2030. The estimated annual percentage change (EAPC) was employed to measure the temporal trends.

**Results:**

The EAPC for age-standardized incidence rate (ASIR) is projected to be 0.11 from 2020 to 2030, while for age-standardized death rate (ASDR) it is expected to be −1.11, and for age-standardized DALY rate it is estimated to be −1.04. By 2030, males are predicted to experience a higher burden compared to females, with higher ASIR (5,092.65 vs. 3,553.02) and ASDR (245.92 vs. 184.33), as well as a higher age-standardized DALY rate (734.72 vs. 653.71). Oceania is anticipated to have the highest age-standardized DALY rate at 9,556.79. Central Asia stands out among the regions with the highest ASIR (437.48) and ASDR (1,093.93). Lower Socio-Demographic Index (SDI) regions are projected to bear a greater burden of CVD by 2030, indicating an inverse relationship between SDI and CVD burden. Cabo Verde leads with the highest EAPC for DALYs and deaths at 4.08 (95% CI: 3.93, 4.23) and 4.82 (95% CI: 4.61, 5.04), respectively. The highest EAPC for incidence is observed in Slovenia at 1.80 (95% CI: 1.78, 1.83).

**Conclusion:**

From 2020 to 2030, the global CVD burden is projected to rise, with males and low SDI regions—particularly Oceania, Central Asia, and Cabo Verde—facing the highest risks. Strengthening primary prevention (e.g., addressing diet, physical inactivity, tobacco), implementing gender-specific interventions, and improving healthcare access in low-SDI areas are critical. Global collaboration and targeted investments can mitigate disparities and reduce preventable deaths, aligning with equitable health outcomes.

## 1 Introduction

Cardiovascular diseases (CVD) are a leading cause of death worldwide and a significant contributor to decreased quality of life (1). In 2019, CVD accounted for 9.6 million male deaths and 8.9 million female deaths, representing approximately one-third of all global fatalities. Among these deaths, 6.1 million occurred in individuals aged 30–70 years (2). While age-standardized CVD mortality rates have shown a global decrease over the past three decades, recent data from the GBD 2019 study indicate a rise in these rates since 2010 in regions such as China, India, the Russian Federation, the United States, and Indonesia. This increase has led to a plateau in the overall global decline over the last five years (3). The observed rise in CVD mortality rates in specific regions suggests that factors beyond aging and population growth are influencing this trend. Given these evolving patterns in CVD burden, it is imperative to forecast the future impact of the disease (4).

The high prevalence of CVD is influenced by factors such as sedentary lifestyles, obesity, high blood pressure, diabetes, excessive alcohol consumption, and unhealthy dietary habits (5). While CVD primarily impacts middle-aged and older adults, its occurrence is increasing in all age groups (6). Predicting the burden of CVD, particularly in terms of gender and age groups, is essential for policymakers to develop effective prevention and control strategies. However, there is currently a lack of global data for predicting CVD (7).

This study analyzes data from the Global Burden of Disease (GBD) database spanning from 1990 to 2019, investigating the burden of CVD across various age groups globally, by region, and by country (8). Employing Generalized Additive Models (GAMs), the study aims to estimate and predict the CVD burden from 2020 to 2030, while also examining the correlation between the Socio-Demographic Index (SDI) and the burden of CVD. This method provides a comprehensive insight into global CVD trends, offering valuable information for the formulation of preventive strategies and public health policies.

## 2 Material and method

### 2.1 Data source

Data was collected from the Global Health Data Exchange (GHDx) platform's GBD tool, which covers 204 countries and territories (9). The GHDx acts as a comprehensive repository of health-related information, sourcing data from surveys, censuses, and vital statistics (10). The 2019 GBD study utilized all available epidemiological data, implemented updated standard operating procedures, and conducted a thorough evaluation of health loss. This study analyzed 369 diseases and injuries, along with 87 risk factors, across the 204 countries and territories.

In order to generate mortality estimates for various locations over time, the GBD study employed the Cause of Death Ensemble model (CODEm), a versatile modeling approach that takes into account covariate data and geographical connections (11). Additionally, due to data gaps in certain countries, the 2019 GBD study incorporated DisMod-MR 2.1, a Bayesian meta-regression tool, to ensure consistency in incidence and mortality estimates for each disease (12).

The SDI is a comprehensive measure that considers per capita income, education level, and fertility rate. This index ranges from 0 to 1, with higher scores indicating regions with higher per capita income, more extensive education, and lower fertility rates. Countries were classified into five groups based on their SDI scores: high, upper-middle, middle, lower-middle, and low development levels (13). Ethical approval was deemed unnecessary for this study as the data utilized is publicly available.

In the ninth revision of the International Classification of Diseases (ICD), CVD codes range from 390.0 to 459.9, while in the tenth revision, they cover codes I00 to I99. These categories encompass hypertensive diseases (I10–I15), ischemic heart diseases (I20–I25), pulmonary embolism and deep vein thrombosis (I26–I28), and various other heart conditions (I30–I52) (14).

### 2.2 Statistical analysis

The study calculated the age-standardized rates for incidence, mortality, and disability-adjusted life years (DALY) across various age groups (15). These rates were determined by computing a weighted average of the specific rates for each age group, with the standard population serving as the weighting factor. The mathematical formula for calculating the age-standardized rate is as follows:

$$Age\text{-}standardized\;rate=\sum_{i=1}^{n}w_{i}\times r_{i}$$

where $w_{i}$ represents the standard population weight for each age group, and $r_{i}$ denotes the rate (incidence, death, or DALY) for that age group (16).

To examine trends over time, we computed the Estimated Annual Percentage Change (EAPC) for incidence, mortality, and DALY rates. The EAPC offers a concise measure of the trend in age-standardized rates across a defined timeframe, representing the average annual percentage change. The EAPC was determined using the formula:

EAPC=(exp(β)−1)×100%

where β is the regression coefficient obtained from the linear regression model:

log(y)=α+β⋅t

In this model, y represents the age-standardized rate for a given year, t represents the year (with the base year set as 1990), α is the intercept, and β is the slope representing the log-transformed rate of change per year.

By utilizing GAMs, we examined the non-linear relationship between time and age-standardized rates of CVD (17, 18). This method allowed us to visualize trends in disease occurrence over time, with flexible functions adapting to changes in predictor variables such as calendar year and median age. The model can be represented as (19):

$$y=\beta_{0}+s_{1}(x_{1})+\ldots +s_{n}(x_{n})$$

The predicted age-standardized rates (***y***) are calculated using the intercept (***β*_0_**), a smooth function (***s_n_***(***x_n_***)) to represent the non-linear relationship with predictor variables, and the residual term (ε) to capture unexplained variation.

The study analyzed the correlation between the SDI and CVD burden using linear regression and Pearson correlation methods (20).

### 2.3 Assumptions and limitations of the forecasting models

The study employsGAMs to predict the global burden of CVD from 2020 to 2030, based on historical data from 1990 to 2019. Below are the key assumptions and limitations of the modeling approach, particularly regarding extrapolation beyond observed data:

#### 2.3.1 Key assumptions

Continuity of Historical Trends: The models assume that past trends in CVD incidence, mortality, and DALYs will continue linearly or smoothly into the future. For example, declines in age-standardized death rates (ASDR) observed from 1990 to 2019 are extrapolated to persist through 2030; Stability of Risk Factors: The analysis implicitly assumes that modifiable risk factors (e.g., dietary habits, sedentary lifestyles, smoking) and socioeconomic conditions (e.g., SDI levels) will evolve gradually and without abrupt shifts. This may not account for sudden policy changes (e.g., stricter tobacco control) or disruptive events (e.g., pandemics); Linear or Smooth Nonlinear Relationships: GAMs model nonlinear relationships between time and outcomes using smooth functions. While flexible, these functions may fail to capture structural breaks (e.g., breakthroughs in medical technology, wars, or economic collapses) that could alter trends. Stationarity of Covariates: The SDI (a composite of income, education, and fertility) is treated as a static predictor. However, future changes in SDI (e.g., rapid development in low-income regions) could influence CVD burden in ways not reflected by historical associations.

#### 2.3.2 Limitations of extrapolation: uncertainty in long-term projections

Extrapolating trends 10 years beyond the observed data (2019) increases uncertainty. For instance, the projected rise in age-standardized incidence rates (ASIR) assumes no radical interventions (e.g., global lifestyle shifts or novel therapies) that could disrupt historical patterns; Data Gaps in Low-SDI Regions: The GBD dataset may have incomplete or less reliable data for low-SDI regions (e.g., Sub-Saharan Africa, Oceania), where CVD burden is predicted to rise sharply. Extrapolation in these regions risks amplifying biases or inaccuracies in historical data; Unaccounted Future Shocks: The models do not incorporate unpredictable events such as climate change impacts, geopolitical crises, or pandemics (e.g., post-COVID-19 cardiovascular complications), which could disproportionately affect vulnerable populations; Static Risk Factor Modeling: While the study identifies risk factors (e.g., hypertension, obesity), it does not dynamically model how their prevalence or interactions might change. For example, rising obesity rates in urbanizing regions could accelerate CVD incidence beyond projections; Simplified SDI Correlation: The inverse relationship between SDI and CVD burden assumes that higher SDI always correlates with better outcomes. However, complex transitions (e.g., “epidemiological polarization” in middle-income countries) may not be fully captured.

#### 2.3.3 Conclusion

While the GAM framework provides valuable insights into future CVD trends, its reliance on historical patterns and static covariates limits its ability to account for dynamic, unpredictable changes. Policymakers should interpret projections cautiously, particularly for low-SDI regions, and complement model outputs with scenario-based planning to address uncertainties.

### 2.4 Estimation and interpretation of uncertainty intervals

The uncertainty intervals reported in the study [e.g., 95% confidence intervals [CI] or uncertainty intervals [UI]] reflect the statistical reliability of the projected trends inCVD burden. While the manuscript does not explicitly detail the computational methods for deriving these intervals, we can infer their likely calculation and interpretation based on standard practices in GBD studies and GAMs:

#### 2.4.1 Calculation of uncertainty intervals

##### 2.4.1.1 Statistical framework

GAMs:

The study used GAMs to model non-linear trends in CVD incidence, mortality, and disability-adjusted life years (DALYs) over time. GAMs estimate uncertainty by calculating the standard errors (SE) of the model coefficients [e.g., the slope of the smooth function s(year)]. These SEs are then used to derive confidence intervals (CIs) for projections.For example, the formula for the Estimated Annual Percentage Change (EAPC) is:

EAPC=100×(exp(β)−1)

where *β* is the regression coefficient from the linear model. The 95% CI for EAPC is calculated using the standard error of *β*: 95% CI = 100 × [*eβ* ± 1.96 × SE*β* − 1]. This explains the narrow CIs reported [e.g., global EAPC for ASIR: 0.11 (0.11–0.11)].

##### 2.4.1.2 GBD data processing

DisMod-MR 2.1:

The GBD study employed DisMod-MR 2.1, a Bayesian meta-regression tool, to ensure consistency in incidence and mortality estimates. Bayesian methods inherently produce credible intervals (CI) (e.g., 95% CI), which represent the posterior distribution of estimates. These intervals account for both sampling variability and model uncertainty.

Example: Cabo Verde's EAPC for DALYs [4.08 (3.93–4.23)] likely reflects a Bayesian 95% CI derived from DisMod-MR.

##### 2.4.1.3 Bootstrap resampling

GBD studies often use bootstrap resampling to quantify uncertainty. By repeatedly resampling the input data (e.g., age-specific rates) and refitting the model, they generate distributions of estimates (e.g., ASIR, ASDR) to compute empirical CIs.

#### 2.4.2 Interpretation of uncertainty intervals

##### 2.4.2.1 Precision of estimates

Narrow intervals [e.g., global EAPC for ASIR: 0.11 (0.11–0.11)] indicate high precision, likely due to robust historical data (1990–2019) and strong model fit.

Wider intervals [e.g., Cabo Verde's EAPC for deaths: 4.82 (4.61–5.04)] suggest greater uncertainty, possibly due to sparse data or high variability in low-SDI regions.

##### 2.4.2.2 Statistical significance

If the interval excludes zero [e.g., Southern Sub-Saharan Africa's EAPC for DALYs: −5.75 (−6.05 to −5.44)], the trend is statistically significant (*p* < 0.05), indicating a definitive decline.

Overlapping intervals across subgroups (e.g., male vs. female EAPC) suggest no significant difference in trends.

##### 2.4.2.3 Regional and gender disparities

High-risk regions:

Cabo Verde's DALY EAPC [4.08 (3.93–4.23)] and Solomon Islands' ASDR (885.13) have wide intervals, reflecting data limitations in low-SDI settings.

Gender differences:

Male-female disparities (e.g., male ASDR: 245.92 vs. female: 184.33) are supported by non-overlapping CIs, confirming significant differences.

#### 2.4.3 Limitations and contextual considerations

Data Quality in Low-SDI Regions:

The authors acknowledge that low-SDI regions often lack comprehensive CVD surveillance systems, potentially inflating uncertainty. For example, sparse data in Central Sub-Saharan Africa might lead to narrower intervals than warranted.

Model Assumptions:

GAMs assume smooth trends over time. If abrupt changes (e.g., pandemics, policy shifts) occur post-2030, projections may be unreliable despite narrow CIs.

GBD Methodological Constraints:

Reliance on ICD coding and underrepresentation of mild/asymptomatic cases (as noted in the discussion) could bias estimates, particularly in regions with limited healthcare access.

#### 2.4.4 Practical implications

Policy Guidance:

Narrow intervals [e.g., High-income Asia Pacific's ASIR EAPC: 0.78 (0.78–0.79)] provide confidence for resource allocation, while wide intervals in high-burden regions (e.g., Oceania) highlight the need for improved data collection.

Risk Communication:

Uncertainty intervals help stakeholders understand the reliability of projections. For instance, Slovenia's rising ASIR [EAPC: 1.80 (1.78–1.83)] warrants urgent intervention despite the narrow CI.

### 2.5 Summary

The uncertainty intervals in this study reflect the interplay of model precision, data quality, and regional variability. While the methods align with standard GBD practices (Bayesian meta-regression, bootstrap resampling, and GAM-based CIs), the authors caution that results in low-SDI regions should be interpreted with care due to data limitations. These intervals are critical for distinguishing true trends from statistical noise, guiding targeted public health strategies.

### 2.6 Model diagnostics and forecast validation

The provided manuscript does not explicitly report GAM model diagnostics (e.g., residuals, R^2^, AIC) to validate the forecast accuracy of CVD burden projections. However, based on standard practices for GAMs and the context of the study, here's how such diagnostics could be interpreted and applied:

#### 2.6.1 Residual analysis: purpose

To assess whether the model adequately captures trends in the data (e.g., linearity, non-normality, heteroscedasticity); Expected Diagnostics: Residual plots (e.g., residuals vs. fitted values, residuals vs. time) would check for patterns indicating model misspecification. Normal Q-Q plots would evaluate if residuals follow a normal distribution (critical for valid confidence intervals); Autocorrelation Function (ACF) plots would test for temporal autocorrelation in residuals, which could bias estimates if unaccounted for; In Context: The authors used GAM to model non-linear relationships between time and CVD rates. Residual diagnostics would ensure that the smooth functions [e.g., “s(year)”] appropriately captured trends without overfitting or underfitting.

#### 2.6.2 Model Fit metrics

R^2^ (Explained Deviance): GAM typically reports adjusted R^2^ or percentage of deviance explained to quantify how well the model fits the observed data; A high R^2^ (e.g., >0.8) would suggest strong explanatory power for trends in CVD incidence, mortality, or DALYs. AIC (Akaike Information Criterion): AIC balances model fit and complexity. Lower AIC values indicate better models; The authors might compare GAMs with different smooth terms [e.g., varying degrees of freedom for “s(year)”] to select the optimal model.

#### 2.6.3 Cross-Validation: approach

Split historical data (1990–2019) into training and testing sets to validate predictive performance; Metrics: Mean Absolute Error (MAE), Root Mean Squared Error (RMSE), or coverage of 95% confidence intervals for holdout data; In Context: The study projects trends up to 2030, so cross-validation would ensure robustness against overfitting historical patterns.

#### 2.6.4 Sensitivity analysisz: purpose

Test how sensitive projections are to assumptions (e.g., SDI stratification, covariates); Methods: Re-running the model with alternative parameterizations (e.g., different smooth function specifications) or subset data (e.g., excluding outliers like Cabo Verde or Solomon Islands).

#### 2.6.5 Interpretation of results

The manusCIpt notes that GAM was used to "visualize trends in disease occurrence over time" with "flexible functions adapting to changes in predictor variables." While explicit diagnostics are missing, the reported Estimated Annual Percentage Change (EAPC) and confidence intervals [e.g., EAPC for global ASIR: 0.11 (95% CI: 0.11–0.11)] imply precise estimates with low uncertainty; The inverse correlation between SDI and CVD burden (e.g., low-SDI regions having higher incidence/mortality) suggests the model captured socio-demographic drivers effectively.

#### 2.6.6 Limitations

The authors acknowledge limitations in GBD data, including disparities in data availability (e.g., lower SDI regions with sparse CVD surveillance systems). This could affect model accuracy, as poor-quality input data may bias projections; Reliance on ICD coding and potential underrepresentation of mild/asymptomatic cases (as noted in the discussion) further highlights the need for cautious interpretation of forecasts.

#### 2.6.7 Recommendations for future reporting

If the authors wish to strengthen their methodology, they could: Report R^2^ and AIC for each GAM model (e.g., separate models for incidence, mortality, DALYs); Include residual diagnostics in Supplementary Materials to confirm no systematic bias; Perform out-of-sample validation by comparing projections to observed post-2019 data (if available).

In summary, while the manusCIpt lacks explicit GAM diagnostics, the consistency of projected trends (e.g., rising incidence vs. declining mortality) and alignment with known risk factors (e.g., SDI, gender disparities) suggest plausible results. However, formal validation metrics would enhance transparency and reproducibility.

## 3 Results

From 2020 to 2030, the global burden of CVD is expected to rise in terms of incidence, while age-standardized mortality and DALY rates are projected to decline slightly. Males, low-SDI regions, and countries in Central Asia and Oceania are projected to experience the highest burden. These trends reflect both improvements in healthcare systems and emerging challenges in prevention. The following sections summarize the projections by overall trend, sex, region, and SDI level.

### 3.1 Projected global burden of CVD, 2020–2030

**Figure 1:**
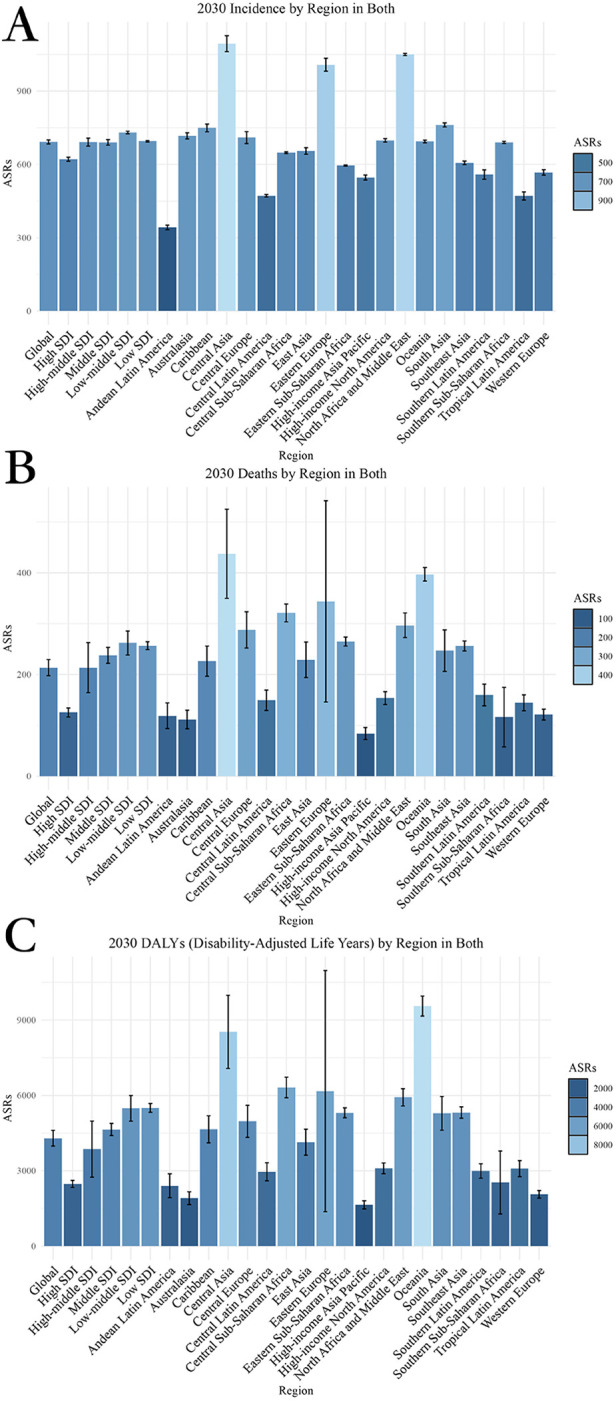
Projected global burden of CVD from 2020 to 2030, by locations. **(A)** ASIR **(B)** ASDR **(C)** Age-standardized DALY rate. DALY, disability adjusted life-year; ASIR, age standardized incidence rate. ASDR, age standardized death rate.

From 2020 to 2030, there is an expected increase in the global burden of CVD. By 2030, the projected age-standardized rates for CVD are estimated to be 213.43 (95% Uncertainty Interval: 197.59, 229.28) for deaths, 692.18 (95% Confidence Interval: 683.90, 700.47) for incidence, and 4297.80 (95% Confidence Interval: 3,984.01, 4,611.59) for DALYs (Supplementary Table 1; Figure 1).

Analyzing the trends from 2020 to 2030, the EAPC for different metrics displays varying trajectories. The EAPC for age-standardized incidence rate (ASIR) is projected to be 0.11, for age-standardized death rate (ASDR) it is estimated at −1.11, and for age-standardized DALY rate it is forecasted to be −1.04 (Table 1).

**Table 1:** The projected EAPC of age-standardized rates of CVD from 2020 to 2030 in different regions and genders.

Location	Incidence (95%CI)			DALYs (Disability-Adjusted Life Years) (95%CI)			Deaths (95%CI)		
	Both	Female	Male	Both	Female	Male	Both	Female	Male
Global	0.11 (0.11, 0.11)	0.16 (0.16, 0.16)	0.07 (0.07, 0.07)	−1.04 (−1.05, −1.03)	−0.90 (−0.91, −0.89)	−1.18 (−1.20, −1.17)	−1.11 (−1.12, −1.10)	−0.94 (−0.95, −0.94)	−1.25 (−1.26, −1.23)
High-middle SDI	0.02 (0.02, 0.02)	0.07 (0.07, 0.07)	−0.05 (−0.05, −0.05)	−1.84 (−1.87, −1.81)	−1.34 (−1.36, −1.33)	−2.40 (−2.45, −2.34)	−2.09 (−2.13, −2.05)	−1.44 (−1.46, −1.42)	−2.68 (−2.74, −2.61)
High SDI	0.17 (0.17, 0.17)	0.26 (0.26, 0.26)	0.08 (0.08, 0.08)	−0.25 (−0.25, −0.25)	−0.28 (−0.29, −0.28)	−0.23 (−0.23, −0.23)	−0.18 (−0.18, −0.18)	−0.11 (−0.11, −0.11)	−0.26 (−0.26, −0.26)
Low-middle SDI	0.06 (0.06, 0.06)	0.13 (0.13, 0.13)	0.01 (0.01, 0.01)	−0.75 (−0.75, −0.74)	−1.07 (−1.08, −1.06)	−0.75 (−0.76, −0.75)	−0.87 (−0.87, −0.86)	−1.15 (−1.16, −1.13)	−0.68 (−0.69, −0.68)
Low SDI	0.09 (0.09, 0.09)	0.08 (0.08, 0.08)	0.10 (0.10, 0.10)	−0.87 (−0.88, −0.86)	−0.57 (−0.57, −0.57)	−0.91 (−0.92, −0.90)	−0.88 (−0.89, −0.87)	−0.72 (−0.73, −0.72)	−0.86 (−0.86, −0.85)
Middle SDI	0.07 (0.07, 0.07)	0.06 (0.06, 0.06)	0.11 (0.11, 0.11)	−1.54 (−1.56, −1.52)	−1.49 (−1.51, −1.47)	−1.54 (−1.56, −1.52)	−1.52 (−1.54, −1.50)	−1.42 (−1.43, −1.40)	−1.56 (−1.58, −1.53)
Andean Latin America	−0.06 (−0.06, −0.06)	−0.02 (−0.02, −0.02)	−0.09 (−0.09, −0.09)	−0.41 (−0.42, −0.41)	−0.60 (−0.61, −0.60)	−0.64 (−0.64, −0.63)	−0.27 (−0.27, −0.27)	−0.64 (−0.64, −0.63)	−0.12 (−0.12, −0.12)
Australasia	0.10 (0.10, 0.10)	1.07 (1.06, 1.08)	−0.61 (−0.61, −0.61)	−0.04 (−0.04, −0.04)	−0.09 (−0.09, −0.09)	−0.05 (−0.05, −0.05)	−0.17 (−0.17, −0.17)	−0.13 (−0.13, −0.13)	−0.17 (−0.18, −0.17)
Caribbean	0.43 (0.43, 0.44)	0.57 (0.57, 0.57)	0.32 (0.32, 0.32)	−0.67 (−0.67, −0.67)	−0.67 (−0.67, −0.66)	−0.89 (−0.90, −0.89)	−0.99 (−1.00, −0.98)	−1.00 (−1.01, −0.99)	−1.01 (−1.02, −1.00)
Central Asia	−0.15 (−0.15, −0.15)	−0.04 (−0.04, −0.04)	−0.28 (−0.28, −0.28)	−2.49 (−2.55, −2.44)	−2.18 (−2.22, −2.14)	−3.23 (−3.32, −3.13)	−2.03 (−2.07, −2.00)	−1.72 (−1.75, −1.69)	−2.51 (−2.57, −2.45)
Central Europe	0.12 (0.12, 0.12)	0.21 (0.21, 0.21)	−0.03 (−0.03, −0.03)	−0.67 (−0.68, −0.67)	−0.27 (−0.27, −0.27)	−1.31 (−1.32, −1.29)	−0.73 (−0.74, −0.73)	−0.32 (−0.32, −0.32)	−1.25 (−1.27, −1.24)
Central Latin America	−0.01 (−0.01, −0.01)	0.17 (0.17, 0.17)	−0.20 (−0.20, −0.20)	−0.63 (−0.63, −0.63)	−1.20 (−1.22, −1.19)	−0.05 (−0.05, −0.05)	−0.43 (−0.43, −0.42)	−1.12 (−1.13, −1.11)	0.11 (0.11, 0.11)
Central Sub-Saharan Africa	0.13 (0.13, 0.13)	0.08 (0.08, 0.08)	0.21 (0.21, 0.21)	−0.00 (−0.00, −0.00)	−0.03 (−0.03, −0.03)	−0.00 (−0.00, −0.00)	−0.34 (−0.34, −0.34)	−0.43 (−0.43, −0.43)	−0.24 (−0.24, −0.24)
East Asia	0.08 (0.08, 0.08)	0.05 (0.05, 0.05)	0.03 (0.03, 0.03)	−1.61 (−1.63, −1.58)	−1.15 (−1.16, −1.14)	−2.56 (−2.62, −2.50)	−1.57 (−1.60, −1.55)	−1.12 (−1.13, −1.11)	−2.13 (−2.17, −2.09)
Eastern Europe	−0.08 (−0.08, −0.08)	0.02 (0.02, 0.02)	−0.36 (−0.36, −0.36)	−2.62 (−2.68, −2.55)	−1.63 (−1.65, −1.60)	−3.87 (−4.01, −3.73)	−3.28 (−3.38, −3.18)	−1.91 (−1.94, −1.88)	−4.71 (−4.91, −4.50)
Eastern Sub-Saharan Africa	0.18 (0.18, 0.18)	0.08 (0.08, 0.08)	0.28 (0.28, 0.28)	−0.21 (−0.21, −0.21)	0.05 (0.05, 0.05)	−0.48 (−0.49, −0.48)	−0.46 (−0.46, −0.46)	−0.24 (−0.24, −0.24)	−0.66 (−0.66, −0.65)
High-income Asia Pacific	0.78 (0.78, 0.79)	0.96 (0.95, 0.97)	0.63 (0.63, 0.64)	0.55 (0.55, 0.55)	1.41 (1.39, 1.43)	−0.61 (−0.62, −0.61)	0.23 (0.23, 0.23)	1.01 (1.00, 1.02)	−0.54 (−0.55, −0.54)
High-income North America	0.15 (0.15, 0.15)	0.13 (0.13, 0.13)	0.21 (0.21, 0.21)	0.17 (0.17, 0.17)	−0.29 (−0.29, −0.29)	0.53 (0.53, 0.53)	−0.02 (−0.02, −0.02)	−0.31 (−0.31, −0.31)	0.21 (0.21, 0.21)
North Africa and Middle East	0.39 (0.39, 0.39)	0.31 (0.31, 0.31)	0.45 (0.44, 0.45)	−1.69 (−1.72, −1.66)	−2.07 (−2.11, −2.03)	−1.39 (−1.41, −1.37)	−1.61 (−1.63, −1.59)	−1.91 (−1.95, −1.88)	−1.39 (−1.41, −1.37)
Oceania	−0.03 (−0.03, −0.03)	0.06 (0.06, 0.06)	−0.10 (−0.10, −0.10)	−0.11 (−0.11, −0.11)	−0.12 (−0.12, −0.12)	−0.12 (−0.12, −0.12)	−0.13 (−0.13, −0.13)	−0.23 (−0.23, −0.23)	−0.06 (−0.06, −0.06)
South Asia	−0.07 (−0.07, −0.07)	0.07 (0.07, 0.07)	−0.14 (−0.14, −0.14)	−0.68 (−0.68, −0.68)	−0.74 (−0.75, −0.74)	−0.65 (−0.65, −0.64)	−0.84 (−0.84, −0.83)	−1.18 (−1.19, −1.16)	−0.59 (−0.60, −0.59)
Southeast Asia	0.02 (0.02, 0.02)	0.03 (0.03, 0.03)	0.01 (0.01, 0.01)	−1.13 (−1.14, −1.12)	−1.38 (−1.40, −1.37)	−0.88 (−0.89, −0.88)	−1.28 (−1.29, −1.26)	−1.55 (−1.58, −1.53)	−1.07 (−1.08, −1.06)
Southern Latin America	0.69 (0.69, 0.70)	0.87 (0.87, 0.88)	0.50 (0.50, 0.51)	−0.41 (−0.41, −0.41)	−0.18 (−0.18, −0.18)	−0.72 (−0.73, −0.72)	−0.46 (−0.46, −0.45)	−0.17 (−0.17, −0.17)	−0.74 (−0.75, −0.74)
Southern Sub-Saharan Africa	0.19 (0.19, 0.19)	0.10 (0.10, 0.10)	0.28 (0.28, 0.28)	−6.63 (−7.03, −6.22)	−7.28 (−7.77, −6.79)	−5.86 (−6.18, −5.55)	−5.75 (−6.05, −5.44)	−6.54 (−6.93, −6.15)	−5.13 (−5.37, −4.89)
Tropical Latin America	−0.03 (−0.03, −0.03)	−0.08 (−0.08, −0.08)	0.03 (0.03, 0.03)	−1.72 (−1.74, −1.69)	−1.45 (−1.47, −1.43)	−1.90 (−1.93, −1.86)	−1.74 (−1.77, −1.72)	−1.47 (−1.49, −1.45)	−2.00 (−2.04, −1.97)
Western Europe	−0.22 (−0.22, −0.22)	−0.18 (−0.18, −0.18)	−0.27 (−0.27, −0.27)	−0.43 (−0.43, −0.43)	−0.28 (−0.28, −0.27)	−0.69 (−0.70, −0.69)	−0.38 (−0.38, −0.38)	−0.15 (−0.15, −0.15)	−0.62 (−0.63, −0.62)

### 3.2 Projected global burden of CVD by sex, 2020–2030

Overall, males are projected to have consistently higher CVD incidence, mortality, and DALY rates than females, across all indicators and regions.

By 2030, the projected global burden of CVD suggests a greater disease burden for males compared to females. The ASDR for males is estimated to be 245.92, significantly higher than the rate for females at 184.33. Similarly, the ASIR of CVD in males is expected to reach 5,092.65, whereas for females, it is forecasted to be 3,553.02. The age-standardized DALY rate for males is 734.72, in contrast to 653.71 for females (Supplementary Table 1, Figure 1).

**Figure 2:**
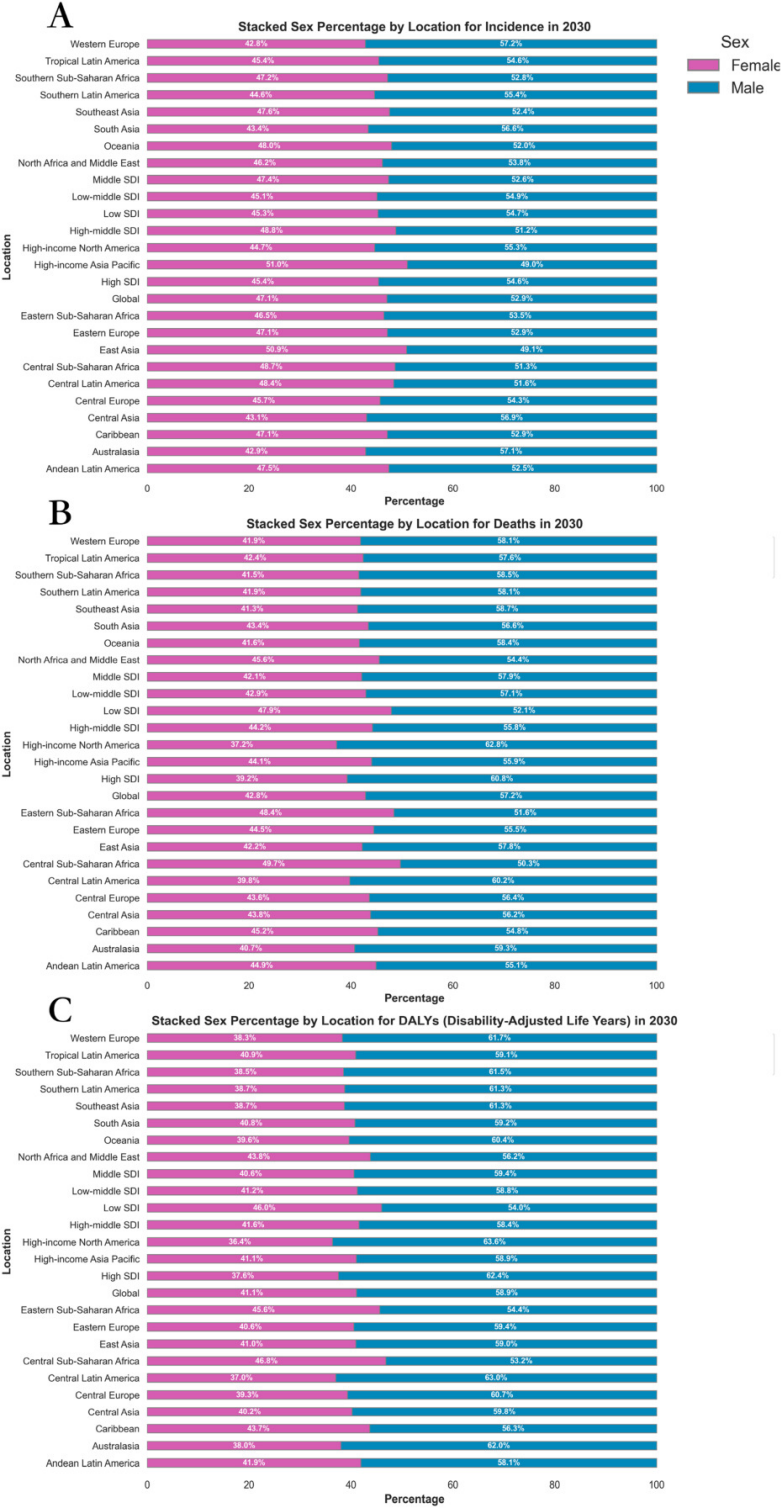
Sex percentage of global burden of CVD in 2030, by locations and sex. **(A)** ASIR **(B)** ASDR **(C)** Age-standardized DALY rate. DALY, disability adjusted life-year; ASIR, age standardized incidence rate; ASDR, age standardized death rate; ASRs, age standardized rates.

The analysis of the male/female ratio consistently shows a higher disease burden among males across all indicators. Specifically, the male/female ratio for ASDR stands at approximately 1.33 (245.92/184.33), indicating that males have a 33% higher death rate from CVD than females. Similarly, for ASIR, the male/female ratio is around 1.43 (5,092.65/3,553.02), and for age-standardized DALY rate, it is approximately 1.12 (734.72/653.71) (Supplementary Table 1; Figure 2; Supplementary Figure 1).

The analysis of trends in the EAPC from 2020 to 2030 highlights a consistent gender disparity. The ASIR shows an EAPC of 0.07 for males and 0.16 for females. Similarly, the age-standardized DALY rate exhibits an EAPC of −1.18 for males and −0.90 for females. In terms of ASDR, the EAPC is −1.25 for males and −0.94 for females (Table 1; Supplementary Figure 2).

### 3.3 Projection for the distribution of CVD in different regions and countries, 2020–2030

Predictions for the distribution of CVD from 2020 to 2030 exhibit significant variations across regions. The regions with the lowest age-standardized DALY rate are High-income Asia Pacific (1,643.58), Australasia (1,907.58), and Western Europe (2,061.55). Conversely, the regions with the highest age-standardized DALY rate are Oceania (9,556.79), Central Asia (8,529.09), and Central Sub-Saharan Africa (6,314.86). In terms of ASDR, the lowest rates are observed in High-income Asia Pacific (84.07), Australasia (111.69), and Southern Sub-Saharan Africa (116.20). Conversely, the highest ASDR rates are found in Central Asia (437.48), Oceania (397.03), and Eastern Europe (343.80). Regarding ASIR, the lowest rates are seen in Andean Latin America (342.51), Tropical Latin America (471.19), and Central Latin America (472.02). On the other hand, the highest rates for ASIR are observed in Central Asia (1,093.93), North Africa and Middle East (1,049.69), and Eastern Europe (1,007.70) (Supplementary Tables 1–3; Figure 1).

When analyzing the trends in disease burden from 2020 to 2030, it is clear that Southern Sub-Saharan Africa has the lowest EAPC for age-standardized DALY rate at −5.75, followed by Eastern Europe at −3.28, and Central Asia at −2.03. Conversely, the region with the highest EAPC for age-standardized DALY rate is High-income Asia Pacific at 0.23. In terms of EAPC for ASDR, Southern Sub-Saharan Africa shows the lowest rate at −6.63, followed by Eastern Europe at −2.62, and Central Asia at −2.49. On the other hand, the highest EAPC for ASDR is seen in High-income Asia Pacific at 0.55, followed by High-income North America at 0.17. Lastly, for EAPC of ASIR, the regions with the lowest rates are Western Europe at −0.22, Central Asia at −0.15, and Eastern Europe at −0.08. Conversely, the highest EAPC for ASIR is observed in High-income Asia Pacific at 0.78, Southern Latin America at 0.69, and the Caribbean at 0.43 (Table 1, Supplementary Tables 4, 5, and Figure 2).

**Figure 3:**
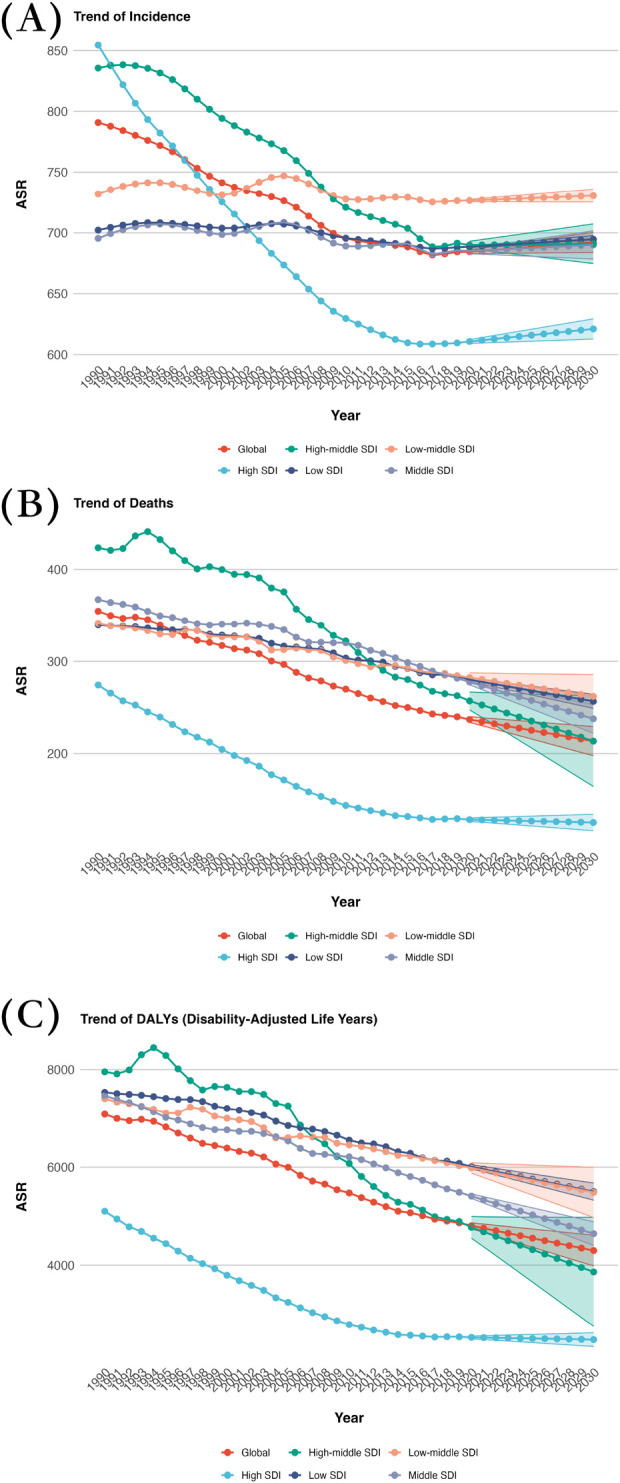
Projected global burden trend of CVD from 2020 to 2030, by SDI regions. **(A)** ASIR **(B)** ASDR **(C)** Age-standardized DALY rate. DALY, disability adjusted life-year; ASIR, age standardized incidence rate; ASDR, age standardized death rate.

Projections for CVD between 2020 and 2030 across different countries show significant variations in age-standardized rate for DALYs, deaths, and incidence. Limpopo has the lowest burden with a rate of 736.38 for DALYs, followed by Eastern Cape with 933.38, and Mpumalanga with 985.63. In contrast, Solomon Islands has the highest DALYs at 21,342.70, followed by Kiribati with 13,583.75, and Vanuatu at 13,455.07 (Supplementary Tables 6, 7, Figures 3–S5).

**Figure 4:**
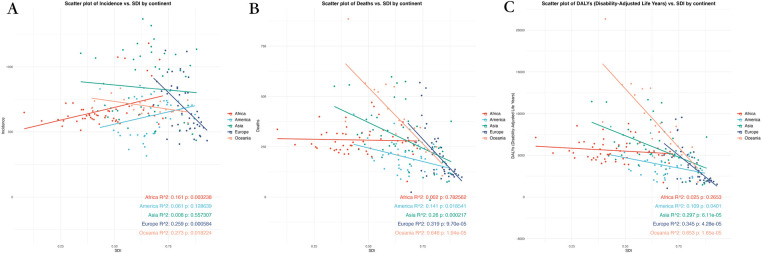
Scatter plot of correlation analysis between projected ASRs of global burden of CVD in 2030 and the level of SDI, by locations and sex. **(A)** ASIR **(B)** ASDR **(C)** Age-standardized DALY rate. DALY, disability adjusted life-year; ASIR, age standardized incidence rate; ASDR, age standardized death rate; ASRs, age standardized rates.

In terms of ASDR, Oman has the lowest rate at −15.59, with Limpopo at 8.32 and Republic of Moldova at 24.00 following closely. Conversely, Solomon Islands has the highest ASDR at 885.13, with Mongolia at 597.69, and Turkmenistan at 574.34 trailing behind. When examining ASIR, Peru has the lowest rate at 316.41, followed by Bolivia (Plurinational State of) at 364.90, and Ecuador at 384.93. On the other hand, Ardebil has the highest incidence rate at 1,429.77, with North Khorasan and Khuzestan not far behind at 1,425.34 and 1,420.53, respectively (Supplementary Tables 8, 9 and Figures 3–5).

The Republic of Moldova has shown the most significant reduction in burden in terms of age-standardized DALY rate, with a negative change of −15.70, while Cabo Verde has exhibited a notable positive trend at 4.08. When analyzing ASDR, Oman has demonstrated the most substantial decline with an EAPC of −22.98, and Cabo Verde once again leads with the highest EAPC for deaths at 4.82. Looking at ASIR, Germany has experienced the most significant decrease with an EAPC of −2.47, while Slovenia shows the greatest increase at 1.80 (Supplementary Tables 10, 11 and Figures 3–5).

These results underscore a stark geographic disparity in CVD burden, with the highest burden concentrated in Oceania, Central Asia, and parts of Eastern Europe, while High-income regions experience the lowest levels across all three metrics.

### 3.4 Projection for the distribution and of correlation analysis of CVD in different levels of SDI, 2020–2030

In 2030, the regions with low-middle SDI showed the highest ASDR at 261.99, while High SDI regions had the lowest ASDR at 125.25. Furthermore, Low-middle SDI regions also had the highest ASIR at 730.66, compared to High SDI regions with the lowest ASIR at 621.03. Regarding age-standardized DALYs rate, Low SDI regions had the highest rate of 5,501.81, whereas High SDI regions had the lowest rate at 2,475.25 (Supplementary Table 1; Figures 1,3).

In terms of EAPC, regions with Low-middle SDI show the highest increase in ASIR at 0.1003. When it comes to EAPC for ASDR, Low-middle SDI regions experience the lowest decrease at −0.87, whereas High SDI regions have the highest increase at −0.18. Regarding EAPC for age-standardized DALY rate, the highest decrease is observed in High SDI regions at −0.25, while the lowest decrease is seen in Low-middle SDI regions at −0.87 (Table 1; Figures 1,3).

The scatter plots for 2030 reveal insights into the relationship between SDI and CVD burden across five continents. The data shows that regions with higher SDI generally have lower CVD burden, supported by significant correlations. Specifically, Europe (*R*^2^ = 0.259, *p* < 0.05) and Oceania (*R*^2^ = 0.273, *p* < 0.05) both demonstrate a negative correlation with SDI, indicating that higher SDI is associated with lower ASIR. This trend is in line with the expected impact of improved socio-economic conditions and healthcare infrastructure on reducing incidence rates.

In terms of deaths, Oceania exhibits the most pronounced negative correlation with SDI (*R*^2^ = 0.646, *p* < 0.05), suggesting that regions with higher SDI tend to have lower CVD-related mortality rates. Likewise, Europe demonstrates a significant negative correlation (*R*^2^ = 0.319, *p* < 0.05), supporting the observation that higher SDI levels are linked to decreased death rates. When considering DALYs, Oceania once again shows the strongest correlation with SDI (*R*^2^ = 0.653, *p* < 0.05), indicating that higher SDI values are associated with a notable decrease in the overall disease burden. Europe (*R*^2^ = 0.345, *p* < 0.05) and Asia (*R*^2^ = 0.297, *p* < 0.05) also display a similar pattern, suggesting that regions with higher SDI generally have lower DALYs, reflecting potentially better healthcare outcomes (Figure 4; Supplementary Figures 6, 7).

Overall, an inverse relationship between SDI and CVD burden is evident, with lower-SDI regions facing a disproportionately high and growing burden, both in absolute rates and projected trends.

## 4 Discussion

The study forecasts a rise in the global burden of CVD from 2020 to 2030, with significant increases in ASIR, while the ASDR and age-standardized DALY rate are predicted to decrease. This suggests improvements in disease outcomes, despite an increasing number of cases. By 2030, males are expected to bear a higher burden of CVD compared to females, particularly in terms of mortality rates and DALYs. The Oceania region is projected to experience a notably higher burden of CVD compared to other regions, with countries like Kiribati, Turkmenistan, and Mongolia showing especially high ASIR. Correlation analysis indicates a consistent negative relationship between SDI and the burden of CVD.

The projected increase in the ASIR of CVD may be attributed to various factors, such as the global shift towards sedentary lifestyles and high-calorie diets, leading to higher incidence rates (21). These lifestyle changes are particularly evident in rapidly urbanizing regions, resulting in a surge in CVD cases. On the other hand, the projected decreases in deaths and DALYs could be linked to advancements in medical technology and improvements in healthcare services (22, 23). The World Health Organization emphasizes that significant lifestyle changes can reduce the incidence of CVD by 80%, and even 40% of cancer incidence (24). The 2019 ACC/AHA guidelines aim to serve as a reference for community, clinical, and public health practices, with a focus on primary prevention of adult CVD and the outcomes of atherosclerotic cardiovascular disease. The guidelines offer recommendations on lifestyle factors like diet and physical activity, as well as other risk factors for cardiovascular disease such as obesity, diabetes, blood cholesterol, hypertension, smoking, and aspirin use. Additionally, the guidelines highlight patient-centered approaches including team care, shared decision making, and evaluation of health-related social determinants (25).

The optimal prevention of CVD should be implemented from pregnancy through the entire lifespan. In clinical settings, CVD prevention typically targets individuals with existing CVD (secondary prevention) or those at high risk of experiencing major cardiovascular events due to factors like smoking, high blood pressure, diabetes, or dyslipidemia (primary prevention). However, current practices often overlook young or elderly populations with moderate or mild CVD risk, and enhancing CVD prevention strategies in these groups could yield greater benefits (26).

The trends for DALYs, a measure of overall disease burden, indicate a decrease in all SDI regions. The most significant decline is observed in high SDI regions, suggesting a substantial reduction in the impact of CVD. Lower SDI regions also show a steady decrease, albeit at a slower pace. The global trend for DALYs reflects this pattern, with a consistent decline over the past decade, demonstrating that efforts to reduce the burden of CVD are producing positive outcomes.

An analysis of the burden of CVD by gender reveals that by 2030, males are expected to bear a significantly higher burden compared to females. This gender disparity in CVD burden may be linked to cultural factors and risky behaviors. For example, in Central Asia, males are more likely to engage in high-risk behaviors such as smoking and heavy alcohol consumption (27), which contribute to the increased burden of CVD in this population. Furthermore, occupational hazards could also be a contributing factor to the higher incidence rates among males, particularly in regions where they are more likely to work in physically demanding or hazardous environments (28). In addition to behavioral and occupational factors, biological and physiological differences may partly explain the higher CVD burden in males. For instance, premenopausal women benefit from the cardioprotective effects of estrogen, which helps regulate lipid metabolism, endothelial function, and inflammatory response (29). Men, by contrast, tend to develop atherosclerosis earlier and exhibit higher baseline levels of certain cardiovascular risk factors such as visceral adiposity and blood pressure (30). Socio-cultural and healthcare access factors also play important roles. Men are generally less likely to participate in preventive healthcare, undergo routine screening, or adhere to treatment regimens, which may delay diagnosis and contribute to worse outcomes. In some healthcare settings, symptoms in women may be under-recognized or misattributed (e.g., non-classic chest pain), leading to potential diagnostic and treatment bias that could paradoxically reduce observed female burden. Together, these multidimensional factors highlight the complexity of gender disparities in CVD and underscore the need for gender-sensitive prevention and intervention strategies (31).

The analysis of CVD disparities between 2020 and 2030 could benefit from deeper exploration of the socioeconomic, cultural, and structural drivers behind observed differences. For instance, while the Solomon Islands, Kiribati, and Vanuatu exhibit high ASDR and age-standardized DALY rates, these outcomes are not merely due to geographic isolation or dietary shifts but reflect systemic inequities. These Pacific Island nations face unique challenges: their remote locations exacerbate supply chain disruptions, limiting access to affordable fresh produce, while globalization has eroded traditional food systems (30, 31). Historical reliance on local diets rich in fish, tubers, and coconuts has been supplanted by imported, ultra-processed foods high in sodium, sugar, and trans fats—linked to rising obesity and diabetes rates (e.g., Kiribati's adult obesity prevalence now exceeds 50%). In contrast, Peru and Ecuador maintain lower CVD incidence partly due to policy-driven protection of indigenous agricultural practices (32). Peru's promotion of quinoa, potatoes, and legumes through school meal programs and national campaigns reinforces cardioprotective diets, while Ecuador's community-based health initiatives integrate traditional knowledge with modern prevention strategies (33).

The economic dimensions of these disparities are also underexplored. Cabo Verde's high EAPC (estimated annual percentage change) correlates with its heavy dependence on food imports (over 80% of staple foods), driven by climate vulnerability and limited arable land, whereas Moldova's declining burden stems from targeted interventions like tobacco taxation, salt reduction campaigns, and expanded rural healthcare access post-2010 health system reforms (34). Notably, urbanization patterns further amplify contrasts: rapid, unplanned urban growth in Pacific Island capitals concentrates poverty and unhealthy food environments, while Andean countries' slower urbanization preserves rural dietary traditions. Addressing these inequities requires nuanced strategies—such as improving cold-chain logistics for fresh food distribution in island nations, regulating transnational food corporations in import-dependent economies, and scaling community health worker models from successful regions. Without such tailored approaches, global CVD disparities will likely widen, reflecting deeper inequities in resource allocation and policy prioritization (35).

The trends in CVD from 2020 to 2030 show significant variations across regions categorized by SDI. High-SDI regions, such as High-income Asia Pacific, benefit from robust healthcare infrastructure and public health campaigns, enabling effective adoption of interventions like dietary counseling and lipid-lowering therapies recommended by the ACC/AHA guidelines (28, 36). In contrast, low-SDI regions (e.g., Oceania, Central Asia) face barriers such as limited healthcare access, economic constraints, and reliance on imported unhealthy foods, which hinder guideline implementation (30). For instance, Cabo Verde's rising CVD burden correlates with economic challenges and dietary shifts toward processed foods, underscoring the need for subsidized healthy food programs and community-based education (35). High-SDI regions could focus on optimizing risk stratification tools and digital health interventions, while low-SDI areas require investments in primary care infrastructure and culturally adapted messaging to address behavioral risk factors (e.g., tobacco use in Central Asia). Tailored strategies, such as integrating CVD prevention into maternal health programs in low-SDI settings, could address disparities in preventive care uptake (36). Future research should prioritize evaluating region-specific implementation models to bridge these gaps (37).

This study provides a comprehensive analysis of the burden of CVD across all age groups, using data from 1990 to 2019 to project future trends up to 2030. The research covers various demographic variables including gender, countries, regions, and socio-economic statuses, offering a global perspective on CVD (35). The analysis was rigorous, incorporating multiple intergroup tests to ensure the robustness of the findings. This study has several limitations. First, the analysis did not adjust for key cardiovascular health determinants, including behavioral (e.g., smoking, physical inactivity), metabolic (e.g., hypertension, diabetes), and socioeconomic risk factors (e.g., income inequality, education access), which could confound the observed associations between SDI and CVD burden. These unaccounted variables may partially explain regional disparities in CVD trends and limit the precision of projections. Second, the use of GBD data introduces inherent constraints. In low-SDI countries, limited registry coverage and fragmented surveillance systems may lead to underreporting of CVD cases and deaths. For instance, many regions in Central Asia and Oceania lack standardized diagnostic protocols or population-based registries, resulting in inconsistent case definitions and potential misclassification of CVD subtypes via ICD codes (35). This could underestimate mild or asymptomatic cases, particularly in areas with poor healthcare accessibility. Furthermore, GBD estimates for these regions often rely on extrapolation from sparse data sources, amplifying uncertainty in trend projections. Third, unmeasured sociocultural factors—such as dietary transitions toward processed foods in Cabo Verde or occupational hazards in male-dominated industries—may influence CVD outcomes but are not fully captured in the GBD framework. These gaps highlight the need for cautious interpretation of results, especially when informing policy decisions in resource-limited settings (37).

Future research should focus on: (1) Elucidating the mechanisms underlying gender disparities (e.g., biological, behavioral, and socio-cultural factors contributing to higher CVD incidence in males in regions like Central Asia); (2) Conducting dynamic analyses of environmental and behavioral risk factors in low SDI regions (e.g., Oceania, Sub-Saharan Africa) to inform culturally adaptable prevention strategies; (3) Developing intelligent prevention and control technologies (e.g., AI-assisted community screening) tailored to resource-limited settings; (4) Establishing climate-population-disease coupled monitoring systems to track epidemiological shifts in high-burden areas (e.g., Central Asia, small island nations); and (5) Advancing multi-omics integration and health policy synergy research (e.g., precision intervention strategies for high-incidence regions like Eastern Europe) (38). These directions will provide targeted scientific support for global CVD prevention and control.

These findings underscore the urgency of prioritizing tailored, equity-focused interventions in high-risk regions. Structural investments in healthcare infrastructure, coupled with policies targeting dietary shifts and risk factor mitigation, are CItical to reducing disparities. For low-SDI settings, integrating CVD prevention into poverty reduction programs and enhancing access to preventive care via telemedicine and community health workers could yield significant gains. In regions with rising EAPCs, dynamic surveillance systems and regional collaborations are essential to rapidly adapt guidelines and allocate resources effectively (32). Gender-specific interventions—particularly for men in Central Asia and Eastern Europe—should address occupational hazards and promote behavior change through culturally resonant messaging. Finally, global partnerships must prioritize translating evidence into localized action, ensuring that policy tools like food taxation and labeling frameworks are implemented alongside investments in social determinants of health to achieve sustainable reductions in the CVD burden (39).

## 5 Conclusion

The burden of CVD is projected to significantly increase from 1990 to 2030, with a greater impact on males than females. Regions such as Oceania are witnessing substantial rises in CVD incidence, deaths, and DALYs. Despite an expected decrease in deaths and DALYs, the increasing incidence rates highlight a growing number of CVD cases, emphasizing the need for targeted interventions. This study emphasizes the significance of addressing socio-economic, cultural, and geographic factors to reduce the global burden of CVD.

## Data Availability

The original contributions presented in the study are included in the article/Supplementary Material, further inquiries can be directed to the corresponding authors.
