# Supplementary material for: A global prediction of cardiovascular disease from 2020 to 2030
Source: Front Cardiovasc Med. 2025 Aug 11;12:1462705. doi: 10.3389/fcvm.2025.1462705 (PMC12375611; doi:10.3389/fcvm.2025.1462705)

SFigure 1:Projected ASRs of global burden of CVD in 2030, by locations. (A) ASIR (B) ASDR (C) Age-standardized DALY rate. DALY = disability adjusted life-year. ASIR = age standardized incidence rate. ASDR = age standardized death rate. ASRs = age standardized rates.

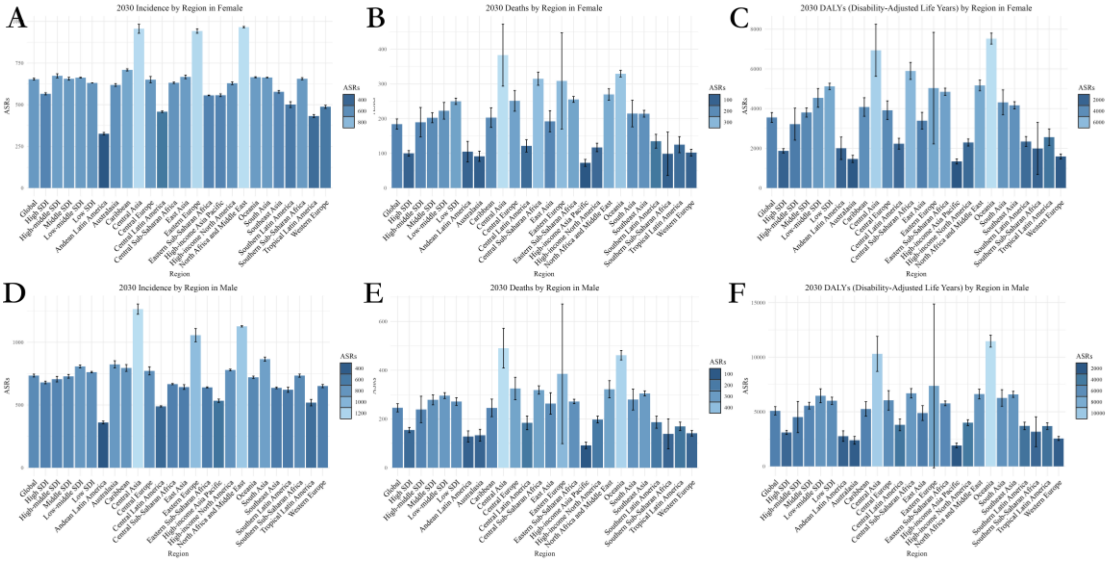

SFigure 2:Projected ASRs of global burden of CVD in 2030, by locations and genders. (A) ASIR (B) ASDR (C) Age-standardized DALY rate. DALY = disability adjusted life-year. ASIR = age standardized incidence rate. ASDR = age standardized death rate. ASRs = age standardized rates.

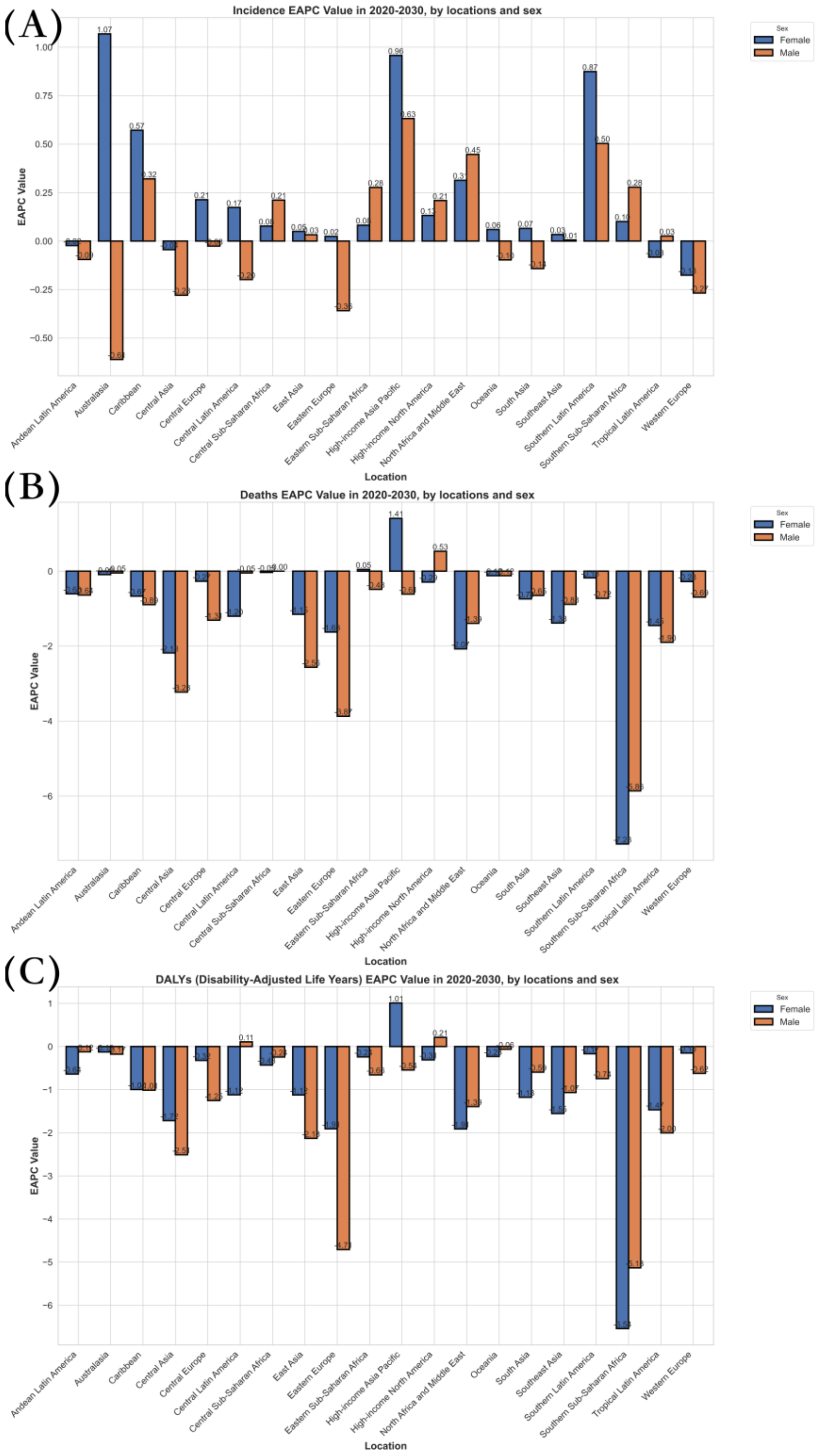

SFigure 3: Projected ASRs of global burden of CVD of both genders in 204 countries in 2030, by locations. (A) ASIR (B) ASDR (C) Age-standardized DALY rate. DALY = disability adjusted life-year. ASIR = age standardized incidence rate. ASDR = age standardized death rate. ASRs = age standardized rates.

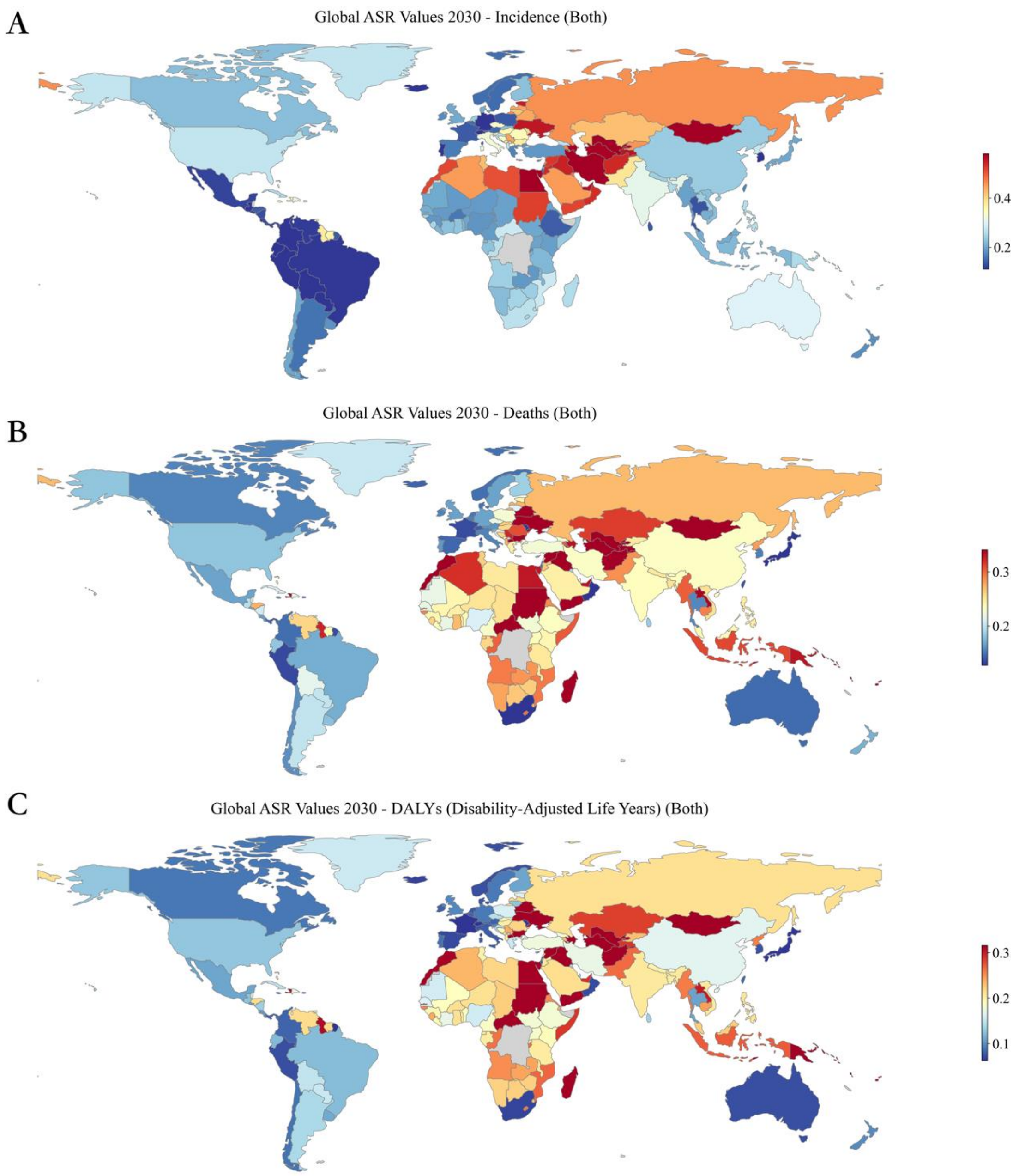

SFigure 4: Projected ASRs of global burden of CVD of male in 204 countries in 2030, by locations. (A) ASIR (B) ASDR (C) Age-standardized DALY rate. DALY = disability adjusted life-year. ASIR = age standardized incidence rate. ASDR = age standardized death rate. ASRs = age standardized rates.

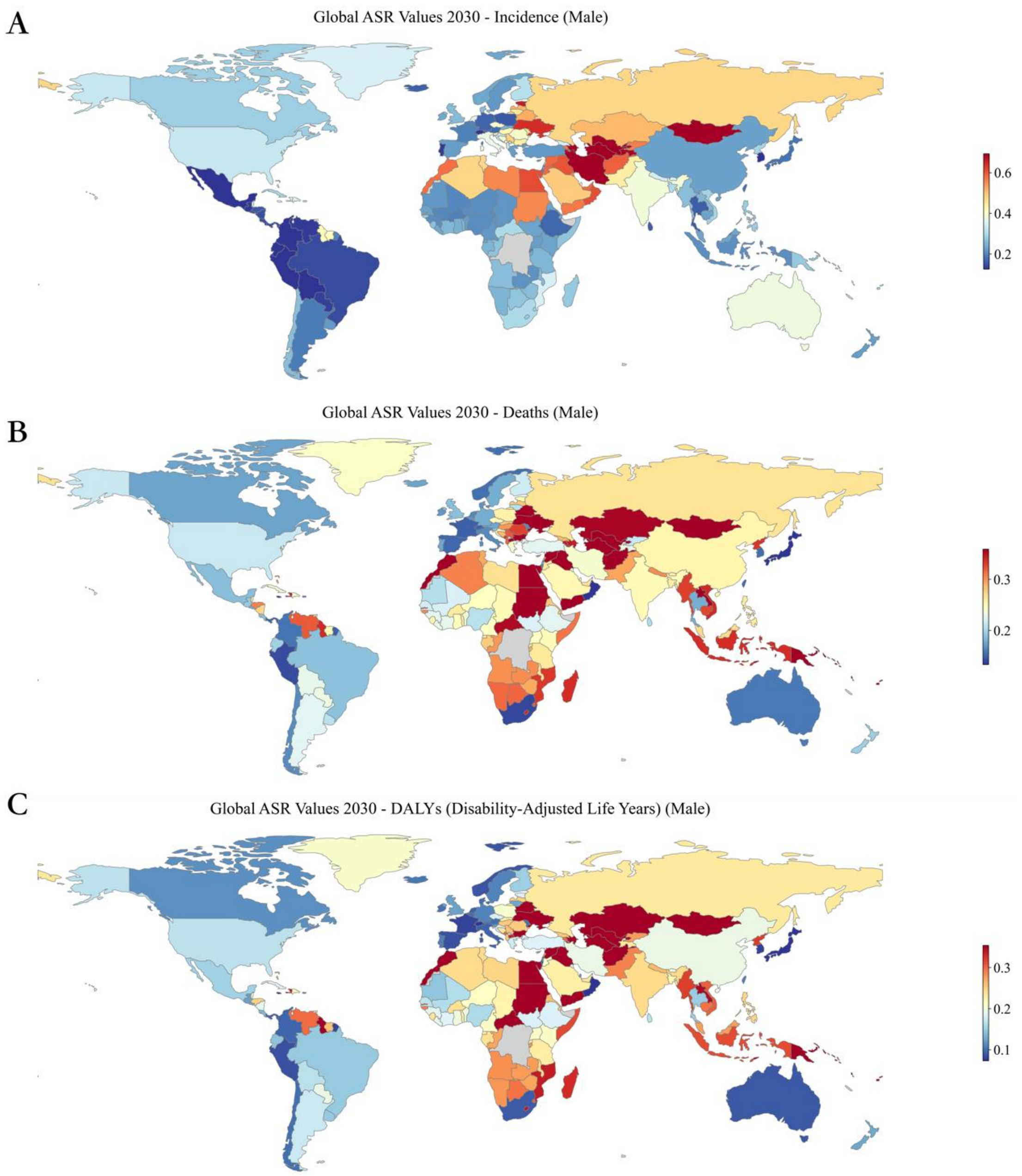

SFigure 5: Projected ASRs of global burden of CVD of female in 204 countries in 2030, by locations. (A) ASIR (B) ASDR (C) Age-standardized DALY rate. DALY = disability adjusted life-year. ASIR = age standardized incidence rate. ASDR = age standardized death rate. ASRs = age standardized rates.

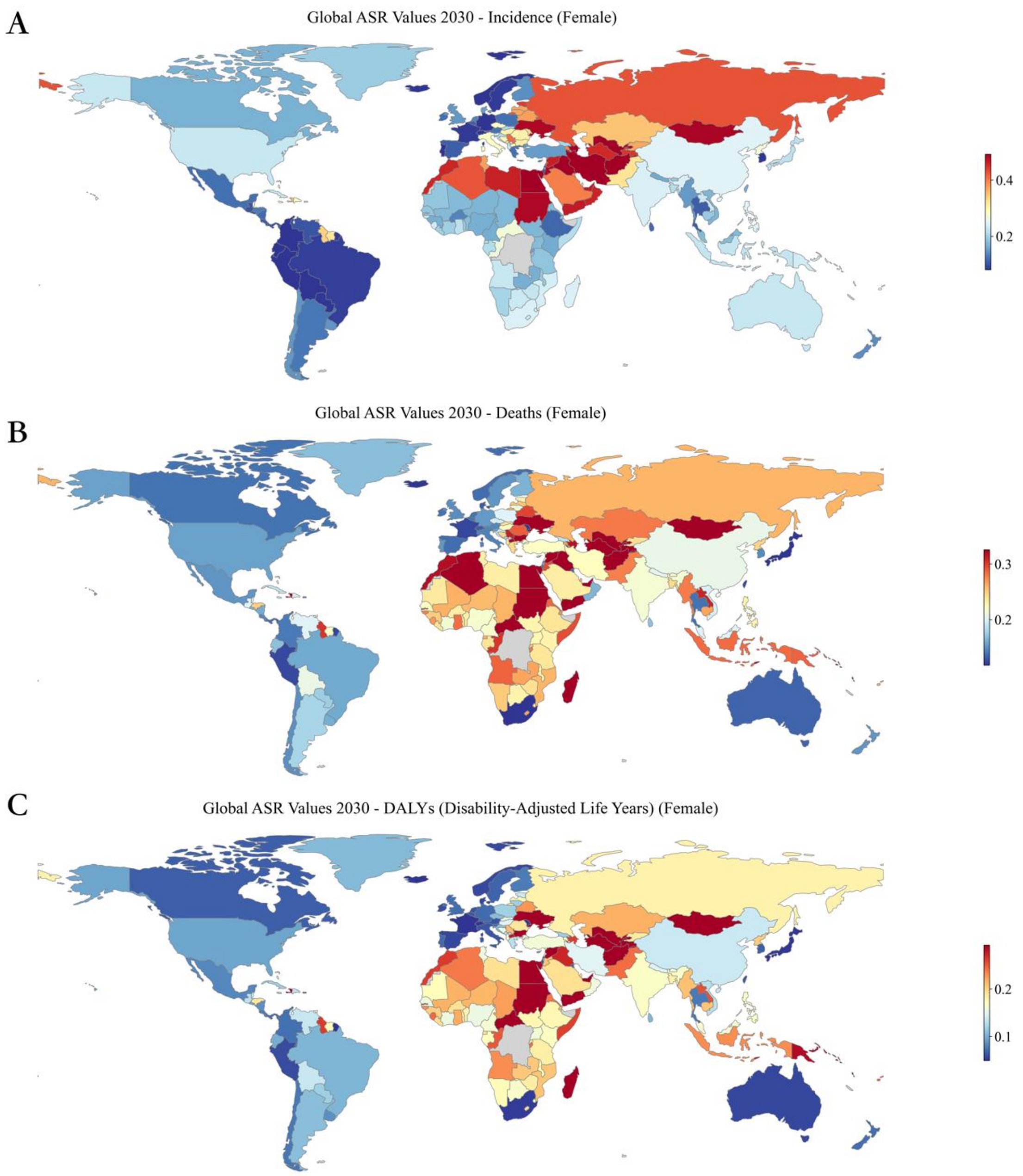

SFigure 6: Scatter plot of correlation analysis between projected ASRs of global burden of CVD in 2030 and the level of SDI of male, by locations. (A) ASIR (B) ASDR (C) Age-standardized DALY rate. DALY = disability adjusted life-year. ASIR = age standardized incidence rate. ASDR = age standardized death rate. ASRs = age standardized rates.

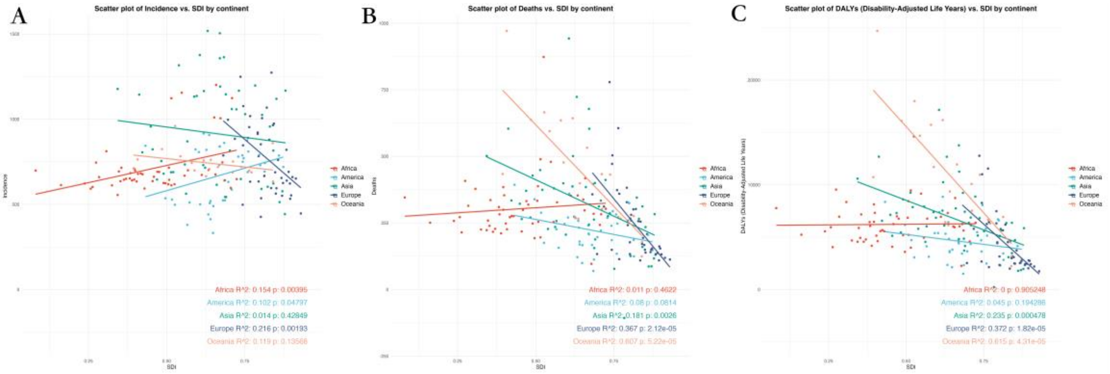

SFigure 7: Scatter plot of correlation analysis between projected ASRs of global burden of CVD in 2030 and the level of SDI of female, by locations. (A) ASIR (B) ASDR (C) Age-standardized DALY rate. DALY = disability adjusted life-year. ASIR = age standardized incidence rate. ASDR = age standardized death rate. ASRs = age standardized rates.

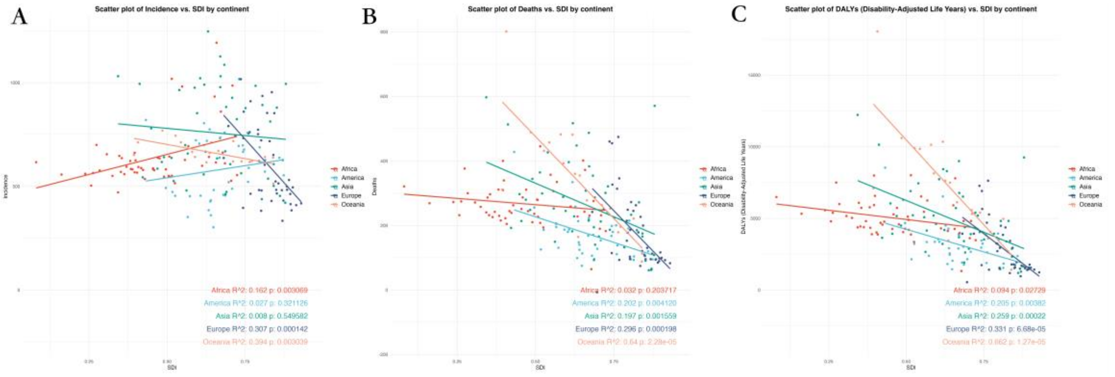

Supplement: Supplementary file 1 [file Datasheet1.pdf]
