# Supplementary material for: A global prediction of cardiovascular disease from 2020 to 2030
Source: Front Cardiovasc Med. 2025 Aug 11;12:1462705. doi: 10.3389/fcvm.2025.1462705 (PMC12375611; doi:10.3389/fcvm.2025.1462705)
Supplement: Supplementary file 2 [file Datasheet2.pdf]

STable 1. The projected age-standardized rates of CVD in 2030 in different regions.

location	Death(95 %UI)	Incidence(95 %UI)	Disability-Adjusted Life Years (DALYs)(95%UI)	Death(95%UI)		Incidence(95%UI)		DALYs (95%UI)	
				Male	Female	Male	Female	Male	Female
Global	213.43 (197.59, 229.28)	692.18 (683.90, 700.47)	4297.80 (3984.01, 4611.59)	245.92 (229.12, 262.73)	184.33 (169.49, 199.16)	5092.65 (4712.68, 5472.63)	3553.02 (3302.86, 3803.19)	734.72 (723.22, 746.21)	653.71 (647.71, 659.70)
High-middle SDI	213.50 (164.29, 262.70)	691.04 (674.74, 707.33)	3861.59 (2745.72, 4977.46)	239.16 (184.97, 293.34)	189.58 (147.04, 232.13)	4520.31 (3090.46, 5950.16)	3218.05 (2413.25, 4022.85)	706.29 (684.14, 728.44)	673.58 (661.15, 686.00)
High SDI	125.25 (116.36, 134.15)	621.03 (612.71, 629.34)	2475.25 (2335.54, 2614.96)	154.69 (143.93, 165.46)	99.86 (91.86, 107.86)	3115.62 (2929.79, 3301.45)	1874.26 (1764.30, 1984.23)	679.78 (669.18, 690.37)	565.47 (559.18, 571.75)
Low-middle SDI	261.99 (238.32, 285.66)	730.66 (725.39, 735.92)	5485.88 (4973.49, 5998.27)	295.96 (283.92, 308.01)	222.68 (198.69, 246.68)	6469.32 (5827.84, 7110.80)	4538.88 (4065.30, 5012.47)	807.05 (795.09, 819.01)	662.83 (660.50, 665.16)
Low SDI	256.61 (249.01, 264.21)	694.63 (691.51, 697.75)	5501.81 (5327.52, 5676.09)	271.50 (255.66, 287.34)	249.99 (240.90, 259.07)	6006.57 (5673.53, 6339.62)	5116.95 (4958.05, 5275.85)	762.02 (755.98, 768.06)	630.83 (630.24, 631.41)
Middle SDI	237.66 (222.04, 253.29)	690.02 (678.42, 701.63)	4642.86 (4399.64, 4886.08)	278.55 (258.05, 299.04)	202.63 (188.23, 217.03)	5563.70 (5255.35, 5872.05)	3803.40 (3574.06, 4032.74)	728.87 (713.89, 743.86)	656.27 (647.49, 665.05)
Andean Latin America	118.96 (93.78, 144.15)	342.51 (333.01, 352.00)	2400.87 (1928.49, 2873.26)	128.47 (105.84, 151.11)	104.72 (74.91, 134.52)	2773.59 (2293.25, 3253.93)	2003.04 (1437.96, 2568.12)	360.89 (349.51, 372.28)	326.02 (318.26, 333.78)

Australasia	111.69 (93.45, 129.94)	716.75 (704.49, 729.00)	1907.58 (1655.51, 2159.64)	133.38 (109.06, 157.70)	91.60 (76.87, 106.32)	2390.87 (2033.78, 2747.96)	1466.51 (1285.94, 1647.09)	824.21 (795.63, 852.79)	618.72 (610.49, 626.95)
Caribbean	226.26 (196.56, 255.97)	749.19 (733.39, 765.00)	4652.42 (4114.57, 5190.27)	245.58 (209.57, 281.58)	202.94 (174.63, 231.26)	5262.09 (4596.30, 5927.88)	4081.46 (3614.36, 4548.56)	795.10 (767.93, 822.26)	709.24 (702.88, 715.60)
Central Asia	437.48 (349.87, 525.09)	1093.93 (1061.18, 1126.67)	8529.09 (7072.67, 9985.51)	490.46 (409.01, 571.92)	382.80 (293.65, 471.96)	10304.83 (8698.16, 11911.50)	6936.08 (5624.25, 8247.92)	1264.07 (1223.16, 1304.98)	956.16 (929.06, 983.25)
Central Europe	287.82 (252.05, 323.59)	709.76 (685.29, 734.22)	4969.53 (4330.08, 5608.98)	325.00 (278.90, 371.11)	251.14 (221.34, 280.95)	6048.98 (5158.38, 6939.58)	3918.18 (3446.28, 4390.08)	772.21 (739.60, 804.81)	650.49 (631.23, 669.74)
Central Latin America	149.43 (129.49, 169.36)	472.02 (467.35, 476.68)	2959.70 (2601.38, 3318.02)	184.06 (156.05, 212.07)	121.45 (103.77, 139.12)	3801.38 (3265.07, 4337.69)	2233.56 (1962.01, 2505.12)	489.04 (484.26, 493.82)	458.21 (453.49, 462.92)
Central Sub-Saharan Africa	321.10 (303.62, 338.57)	648.09 (644.84, 651.34)	6314.86 (5904.97, 6724.75)	318.92 (301.79, 336.06)	315.14 (296.09, 334.20)	6696.78 (6264.05, 7129.51)	5897.92 (5469.02, 6326.82)	666.58 (661.79, 671.38)	631.84 (627.49, 636.18)
East Asia	229.00 (194.22, 263.78)	655.02 (641.73, 668.30)	4136.41 (3616.30, 4656.53)	263.49 (219.33, 307.66)	192.41 (162.09, 222.73)	4884.77 (4203.44, 5566.09)	3392.23 (2972.97, 3811.50)	643.18 (624.24, 662.12)	667.24 (656.38, 678.10)
Eastern Europe	343.80 (146.00, 541.61)	1007.70 (981.14, 1034.26)	6171.90 (1368.89, 10974.91)	385.23 (98.90, 671.57)	308.65 (170.14, 447.16)	7374.91 (-124.95, 14874.77)	5030.64 (2221.73, 7839.55)	1056.50 (1003.72, 1109.28)	942.54 (929.26, 955.82)
Eastern Sub-Saharan	264.73 (256.03,	595.39 (592.91,	5307.32 (5109.83, 5504.81)	271.89 (262.85,	255.37 (246.89,	5771.95 (5555.40,	4847.63 (4665.20,	640.62 (636.64,	556.15 (554.27,

Africa	273.43)	597.87)		280.93)	263.85)	5988.49)	5030.06)	644.59)	558.04)
High-income Asia Pacific	84.07 (72.46, 95.68)	546.12 (536.04, 556.20)	1643.58 (1480.22, 1806.94)	92.03 (78.87, 105.20)	72.50 (62.45, 82.55)	1918.52 (1707.10, 2129.94)	1336.03 (1209.00, 1463.06)	534.18 (521.01, 547.35)	557.02 (548.77, 565.27)
High-income North America	153.66 (140.98, 166.35)	698.27 (691.12, 705.42)	3092.88 (2877.83, 3307.93)	197.77 (183.68, 211.86)	116.97 (104.79, 129.16)	4006.16 (3733.80, 4278.53)	2292.34 (2117.01, 2467.66)	779.26 (772.55, 785.96)	629.01 (620.73, 637.29)
North Africa and Middle East	296.88 (272.55, 321.20)	1049.69 (1045.48, 1053.90)	5922.74 (5579.15, 6266.33)	322.02 (286.88, 357.17)	269.47 (253.08, 285.86)	6624.72 (6176.40, 7073.03)	5163.45 (4894.35, 5432.56)	1126.91 (1121.83, 1131.98)	966.06 (962.03, 970.09)
Oceania	397.03 (383.71, 410.35)	694.00 (688.27, 699.73)	9556.79 (9155.73, 9957.85)	461.54 (442.30, 480.79)	329.43 (319.76, 339.10)	11470.51 (10923.86, 12017.17)	7525.88 (7250.78, 7800.98)	721.40 (712.23, 730.57)	665.03 (661.87, 668.20)
South Asia	246.92 (206.09, 287.76)	761.46 (753.33, 769.59)	5287.82 (4615.74, 5959.89)	279.82 (236.82, 322.82)	214.77 (176.33, 253.21)	6268.51 (5510.85, 7026.17)	4314.17 (3686.30, 4942.04)	865.52 (849.01, 882.04)	663.34 (661.55, 665.13)
Southeast Asia	256.07 (246.18, 265.96)	606.07 (598.85, 613.28)	5318.04 (5094.36, 5541.71)	304.95 (294.41, 315.50)	214.19 (204.48, 223.90)	6599.30 (6321.49, 6877.11)	4165.53 (3982.72, 4348.34)	636.79 (630.17, 643.41)	577.79 (570.13, 585.46)
Southern Latin America	159.77 (138.61, 180.93)	558.08 (538.98, 577.18)	2989.66 (2703.63, 3275.69)	186.93 (162.21, 211.64)	134.94 (115.10, 154.79)	3705.78 (3362.51, 4049.06)	2344.21 (2093.36, 2595.06)	621.83 (600.94, 642.71)	500.78 (483.17, 518.39)
Southern Sub-Saharan Africa	116.20 (57.66, 174.74)	690.16 (685.84, 694.48)	2532.24 (1280.88, 3783.60)	138.99 (77.44, 200.54)	98.74 (36.18, 161.29)	3176.21 (1807.09, 4545.33)	1991.62 (680.26, 3302.99)	734.20 (721.59, 746.80)	656.20 (649.72, 662.68)
Tropical	144.41	471.19	3085.28 (2768.55,	169.88	125.05	3691.18	2558.94	518.91	432.30

Latin America	(128.82, 159.99)	(454.56, 487.81)	3402.01)	(152.61, 187.15)	(101.82, 148.28)	(3389.59, 3992.77)	(2138.50, 2979.39)	(493.10, 544.72)	(423.14, 441.46)
Western Europe	121.28 (110.76, 131.80)	566.82 (555.82, 577.82)	2061.55 (1909.62, 2213.49)	141.63 (130.12, 153.15)	102.01 (92.19, 111.84)	2558.25 (2376.02, 2740.47)	1587.90 (1459.40, 1716.40)	650.97 (638.12, 663.81)	487.70 (478.07, 497.32)

STable 2. Buttom 10 regions of the projected age-standardized rates of CVD in 2030.

Rank	DALYs Country (95%UI)	DALYs Value(95%UI)	Deaths Country	Deaths Value(95%UI)	Incidence Country	Incidence Value(95%UI)
1	High-income Asia Pacific	1643.58 (1480.22, 1806.94)	High-income Asia Pacific	84.07 (72.46, 95.68)	Andean Latin America	342.51 (333.01, 352.00)
2	Australasia	1907.58 (1655.51, 2159.64)	Australasia	111.69 (93.45, 129.94)	Tropical Latin America	471.19 (454.56, 487.81)
3	Western Europe	2061.55 (1909.62, 2213.49)	Southern Sub-Saharan Africa	116.20 (57.66, 174.74)	Central Latin America	472.02 (467.35, 476.68)
4	Andean Latin America	2400.87 (1928.49, 2873.26)	Andean Latin America	118.96 (93.78, 144.15)	High-income Asia Pacific	546.12 (536.04, 556.20)
5	Southern Sub-Saharan Africa	2532.24 (1280.88, 3783.60)	Western Europe	121.28 (110.76, 131.80)	Southern Latin America	558.08 (538.98, 577.18)
6	Central Latin America	2959.70 (2601.38, 3318.02)	Tropical Latin America	144.41 (128.82, 159.99)	Western Europe	566.82 (555.82, 577.82)
7	Southern Latin America	2989.66 (2703.63, 3275.69)	Central Latin America	149.43 (129.49, 169.36)	Eastern Sub-Saharan Africa	595.39 (592.91, 597.87)
8	Tropical Latin America	3085.28 (2768.55, 3402.01)	High-income North America	153.66 (140.98, 166.35)	Southeast Asia	606.07 (598.85, 613.28)
9	High-income North America	3092.88 (2877.83, 3307.93)	Southern Latin America	159.77 (138.61, 180.93)	Central Sub-Saharan Africa	648.09 (644.84, 651.34)
10	East Asia	4136.41 (3616.30, 4656.53)	Caribbean	226.26 (196.56, 255.97)	East Asia	655.02 (641.73, 668.30)

STable 3. Top 10 regions of the projected age-standardized rates of CVD in 2030.

Rank	DALYs Country	DALYs Value (95%UI)	Deaths Country	Deaths Value(95%UI)	Incidence Country	Incidence Value(95%UI)
1	Oceania	9556.79 (9155.73, 9957.85)	Central Asia	437.48 (349.87, 525.09)	Central Asia	1093.93 (1061.18, 1126.67)
2	Central Asia	8529.09 (7072.67, 9985.51)	Oceania	397.03 (383.71, 410.35)	North Africa and Middle East	1049.69 (1045.48, 1053.90)
3	Central Sub-Saharan Africa	6314.86 (5904.97, 6724.75)	Eastern Europe	343.80 (146.00, 541.61)	Eastern Europe	1007.70 (981.14, 1034.26)
4	Eastern Europe	6171.90 (1368.89, 10974.91)	Central Sub-Saharan Africa	321.10 (303.62, 338.57)	South Asia	761.46 (753.33, 769.59)
5	North Africa and Middle East	5922.74 (5579.15, 6266.33)	North Africa and Middle East	296.88 (272.55, 321.20)	Caribbean	749.19 (733.39, 765.00)
6	Southeast Asia	5318.04 (5094.36, 5541.71)	Central Europe	287.82 (252.05, 323.59)	Australasia	716.75 (704.49, 729.00)
7	Eastern Sub-Saharan Africa	5307.32 (5109.83, 5504.81)	Eastern Sub-Saharan Africa	264.73 (256.03, 273.43)	Central Europe	709.76 (685.29, 734.22)
8	South Asia	5287.82 (4615.74, 5959.89)	Southeast Asia	256.07 (246.18, 265.96)	High-income North America	698.27 (691.12, 705.42)
9	Central Europe	4969.53 (4330.08, 5608.98)	South Asia	246.92 (206.09, 287.76)	Oceania	694.00 (688.27, 699.73)
10	Caribbean	4652.42 (4114.57, 5190.27)	East Asia	229.00 (194.22, 263.78)	Southern Sub-Saharan Africa	690.16 (685.84, 694.48)

STable 4. Buttom 10 regions of the projected EAPC of age-standardized rates of CVD in 2030.

Rank	DALYs Country	DALYs Value (95%CI)	Deaths Country	Deaths Value(95%CI)	Incidence Country	Incidence Value(95%CI)
1	Southern Sub-Saharan Africa	-5.75 (-6.05, -5.44)	Southern Sub-Saharan Africa	-6.63 (-7.03, -6.22)	Western Europe	-0.22 (-0.22, -0.22)
2	Eastern Europe	-3.28 (-3.38, -3.18)	Eastern Europe	-2.62 (-2.68, -2.55)	Central Asia	-0.15 (-0.15, -0.15)
3	Central Asia	-2.03 (-2.07, -2.00)	Central Asia	-2.49 (-2.55, -2.44)	Eastern Europe	-0.08 (-0.08, -0.08)
4	Tropical Latin America	-1.74 (-1.77, -1.72)	Tropical Latin America	-1.72 (-1.74, -1.69)	South Asia	-0.07 (-0.07, -0.07)
5	North Africa and Middle East	-1.61 (-1.63, -1.59)	North Africa and Middle East	-1.69 (-1.72, -1.66)	Andean Latin America	-0.06 (-0.06, -0.06)
6	East Asia	-1.57 (-1.60, -1.55)	East Asia	-1.61 (-1.63, -1.58)	Tropical Latin America	-0.03 (-0.03, -0.03)
7	Southeast Asia	-1.28 (-1.29, -1.26)	Southeast Asia	-1.13 (-1.14, -1.12)	Oceania	-0.03 (-0.03, -0.03)
8	Caribbean	-0.99 (-1.00, -0.98)	South Asia	-0.68 (-0.68, -0.68)	Central Latin America	-0.01 (-0.01, -0.01)
9	South Asia	-0.84 (-0.84, -0.83)	Central Europe	-0.67 (-0.68, -0.67)	Southeast Asia	0.02 (0.02, 0.02)
10	Central Europe	-0.73 (-0.74, -0.73)	Caribbean	-0.67 (-0.67, -0.67)	East Asia	0.08 (0.08, 0.08)

STable 5. Top 10 regions of the projected EAPC of age-standardized rates of CVD in 2030.

Rank	DALYs Country	DALYs (Disability-Adjusted Life Years) Value(95%CI)	Deaths Country	Deaths Value(95%CI)	Incidence Country	Incidence Value(95%CI)
1	High-income Asia Pacific	0.23 (0.23, 0.23)	High-income Asia Pacific	0.55 (0.55, 0.55)	High-income Asia Pacific	0.78 (0.78, 0.79)
2	High-income North America	-0.02 (-0.02, -0.02)	High-income North America	0.17 (0.17, 0.17)	Southern Latin America	0.69 (0.69, 0.70)
3	Oceania	-0.13 (-0.13, -0.13)	Central Sub-Saharan Africa	-0.00 (-0.00, -0.00)	Caribbean	0.43 (0.43, 0.44)
4	Australasia	-0.17 (-0.17, -0.17)	Australasia	-0.04 (-0.04, -0.04)	North Africa and Middle East	0.39 (0.39, 0.39)
5	Andean Latin America	-0.27 (-0.27, -0.27)	Oceania	-0.11 (-0.11, -0.11)	Southern Sub-Saharan Africa	0.19 (0.19, 0.19)
6	Central Sub-Saharan Africa	-0.34 (-0.34, -0.34)	Eastern Sub-Saharan Africa	-0.21 (-0.21, -0.21)	Eastern Sub-Saharan Africa	0.18 (0.18, 0.18)
7	Western Europe	-0.38 (-0.38, -0.38)	Southern Latin America	-0.41 (-0.41, -0.41)	High-income North America	0.15 (0.15, 0.15)
8	Central Latin America	-0.43 (-0.43, -0.42)	Andean Latin America	-0.41 (-0.42, -0.41)	Central Sub-Saharan Africa	0.13 (0.13, 0.13)
9	Southern Latin America	-0.46 (-0.46, -0.45)	Western Europe	-0.43 (-0.43, -0.43)	Central Europe	0.12 (0.12, 0.12)
10	Eastern Sub-Saharan Africa	-0.46 (-0.46, -0.46)	Central Latin America	-0.63 (-0.63, -0.63)	Australasia	0.10 (0.10, 0.10)

STable 6. The projected age-standardized rates of CVD in 2030 in different countries, by genders.

	Deaths(95%UI)			DALYs (95%UI)			Incidence(95%UI)		
location	Male	Female	Both	Male	Female	Both	Male	Female	Both
Afghanistan	501.30 (468.71, 533.89)	597.25 (571.57, 622.93)	550.98 (521.82, 580.14)	10591.34 (9703.16, 11479.53)	12220.95 (11461.02, 12980.87)	11439.64 (10613.11, 12266.17)	1177.52 (1158.91, 1196.14)	1031.22 (1006.73, 1055.70)	1103.41 (1080.61, 1126.20)
Albania	357.43 (220.10, 494.75)	242.01 (170.29, 313.73)	294.17 (198.86, 389.48)	6257.86 (3988.35, 8527.38)	3928.23 (2805.45, 5051.01)	5030.70 (3421.64, 6639.76)	787.16 (779.96, 794.36)	636.78 (632.01, 641.56)	707.68 (702.59, 712.77)
Algeria	337.98 (315.56, 360.40)	401.25 (347.38, 455.12)	357.96 (324.56, 391.36)	6020.00 (5853.26, 6186.75)	5909.54 (5375.72, 6443.35)	5841.65 (5574.92, 6108.38)	1010.22 (995.81, 1024.64)	933.06 (914.87, 951.26)	971.23 (959.38, 983.07)
American Samoa	302.42 (285.47, 319.36)	246.81 (238.24, 255.38)	273.73 (262.92, 284.53)	7527.66 (7096.08, 7959.25)	5306.72 (5130.78, 5482.65)	6375.90 (6115.06, 6636.73)	750.68 (737.57, 763.79)	718.24 (714.65, 721.82)	734.75 (727.08, 742.42)
Andorra	109.36 (102.57, 116.15)	89.56 (83.35, 95.77)	100.01 (95.34, 104.67)	2029.20 (1921.10, 2137.29)	1441.89 (1341.81, 1541.97)	1745.45 (1680.43, 1810.46)	622.39 (613.21, 631.57)	463.03 (455.28, 470.77)	543.22 (535.09, 551.36)
Angola	331.98 (299.18, 364.79)	310.89 (281.95, 339.83)	319.93 (293.11, 346.76)	6990.93 (6084.86, 7896.99)	5727.64 (5023.66, 6431.62)	6237.33 (5535.91, 6938.76)	691.31 (687.87, 694.74)	638.05 (633.42, 642.68)	661.87 (657.95, 665.78)
Antigua and Barbuda	185.27 (39.31, 331.22)	198.44 (172.26, 224.63)	192.83 (124.13, 261.54)	3629.14 (1279.61, 5978.67)	3452.75 (3060.26, 3845.24)	3563.14 (2441.36, 4684.93)	750.32 (741.77, 758.87)	666.23 (659.60, 672.86)	709.05 (705.51, 712.59)
Argentina	221.13 (189.99,	153.78 (130.88,	185.55 (159.84,	4343.70 (3896.25,	2669.02 (2375.57,	3449.75 (3103.02,	586.59 (566.20,	488.46 (472.87,	535.65 (518.02,

	252.26)	176.67)	211.26)	4791.16)	2962.47)	3796.49)	606.99)	504.06)	553.29)
Armenia	399.88 (286.01, 513.75)	286.74 (176.86, 396.62)	335.37 (227.06, 443.69)	7428.66 (5590.25, 9267.07)	4379.96 (2689.75, 6070.17)	5777.60 (4062.48, 7492.72)	1145.72 (1126.74, 1164.70)	882.87 (866.83, 898.91)	999.05 (981.75, 1016.34)
Australia	124.20 (99.08, 149.32)	88.39 (73.10, 103.67)	105.71 (86.53, 124.89)	2232.64 (1859.03, 2606.25)	1419.48 (1231.82, 1607.13)	1809.60 (1542.11, 2077.09)	859.34 (830.18, 888.51)	638.65 (627.58, 649.72)	744.32 (732.97, 755.66)
Austria	149.47 (127.26, 171.68)	105.67 (90.39, 120.96)	126.85 (109.94, 143.75)	2489.35 (2153.75, 2824.96)	1534.38 (1319.09, 1749.67)	2007.34 (1764.46, 2250.23)	563.12 (521.78, 604.46)	482.90 (461.60, 504.19)	522.88 (489.68, 556.07)
Azerbaijan	395.49 (280.66, 510.32)	354.36 (239.50, 469.22)	376.14 (265.77, 486.50)	9221.81 (7544.19, 10899.43)	6517.79 (5093.74, 7941.84)	7819.22 (6331.27, 9307.17)	1363.90 (1345.85, 1381.95)	1086.17 (1070.57, 1101.77)	1212.44 (1195.91, 1228.96)
Bahamas	326.21 (261.89, 390.54)	190.96 (144.99, 236.92)	239.76 (195.24, 284.28)	6702.54 (5697.65, 7707.44)	3942.61 (3219.44, 4665.78)	5138.09 (4397.13, 5879.04)	790.65 (781.76, 799.53)	688.17 (684.36, 691.98)	735.15 (732.75, 737.55)
Bahrain	77.75 (4.96, 150.53)	223.27 (88.98, 357.57)	96.33 (43.84, 148.81)	5566.62 (1447.66, 9685.57)	3527.31 (1722.93, 5331.69)	4702.17 (1662.98, 7741.36)	1029.82 (1020.35, 1039.30)	877.51 (870.18, 884.84)	980.83 (971.41, 990.26)
Bangladesh	270.24 (168.86, 371.62)	248.34 (176.83, 319.85)	261.87 (177.97, 345.76)	5448.48 (4070.14, 6826.82)	4498.91 (3408.84, 5588.98)	5024.31 (3785.34, 6263.29)	754.04 (713.77, 794.30)	617.41 (601.97, 632.86)	683.05 (654.93, 711.17)
Barbados	239.34 (176.89, 301.79)	167.56 (149.24, 185.88)	201.29 (171.75, 230.82)	4710.42 (3746.80, 5674.05)	2961.74 (2620.26, 3303.21)	3750.07 (3252.95, 4247.19)	787.41 (773.72, 801.10)	697.42 (689.79, 705.04)	739.55 (734.64, 744.46)
Belarus	503.57	326.71	405.80	11004.48	5655.64	8051.77	1076.66	860.08	959.50

	(317.78, 689.37)	(221.33, 432.09)	(259.32, 552.28)	(5801.72, 16207.24)	(3684.81, 7626.47)	(4736.48, 11367.07)	(978.15, 1175.18)	(832.93, 887.24)	(905.89, 1013.11)
Belgium	138.74 (119.03, 158.44)	95.44 (81.13, 109.75)	115.98 (99.68, 132.28)	2474.95 (2159.55, 2790.34)	1568.19 (1368.02, 1768.36)	2013.78 (1774.64, 2252.92)	594.43 (579.39, 609.47)	465.45 (455.78, 475.12)	526.64 (515.53, 537.75)
Belize	270.16 (169.00, 371.32)	180.34 (127.65, 233.04)	221.27 (152.88, 289.67)	5389.02 (3679.96, 7098.07)	3545.54 (2696.53, 4394.55)	4433.52 (3283.57, 5583.47)	806.73 (789.15, 824.31)	705.90 (702.13, 709.67)	753.85 (746.19, 761.51)
Benin	239.05 (221.27, 256.84)	231.84 (217.50, 246.18)	235.88 (220.31, 251.45)	4932.87 (4598.67, 5267.07)	4436.05 (4176.93, 4695.17)	4682.12 (4397.71, 4966.54)	635.64 (632.04, 639.24)	569.74 (565.42, 574.05)	601.37 (597.33, 605.41)
Bermuda	186.41 (138.84, 233.99)	93.16 (76.78, 109.55)	136.75 (118.74, 154.76)	3440.06 (2539.66, 4340.46)	1540.67 (1305.25, 1776.10)	2453.82 (2113.44, 2794.21)	692.74 (678.28, 707.20)	570.95 (567.11, 574.79)	629.88 (622.81, 636.96)
Bhutan	264.69 (251.86, 277.52)	213.73 (205.67, 221.79)	237.00 (229.65, 244.36)	5132.95 (4826.79, 5439.10)	3920.19 (3701.92, 4138.46)	4502.85 (4307.71, 4697.98)	823.06 (821.63, 824.49)	582.65 (579.94, 585.35)	703.98 (702.54, 705.43)
Bolivia (Plurinational State of)	226.11 (214.92, 237.30)	195.25 (186.49, 204.01)	209.53 (201.93, 217.14)	4123.52 (3882.39, 4364.64)	3348.82 (3149.44, 3548.21)	3712.34 (3545.62, 3879.05)	379.29 (370.34, 388.25)	351.83 (343.80, 359.86)	364.90 (356.44, 373.35)
Bosnia and Herzegovina	311.44 (185.77, 437.12)	250.50 (74.69, 426.30)	271.29 (153.49, 389.09)	5801.62 (3721.81, 7881.43)	3932.78 (2228.85, 5636.71)	4772.59 (3206.68, 6338.49)	884.42 (878.89, 889.95)	745.71 (736.55, 754.87)	812.47 (805.43, 819.50)
Botswana	353.03 (330.38, 375.68)	227.79 (194.21, 261.36)	279.59 (259.19, 299.99)	7307.33 (6716.32, 7898.35)	4121.99 (3444.55, 4799.42)	5512.71 (5089.56, 5935.86)	702.04 (698.05, 706.03)	641.67 (632.79, 650.54)	668.12 (660.99, 675.26)

Brazil	168.86 (150.94, 186.79)	124.64 (101.66, 147.62)	142.97 (128.13, 157.81)	3667.41 (3367.53, 3967.28)	2551.83 (2135.54, 2968.11)	3039.75 (2752.70, 3326.80)	519.37 (493.12, 545.63)	432.64 (423.34, 441.93)	471.53 (454.64, 488.42)
Brunei Darussalam	345.16 (216.64, 473.69)	185.23 (128.11, 242.34)	242.53 (173.10, 311.96)	6350.58 (4604.26, 8096.91)	3390.10 (2746.07, 4034.13)	4714.26 (3798.68, 5629.84)	603.40 (595.70, 611.11)	537.05 (521.81, 552.29)	570.16 (558.87, 581.46)
Bulgaria	605.82 (412.71, 798.94)	474.30 (362.46, 586.15)	542.00 (396.66, 687.34)	11553.81 (8745.56, 14362.07)	7625.16 (6170.45, 9079.88)	9531.08 (7580.57, 11481.59)	953.40 (904.02, 1002.77)	761.39 (737.48, 785.30)	853.30 (818.26, 888.33)
Burkina Faso	271.88 (240.68, 303.08)	231.81 (200.23, 263.40)	248.69 (217.32, 280.06)	5946.36 (5437.55, 6455.17)	4792.50 (4360.18, 5224.83)	5297.57 (4834.38, 5760.75)	592.03 (582.09, 601.97)	503.62 (499.23, 508.00)	542.61 (535.81, 549.41)
Burundi	314.19 (298.13, 330.24)	300.06 (283.35, 316.78)	310.09 (294.39, 325.78)	6955.77 (6539.71, 7371.82)	5999.18 (5609.52, 6388.84)	6539.43 (6154.31, 6924.55)	659.84 (655.54, 664.14)	576.33 (572.40, 580.26)	619.93 (616.82, 623.04)
Cabo Verde	873.00 (717.19, 1028.81)	193.84 (158.60, 229.08)	450.11 (396.81, 503.40)	14614.80 (12185.90, 17043.70)	3356.63 (2929.62, 3783.64)	7833.29 (6942.53, 8724.06)	617.96 (613.38, 622.54)	514.99 (513.67, 516.31)	563.31 (562.17, 564.45)
Cambodia	370.48 (360.81, 380.15)	279.88 (271.27, 288.50)	316.98 (308.43, 325.53)	7302.41 (7060.50, 7544.32)	5162.74 (4962.61, 5362.87)	6084.76 (5878.04, 6291.48)	645.42 (638.98, 651.86)	585.20 (577.60, 592.80)	612.28 (605.14, 619.42)
Cameroon	247.73 (230.95, 264.51)	210.10 (196.99, 223.22)	227.58 (213.31, 241.85)	5192.85 (4899.74, 5485.96)	4041.02 (3827.73, 4254.31)	4599.27 (4362.95, 4835.59)	625.23 (612.67, 637.80)	546.47 (536.67, 556.26)	584.62 (573.50, 595.74)
Canada	147.59 (134.66,	94.93 (86.91,	119.87 (110.40,	2870.19 (2637.45,	1654.72 (1545.73,	2230.12 (2077.67,	719.30 (708.85,	553.00 (547.89,	633.51 (626.12,

	160.53)	102.96)	129.35)	3102.93)	1763.70)	2382.57)	729.74)	558.11)	640.90)
Central African Republic	407.70 (392.05, 423.34)	376.44 (356.17, 396.71)	398.97 (381.73, 416.20)	9546.76 (9117.19, 9976.32)	7500.36 (6937.11, 8063.62)	8536.28 (8069.76, 9002.80)	745.01 (742.30, 747.73)	699.86 (698.39, 701.33)	720.45 (718.99, 721.90)
Chad	257.38 (240.01, 274.76)	272.64 (259.70, 285.58)	263.96 (249.34, 278.59)	5556.51 (5100.54, 6012.48)	5570.64 (5238.22, 5903.07)	5539.44 (5156.97, 5921.90)	622.70 (619.81, 625.58)	560.73 (557.20, 564.25)	593.94 (590.85, 597.02)
Chile	134.18 (106.32, 162.03)	110.87 (91.73, 130.02)	125.59 (105.15, 146.02)	2481.63 (1943.90, 3019.36)	1886.10 (1625.99, 2146.20)	2197.77 (1848.26, 2547.29)	688.99 (660.08, 717.90)	521.51 (496.36, 546.66)	600.01 (572.94, 627.09)
China	262.52 (216.24, 308.79)	194.46 (162.88, 226.03)	231.09 (194.89, 267.29)	4855.16 (4147.56, 5562.75)	3407.43 (2969.81, 3845.06)	4144.43 (3603.89, 4684.97)	641.06 (619.68, 662.44)	668.89 (657.38, 680.39)	655.86 (641.80, 669.92)
Colombia	121.43 (39.74, 203.12)	98.89 (75.92, 121.85)	107.76 (62.86, 152.66)	2347.15 (709.19, 3985.10)	1814.88 (1476.90, 2152.87)	2035.37 (1168.00, 2902.75)	440.44 (430.61, 450.26)	384.99 (378.25, 391.72)	409.66 (401.46, 417.85)
Comoros	217.92 (182.96, 252.89)	251.04 (227.23, 274.84)	239.31 (211.57, 267.05)	4077.47 (2921.00, 5233.94)	4263.56 (3336.11, 5191.00)	4239.76 (3226.02, 5253.49)	636.41 (633.01, 639.81)	586.89 (580.92, 592.86)	609.14 (604.57, 613.71)
Congo	327.33 (304.30, 350.36)	334.92 (299.81, 370.04)	332.84 (307.05, 358.63)	6769.52 (6070.70, 7468.34)	6246.76 (5344.50, 7149.02)	6520.69 (5795.58, 7245.80)	699.76 (693.74, 705.78)	693.52 (687.24, 699.79)	696.49 (691.19, 701.79)
Cook Islands	272.38 (259.73, 285.03)	178.98 (169.98, 187.98)	223.83 (214.42, 233.24)	6652.68 (6366.02, 6939.34)	3601.56 (3424.52, 3778.59)	5056.33 (4852.38, 5260.28)	698.23 (695.34, 701.11)	617.20 (609.17, 625.24)	654.13 (648.71, 659.54)
Costa Rica	167.24	106.24	134.15	3422.47	1922.37	2603.38	523.22	459.16	486.98

	(129.88, 204.60)	(76.33, 136.15)	(102.64, 165.65)	(2699.58, 4145.36)	(1369.17, 2475.57)	(2008.36, 3198.39)	(519.86, 526.59)	(455.38, 462.93)	(483.59, 490.38)
Croatia	258.29 (182.97, 333.60)	177.47 (116.71, 238.22)	211.72 (149.14, 274.29)	4511.87 (3180.30, 5843.43)	2711.34 (1858.36, 3564.32)	3549.01 (2560.50, 4537.52)	782.94 (745.19, 820.70)	621.56 (583.48, 659.64)	701.16 (663.42, 738.90)
Cuba	239.88 (187.61, 292.16)	168.43 (130.61, 206.26)	200.63 (157.57, 243.69)	4685.28 (3795.81, 5574.76)	2897.22 (2303.90, 3490.54)	3715.93 (3011.76, 4420.10)	732.06 (705.08, 759.04)	638.19 (627.87, 648.52)	682.70 (665.65, 699.76)
Cyprus	159.70 (80.61, 238.79)	175.11 (111.27, 238.95)	173.97 (137.99, 209.96)	3472.06 (2665.84, 4278.27)	2318.41 (1658.76, 2978.06)	2910.28 (2539.77, 3280.80)	622.42 (585.78, 659.05)	501.99 (476.47, 527.50)	559.76 (530.56, 588.96)
Czechia	256.54 (204.00, 309.08)	178.69 (129.70, 227.68)	214.92 (168.22, 261.62)	4522.25 (3489.42, 5555.09)	2685.52 (1915.05, 3455.99)	3589.63 (2804.68, 4374.59)	879.13 (861.45, 896.81)	717.46 (710.24, 724.68)	798.34 (786.15, 810.54)
Côte d'Ivoire	226.13 (204.54, 247.73)	204.76 (186.89, 222.63)	214.32 (195.38, 233.26)	4618.98 (4121.04, 5116.91)	3877.91 (3505.14, 4250.68)	4249.65 (3817.83, 4681.48)	678.91 (669.38, 688.43)	593.13 (587.75, 598.51)	638.58 (631.30, 645.86)
Democratic People's Republic of Korea	379.84 (376.07, 383.60)	252.90 (249.74, 256.07)	304.33 (301.09, 307.57)	7921.44 (7777.83, 8065.05)	5079.42 (4974.56, 5184.28)	6386.93 (6269.02, 6504.84)	734.07 (718.97, 749.17)	698.67 (693.38, 703.96)	714.31 (704.86, 723.75)
Democratic Republic of the Congo	309.78 (288.13, 331.43)	311.27 (287.79, 334.74)	315.21 (293.03, 337.38)	6440.42 (5881.06, 6999.78)	5776.00 (5283.04, 6268.95)	5734.97 (5514.40, 5955.54)	652.21 (646.15, 658.27)	622.26 (617.70, 626.83)	636.36 (633.03, 639.69)
Denmark	137.60 (113.85, 161.36)	84.91 (60.60, 109.21)	109.55 (84.78, 134.33)	2467.89 (2015.85, 2919.94)	1405.11 (1061.95, 1748.28)	1921.54 (1532.12, 2310.97)	690.94 (667.65, 714.22)	530.55 (507.86, 553.23)	609.30 (586.41, 632.19)

Djibouti	298.61 (280.28, 316.93)	286.97 (271.86, 302.08)	295.64 (279.29, 312.00)	6250.70 (5713.36, 6788.04)	5281.61 (4880.92, 5682.30)	5827.76 (5360.59, 6294.94)	661.78 (656.14, 667.42)	578.11 (574.46, 581.76)	622.42 (617.75, 627.09)
Dominica	269.11 (252.93, 285.29)	240.92 (208.08, 273.76)	257.27 (237.03, 277.52)	5477.43 (5151.27, 5803.60)	4270.58 (3735.85, 4805.32)	4866.04 (4503.90, 5228.18)	780.26 (766.17, 794.34)	694.77 (691.09, 698.45)	738.61 (732.93, 744.28)
Dominican Republic	245.86 (200.88, 290.83)	187.20 (133.56, 240.84)	213.04 (169.12, 256.96)	5385.02 (4517.43, 6252.62)	3357.81 (2536.41, 4179.21)	4326.78 (3630.41, 5023.15)	822.62 (797.40, 847.84)	722.01 (714.59, 729.43)	770.03 (754.02, 786.05)
Ecuador	180.07 (137.17, 222.96)	140.28 (97.32, 183.23)	159.29 (120.77, 197.82)	3570.63 (2723.56, 4417.69)	2467.25 (1636.10, 3298.40)	3010.73 (2261.55, 3759.92)	412.54 (398.01, 427.07)	360.28 (349.71, 370.85)	384.93 (372.49, 397.36)
Egypt	417.61 (262.15, 573.07)	372.11 (245.97, 498.25)	372.36 (236.01, 508.72)	8739.21 (6547.79, 10930.63)	7791.87 (5622.11, 9961.62)	7789.69 (5580.25, 9999.12)	1201.07 (1162.95, 1239.18)	1192.65 (1154.89, 1230.41)	1179.94 (1144.33, 1215.56)
El Salvador	156.90 (76.58, 237.21)	130.86 (106.08, 155.64)	139.88 (94.99, 184.77)	3133.01 (1385.65, 4880.37)	2385.26 (1905.00, 2865.51)	2656.37 (1646.05, 3666.69)	511.01 (505.94, 516.08)	452.65 (451.30, 453.99)	475.55 (472.67, 478.43)
Equatorial Guinea	284.15 (250.91, 317.39)	275.76 (240.27, 311.24)	281.13 (248.34, 313.91)	5630.27 (4703.02, 6557.52)	4819.66 (3967.21, 5672.11)	5198.67 (4355.26, 6042.09)	639.93 (631.86, 648.00)	610.68 (606.07, 615.28)	623.74 (618.01, 629.47)
Eritrea	299.45 (266.54, 332.36)	309.64 (295.84, 323.44)	312.09 (290.47, 333.71)	6465.90 (5457.62, 7474.19)	5929.16 (5594.44, 6263.88)	6274.38 (5656.40, 6892.36)	645.32 (636.19, 654.45)	567.78 (560.92, 574.65)	599.71 (592.04, 607.39)
Estonia	255.04 (31.97,	226.75 (114.32,	246.02 (92.17,	4584.27 (-896.06,	3423.32 (1369.70,	4094.63 (631.31,	1273.36 (1242.54,	954.01 (926.64,	1117.22 (1086.18,

	478.11)	339.18)	399.88)	10064.60)	5476.93)	7557.96)	1304.18)	981.38)	1148.26)
	356.99	250.83	295.03	7516.74	4404.99	5741.01	726.73	645.46	678.41
Eswatini	(324.83,	(215.74,	(264.34,	(6660.65,	(3637.61,	(5037.41,	(719.21,	(632.72,	(667.51,
	389.15)	285.93)	325.72)	8372.82)	5172.36)	6444.61)	734.25)	658.20)	689.31)
	222.87	240.58	231.77	4559.56	4370.67	4464.58	554.84	470.62	512.03
Ethiopia	(212.47,	(222.42,	(218.33,	(4305.78,	(3983.73,	(4164.64,	(545.65,	(469.60,	(507.12,
	233.26)	258.74)	245.21)	4813.33)	4757.61)	4764.52)	564.04)	471.63)	516.94)
	532.30	277.99	386.10	11943.07	6119.94	8817.11	719.33	670.91	693.07
Fiji	(430.01,	(216.36,	(310.98,	(9831.97,	(4775.33,	(7040.66,	(708.25,	(660.24,	(682.70,
	634.59)	339.61)	461.21)	14054.16)	7464.56)	10593.55)	730.42)	681.58)	703.44)
	207.91	130.60	165.71	3730.59	1935.56	2761.40	761.10	513.93	637.94
Finland	(184.38,	(110.39,	(146.88,	(3289.48,	(1666.35,	(2472.93,	(743.16,	(507.69,	(627.31,
	231.45)	150.81)	184.53)	4171.70)	2204.77)	3049.87)	779.05)	520.17)	648.57)
	109.13	74.64	90.77	1996.13	1177.81	1566.53	595.38	429.71	508.95
France	(94.97,	(63.20,	(78.44,	(1771.48,	(1035.87,	(1391.36,	(579.79,	(420.37,	(497.14,
	123.28)	86.08)	103.11)	2220.79)	1319.74)	1741.70)	610.97)	439.05)	520.77)
	297.08	242.72	270.19	5944.44	4259.66	5057.90	675.15	615.14	642.50
Gabon	(270.13,	(214.57,	(244.71,	(5207.47,	(3662.58,	(4461.58,	(673.78,	(610.69,	(639.85,
	324.04)	270.86)	295.67)	6681.41)	4856.73)	5654.22)	676.53)	619.59)	645.15)
	353.70	339.56	347.87	7320.49	6572.06	6948.88	699.13	625.56	660.14
Gambia	(300.39,	(284.10,	(293.42,	(5748.90,	(5056.35,	(5408.69,	(694.58,	(622.27,	(656.41,
	407.01)	395.01)	402.33)	8892.08)	8087.77)	8489.08)	703.68)	628.86)	663.87)
	312.01	165.80	226.60	6370.14	2882.20	4440.76	815.62	606.90	702.51
Georgia	(131.27,	(58.91,	(92.77,	(2712.57,	(1102.11,	(1888.18,	(647.02,	(518.32,	(582.72,
	492.76)	272.70)	360.44)	10027.70)	4662.29)	6993.34)	984.21)	695.48)	822.30)
Germany	154.16	116.52	136.95	2741.94	1770.14	2248.59	552.52	384.17	464.26

	(126.61, 181.72)	(95.20, 137.85)	(113.87, 160.03)	(2361.25, 3122.63)	(1520.71, 2019.57)	(1944.85, 2552.34)	(514.79, 590.26)	(355.43, 412.90)	(431.14, 497.37)
Ghana	253.50 (238.90, 268.10)	306.64 (289.54, 323.74)	288.32 (273.60, 303.03)	4948.44 (4615.91, 5280.97)	5459.43 (5083.14, 5835.72)	5289.86 (4968.97, 5610.74)	668.27 (662.70, 673.84)	627.16 (613.09, 641.23)	644.65 (636.30, 653.01)
Greece	241.62 (214.65, 268.59)	248.79 (220.03, 277.55)	247.99 (221.11, 274.86)	4424.36 (3986.84, 4861.88)	3227.27 (2861.77, 3592.76)	3841.04 (3463.76, 4218.33)	634.36 (607.93, 660.79)	482.09 (457.69, 506.48)	555.32 (530.05, 580.58)
Greenland	247.50 (233.52, 261.48)	138.22 (25.47, 250.97)	189.43 (127.24, 251.62)	5146.92 (4842.00, 5451.84)	2622.73 (906.23, 4339.23)	3837.95 (2880.71, 4795.19)	810.09 (795.73, 824.46)	588.52 (570.94, 606.10)	705.43 (689.92, 720.94)
Grenada	209.93 (39.54, 380.32)	214.23 (152.50, 275.96)	223.41 (165.96, 280.87)	4492.61 (2294.80, 6690.42)	3896.94 (2899.56, 4894.32)	4321.23 (3414.35, 5228.10)	839.64 (835.43, 843.85)	731.34 (722.66, 740.01)	790.28 (787.09, 793.48)
Guam	275.85 (189.79, 361.91)	221.44 (117.48, 325.41)	249.61 (197.87, 301.35)	6929.20 (5491.71, 8366.69)	4962.52 (3642.63, 6282.42)	5977.15 (5226.24, 6728.07)	669.99 (667.64, 672.34)	641.67 (634.05, 649.29)	655.69 (651.56, 659.81)
Guatemala	175.23 (121.85, 228.61)	186.45 (123.46, 249.44)	180.46 (123.80, 237.13)	3067.08 (1945.95, 4188.21)	3225.63 (1973.05, 4478.21)	3143.51 (1981.22, 4305.80)	497.61 (492.74, 502.47)	454.34 (447.95, 460.72)	471.97 (467.03, 476.90)
Guinea	225.06 (214.11, 236.00)	257.64 (247.20, 268.08)	241.51 (231.23, 251.79)	4682.59 (4407.56, 4957.61)	5031.27 (4800.62, 5261.91)	4833.36 (4590.01, 5076.70)	611.43 (607.57, 615.28)	550.38 (544.22, 556.53)	580.79 (576.14, 585.43)
Guinea-Bissau	335.78 (323.37, 348.19)	296.59 (286.32, 306.87)	315.55 (304.84, 326.26)	7173.42 (6851.98, 7494.86)	5731.08 (5457.95, 6004.20)	6394.57 (6115.41, 6673.73)	714.51 (707.26, 721.76)	635.88 (631.84, 639.92)	671.49 (666.01, 676.96)

Guyana	387.01 (247.23, 526.79)	334.30 (221.87, 446.73)	365.30 (247.55, 483.04)	8855.94 (6589.41, 11122.47)	6529.31 (4337.00, 8721.62)	7666.66 (5514.92, 9818.40)	915.67 (903.71, 927.62)	804.32 (794.31, 814.32)	856.64 (853.29, 859.99)
Haiti	393.12 (375.90, 410.33)	427.79 (400.18, 455.40)	411.56 (390.75, 432.37)	8367.37 (7902.99, 8831.74)	8536.71 (7817.22, 9256.20)	8433.69 (7869.19, 8998.20)	843.68 (818.92, 868.44)	820.06 (816.24, 823.88)	829.98 (819.28, 840.68)
Honduras	338.72 (281.32, 396.12)	255.70 (158.22, 353.19)	293.32 (233.55, 353.09)	6176.49 (5323.26, 7029.72)	4672.01 (3157.95, 6186.08)	5343.96 (4397.83, 6290.08)	535.91 (532.84, 538.97)	492.28 (487.22, 497.34)	511.41 (509.00, 513.83)
Hungary	327.86 (241.32, 414.39)	214.67 (178.42, 250.93)	269.25 (211.90, 326.60)	6322.31 (4007.98, 8636.65)	3656.01 (2793.72, 4518.30)	5053.24 (3477.47, 6629.00)	921.95 (880.46, 963.44)	751.84 (734.87, 768.80)	833.91 (807.32, 860.50)
Iceland	149.68 (111.50, 187.86)	62.51 (56.38, 68.65)	102.07 (84.81, 119.34)	2561.53 (1992.89, 3130.18)	961.20 (885.85, 1036.56)	1721.25 (1466.45, 1976.05)	550.55 (521.91, 579.19)	333.77 (315.24, 352.31)	442.67 (419.57, 465.76)
India	257.03 (187.55, 326.51)	205.76 (129.24, 282.29)	235.15 (159.02, 311.27)	6032.46 (4646.19, 7418.72)	4150.49 (2942.37, 5358.61)	5113.47 (3874.34, 6352.61)	869.62 (847.39, 891.85)	658.24 (652.87, 663.62)	760.17 (750.73, 769.62)
Indonesia	384.59 (377.15, 392.03)	307.81 (300.31, 315.31)	346.32 (340.30, 352.34)	7749.88 (7532.20, 7967.56)	5720.52 (5540.00, 5901.05)	6743.66 (6573.50, 6913.83)	614.93 (599.97, 629.89)	634.48 (618.11, 650.84)	626.33 (610.57, 642.10)
Iran (Islamic Republic of)	243.61 (230.19, 257.03)	216.71 (204.29, 229.13)	229.73 (217.86, 241.60)	4819.73 (4594.27, 5045.19)	3681.95 (3535.62, 3828.27)	4264.07 (4101.18, 4426.95)	1504.39 (1437.03, 1571.76)	1126.40 (1082.15, 1170.65)	1316.35 (1260.93, 1371.77)
Iraq	603.44 (558.24,	383.30 (344.11,	487.07 (445.73,	11259.09 (10544.22,	6772.81 (6222.72,	8937.37 (8316.28,	1191.69 (1169.19,	1030.84 (1016.35,	1111.94 (1093.29,

	648.63)	422.49)	528.41)	11973.95)	7322.91)	9558.47)	1214.19)	1045.33)	1130.58)
Ireland	140.55 (109.96, 171.14)	95.46 (74.77, 116.16)	118.50 (94.48, 142.53)	2522.45 (1987.55, 3057.35)	1488.25 (1192.56, 1783.94)	2012.04 (1642.76, 2381.32)	655.14 (625.23, 685.04)	481.48 (460.46, 502.50)	566.17 (541.65, 590.69)
Israel	125.76 (97.95, 153.57)	95.19 (74.70, 115.69)	110.64 (87.69, 133.59)	2308.01 (1792.50, 2823.52)	1457.93 (1168.65, 1747.20)	1876.09 (1499.80, 2252.37)	590.53 (560.86, 620.20)	438.22 (420.18, 456.26)	512.48 (489.24, 535.72)
Italy	133.01 (118.48, 147.54)	103.18 (87.68, 118.67)	118.61 (103.03, 134.20)	2341.71 (2103.22, 2580.20)	1549.49 (1364.04, 1734.94)	1941.68 (1729.64, 2153.71)	852.67 (840.41, 864.93)	721.59 (696.81, 746.37)	784.43 (767.38, 801.48)
Jamaica	69.57 (-89.14, 228.29)	171.06 (103.90, 238.22)	126.09 (22.21, 229.97)	1503.83 (-1678.13, 4685.80)	3021.11 (1870.29, 4171.92)	2277.68 (249.32, 4306.05)	860.58 (826.48, 894.68)	759.66 (749.30, 770.02)	808.85 (787.11, 830.59)
Japan	87.05 (74.08, 100.01)	65.93 (55.72, 76.15)	78.50 (66.58, 90.41)	1854.50 (1638.24, 2070.76)	1279.72 (1138.62, 1420.83)	1592.74 (1411.35, 1774.13)	576.89 (563.43, 590.34)	626.90 (618.73, 635.06)	602.19 (592.90, 611.49)
Jordan	275.65 (215.99, 335.31)	321.22 (162.65, 479.80)	281.08 (199.13, 363.02)	5155.23 (4305.70, 6004.75)	5200.06 (3132.95, 7267.17)	5125.50 (3932.69, 6318.31)	1167.15 (1131.33, 1202.97)	1017.21 (976.66, 1057.75)	1101.71 (1063.56, 1139.86)
Kazakhstan	411.29 (249.17, 573.41)	300.84 (211.35, 390.34)	350.90 (238.47, 463.34)	8877.26 (5381.22, 12373.31)	5394.52 (3743.97, 7045.08)	7025.52 (4634.52, 9416.52)	1057.62 (980.03, 1135.22)	823.92 (777.98, 869.85)	929.49 (870.59, 988.39)
Kenya	254.22 (245.30, 263.14)	226.96 (219.88, 234.04)	242.23 (234.85, 249.61)	5220.46 (4992.01, 5448.91)	4198.33 (4060.89, 4335.77)	4718.69 (4549.68, 4887.71)	644.05 (640.30, 647.80)	545.16 (544.08, 546.23)	590.62 (588.54, 592.70)
Kiribati	665.20	431.15	533.65	17990.67	9901.41	13583.75	959.67	744.29	835.38

	(637.46, 692.95)	(417.83, 444.46)	(514.25, 553.05)	(17147.20, 18834.15)	(9567.09, 10235.74)	(13030.69, 14136.81)	(942.82, 976.52)	(742.09, 746.48)	(828.40, 842.36)
Kuwait	234.50 (89.88, 379.11)	90.68 (22.40, 158.96)	173.64 (64.03, 283.24)	4716.32 (1459.70, 7972.94)	1739.76 (578.33, 2901.18)	3416.49 (992.82, 5840.16)	1119.32 (1091.12, 1147.52)	879.51 (868.02, 891.01)	1009.35 (985.99, 1032.71)
Kyrgyzstan	205.96 (-3.27, 415.19)	245.10 (102.33, 387.86)	254.34 (93.94, 414.75)	6853.24 (2982.69, 10723.80)	4611.65 (2375.97, 6847.33)	5725.15 (2785.41, 8664.88)	1136.26 (1115.41, 1157.10)	852.80 (830.42, 875.17)	977.40 (955.83, 998.97)
Lao People's Democratic Republic	427.89 (415.01, 440.77)	344.35 (333.78, 354.91)	385.53 (374.29, 396.78)	8606.89 (8274.87, 8938.92)	6302.51 (6044.09, 6560.93)	7438.22 (7154.99, 7721.46)	679.97 (668.64, 691.30)	640.62 (628.25, 652.98)	660.45 (648.64, 672.27)
Latvia	302.27 (-57.55, 662.08)	262.67 (120.06, 405.29)	291.38 (66.64, 516.13)	6194.85 (-2943.74, 15333.45)	4365.87 (1475.23, 7256.52)	5393.73 (-54.05, 10841.52)	1067.76 (1022.02, 1113.50)	853.87 (839.15, 868.59)	961.33 (940.53, 982.13)
Lebanon	333.87 (318.54, 349.20)	212.32 (202.93, 221.70)	261.75 (250.77, 272.74)	6869.34 (6523.52, 7215.16)	3901.16 (3712.73, 4089.59)	5179.80 (4944.07, 5415.53)	1059.46 (1032.79, 1086.12)	926.92 (912.95, 940.90)	982.88 (972.12, 993.64)
Lesotho	387.72 (369.22, 406.23)	279.27 (224.61, 333.93)	325.84 (289.71, 361.97)	8443.95 (7976.31, 8911.58)	4892.55 (3751.13, 6033.96)	6421.68 (5670.35, 7173.01)	714.88 (711.09, 718.67)	643.72 (640.22, 647.22)	674.05 (670.38, 677.72)
Liberia	227.80 (207.18, 248.43)	242.18 (224.48, 259.89)	234.91 (216.88, 252.94)	4780.72 (4269.29, 5292.15)	4806.88 (4452.13, 5161.63)	4802.35 (4395.88, 5208.82)	649.84 (647.23, 652.44)	594.62 (590.67, 598.58)	623.22 (620.06, 626.37)
Libya	282.51 (204.55, 360.46)	231.57 (179.03, 284.10)	255.61 (198.08, 313.15)	6130.57 (4752.14, 7509.00)	4817.88 (3735.14, 5900.62)	5449.04 (4336.97, 6561.12)	1123.72 (1091.46, 1155.97)	986.02 (969.03, 1003.01)	1055.53 (1031.34, 1079.72)

Lithuania	194.01 (-0.13, 388.15)	199.87 (109.46, 290.29)	203.65 (73.83, 333.47)	3169.56 (-1706.41, 8045.54)	2944.00 (1126.71, 4761.30)	3158.16 (78.02, 6238.30)	973.99 (947.19, 1000.80)	853.00 (844.10, 861.90)	920.53 (908.88, 932.17)
Luxembourg	161.58 (140.62, 182.54)	97.81 (85.91, 109.72)	128.26 (116.93, 139.58)	2781.48 (2410.98, 3151.98)	1559.23 (1373.77, 1744.69)	2161.68 (1955.33, 2368.02)	532.69 (521.63, 543.75)	414.32 (411.23, 417.41)	472.37 (467.36, 477.38)
Madagascar	384.39 (324.76, 444.02)	400.18 (347.62, 452.75)	395.26 (339.03, 451.49)	8133.31 (7430.50, 8836.12)	7990.41 (7280.72, 8700.10)	8100.79 (7396.03, 8805.56)	710.50 (692.15, 728.85)	661.76 (651.84, 671.68)	684.70 (670.80, 698.60)
Malawi	282.18 (261.54, 302.83)	229.81 (214.67, 244.96)	255.92 (239.24, 272.59)	6067.96 (5509.81, 6626.11)	4343.65 (3995.67, 4691.63)	5173.32 (4748.93, 5597.71)	683.40 (678.31, 688.49)	583.81 (580.68, 586.94)	626.61 (623.87, 629.34)
Malaysia	286.23 (168.08, 404.38)	186.76 (129.78, 243.74)	239.34 (121.33, 357.36)	6813.40 (4842.93, 8783.87)	4047.61 (3241.34, 4853.88)	5538.10 (4458.02, 6618.19)	648.67 (636.00, 661.33)	563.16 (549.55, 576.77)	606.86 (594.09, 619.63)
Maldives	234.81 (201.84, 267.78)	149.50 (59.70, 239.29)	197.41 (150.48, 244.33)	4549.28 (3841.58, 5256.97)	2659.72 (1226.20, 4093.23)	3843.62 (2995.24, 4692.00)	562.32 (556.66, 567.99)	479.97 (475.70, 484.25)	527.87 (522.97, 532.76)
Mali	214.42 (204.42, 224.41)	274.57 (261.36, 287.77)	243.03 (232.31, 253.76)	4048.85 (3874.52, 4223.18)	5388.85 (5113.93, 5663.76)	4690.77 (4495.78, 4885.77)	602.10 (599.53, 604.67)	564.13 (555.36, 572.89)	584.18 (579.82, 588.54)
Malta	177.47 (131.21, 223.74)	96.37 (74.21, 118.53)	135.20 (115.52, 154.87)	3155.84 (2405.97, 3905.70)	1500.53 (1206.38, 1794.67)	2276.19 (1951.12, 2601.26)	426.40 (399.56, 453.25)	382.16 (371.46, 392.86)	406.85 (389.03, 424.67)
Marshall Islands	594.46 (432.90,	457.37 (448.55,	521.88 (448.02,	14688.81 (11589.01,	9849.90 (9648.69,	12228.17 (10773.60,	782.25 (774.51,	712.17 (706.78,	747.63 (741.21,

	756.02)	466.19)	595.73)	17788.61)	10051.11)	13682.73)	789.99)	717.57)	754.05)
Mauritania	192.77 (171.20, 214.35)	236.26 (214.13, 258.40)	213.35 (191.88, 234.83)	3597.16 (3093.08, 4101.24)	4276.80 (3730.01, 4823.59)	3923.53 (3403.93, 4443.13)	624.22 (620.78, 627.66)	581.67 (574.76, 588.58)	603.11 (600.21, 606.01)
Mauritius	242.43 (112.87, 372.00)	189.79 (142.53, 237.06)	212.54 (153.15, 271.94)	4593.44 (1949.08, 7237.80)	3617.33 (2453.71, 4780.94)	4070.72 (2714.52, 5426.91)	596.38 (587.55, 605.21)	526.72 (520.20, 533.23)	559.77 (552.32, 567.23)
Mexico	166.46 (134.42, 198.49)	113.35 (89.77, 136.93)	136.61 (109.18, 164.05)	3805.20 (3412.88, 4197.53)	2040.70 (1648.63, 2432.76)	2760.30 (2310.01, 3210.60)	489.40 (484.87, 493.93)	482.27 (477.06, 487.48)	485.69 (481.58, 489.80)
Micronesia (Federated States of)	634.97 (624.66, 645.27)	482.24 (472.82, 491.66)	560.59 (551.10, 570.09)	15730.75 (15463.21, 15998.29)	10133.54 (9934.42, 10332.67)	12964.83 (12742.03, 13187.62)	743.79 (736.10, 751.48)	665.44 (661.77, 669.11)	702.92 (697.40, 708.43)
Monaco	132.22 (120.76, 143.68)	99.68 (91.57, 107.78)	115.28 (105.80, 124.76)	2422.44 (2239.62, 2605.26)	1552.92 (1432.01, 1673.83)	1972.71 (1825.73, 2119.69)	659.84 (654.96, 664.73)	494.99 (491.31, 498.67)	575.25 (570.95, 579.55)
Mongolia	942.52 (774.88, 1110.15)	396.18 (277.40, 514.97)	597.69 (477.19, 718.19)	17154.61 (14701.48, 19607.75)	7729.33 (6123.97, 9334.69)	11662.18 (9838.85, 13485.50)	1376.62 (1361.39, 1391.84)	1021.46 (1008.38, 1034.55)	1177.69 (1164.68, 1190.71)
Montenegro	382.16 (286.01, 478.31)	152.86 (81.94, 223.78)	238.02 (190.53, 285.51)	7480.51 (5910.84, 9050.18)	3247.38 (2290.60, 4204.16)	5059.13 (4220.46, 5897.79)	913.37 (908.69, 918.05)	772.98 (765.67, 780.29)	843.87 (840.90, 846.83)
Morocco	479.40 (305.00, 653.80)	357.12 (343.74, 370.50)	410.46 (317.79, 503.13)	9157.03 (7086.95, 11227.11)	6527.72 (6217.70, 6837.73)	7760.61 (6646.10, 8875.12)	1155.44 (1139.54, 1171.34)	981.26 (962.98, 999.55)	1065.32 (1058.78, 1071.86)
Mozambique	378.00	271.26	320.31	8354.94	5164.66	6626.45	811.71	654.76	721.58

	(358.94, 397.06)	(258.01, 284.52)	(304.47, 336.14)	(7957.65, 8752.23)	(4935.53, 5393.78)	(6330.99, 6921.91)	(802.46, 820.97)	(653.23, 656.29)	(717.21, 725.95)
Myanmar	374.26 (360.34, 388.17)	299.23 (291.20, 307.25)	333.88 (325.56, 342.20)	7869.71 (7486.69, 8252.73)	5216.43 (5011.32, 5421.55)	6427.80 (6191.17, 6664.42)	688.99 (683.74, 694.25)	525.56 (515.77, 535.35)	594.24 (586.31, 602.17)
Namibia	352.38 (331.40, 373.36)	258.63 (234.77, 282.48)	299.68 (277.82, 321.54)	6944.64 (6393.54, 7495.74)	4300.50 (3779.70, 4821.29)	5475.98 (4971.78, 5980.17)	672.81 (668.40, 677.22)	598.88 (593.91, 603.85)	629.73 (624.96, 634.50)
Nauru	642.95 (617.63, 668.26)	481.50 (456.58, 506.42)	548.82 (525.72, 571.91)	16185.46 (15442.44, 16928.47)	10347.62 (9674.87, 11020.36)	13047.98 (12409.61, 13686.34)	757.57 (746.17, 768.96)	698.12 (695.35, 700.90)	723.44 (716.73, 730.16)
Nepal	334.44 (319.34, 349.53)	192.54 (187.09, 197.99)	256.13 (247.44, 264.82)	6562.14 (6218.97, 6905.31)	3643.32 (3507.74, 3778.91)	4953.53 (4754.24, 5152.82)	805.31 (802.27, 808.35)	543.44 (535.22, 551.67)	660.91 (655.91, 665.90)
Netherlands	130.92 (103.75, 158.10)	94.06 (80.35, 107.77)	112.13 (93.34, 130.91)	2336.92 (1858.73, 2815.11)	1523.52 (1300.92, 1746.12)	1925.26 (1598.86, 2251.66)	820.10 (802.65, 837.55)	499.39 (491.57, 507.21)	656.90 (645.17, 668.62)
New Zealand	177.75 (146.11, 209.39)	113.36 (90.57, 136.15)	145.04 (120.99, 169.08)	3169.50 (2684.44, 3654.56)	1784.17 (1422.70, 2145.65)	2463.50 (2084.64, 2842.36)	633.57 (607.86, 659.29)	513.11 (500.92, 525.29)	569.08 (550.46, 587.71)
Nicaragua	288.20 (218.69, 357.70)	125.76 (-50.08, 301.60)	194.41 (67.97, 320.84)	4714.21 (3357.83, 6070.59)	2146.34 (-258.99, 4551.68)	3304.86 (1498.01, 5111.72)	527.60 (516.29, 538.92)	472.95 (464.50, 481.40)	495.77 (486.00, 505.54)
Niger	250.96 (227.23, 274.69)	268.90 (249.92, 287.88)	260.11 (238.79, 281.44)	5236.47 (4700.26, 5772.67)	5323.38 (4921.15, 5725.61)	5273.00 (4804.14, 5741.86)	613.11 (609.35, 616.88)	556.97 (554.53, 559.42)	583.59 (581.07, 586.11)

Nigeria	195.77 (182.85, 208.68)	208.09 (194.48, 221.70)	201.20 (188.17, 214.22)	3995.74 (3749.38, 4242.10)	3872.95 (3665.54, 4080.37)	3904.39 (3684.97, 4123.80)	611.74 (605.84, 617.64)	551.15 (548.39, 553.91)	575.90 (572.57, 579.22)
Niue	433.25 (414.80, 451.69)	263.40 (241.55, 285.25)	342.13 (325.96, 358.29)	10081.22 (9499.38, 10663.07)	5573.15 (5044.98, 6101.32)	7765.79 (7325.44, 8206.14)	687.60 (685.64, 689.55)	620.03 (618.70, 621.36)	653.06 (651.86, 654.27)
North Macedonia	471.62 (328.66, 614.58)	454.87 (390.36, 519.38)	504.55 (428.38, 580.72)	8269.16 (5888.87, 10649.45)	6911.26 (5937.26, 7885.27)	7977.15 (6763.50, 9190.79)	846.67 (810.17, 883.17)	732.45 (701.52, 763.39)	792.90 (759.80, 825.99)
Northern Mariana Islands	266.01 (248.08, 283.93)	196.00 (174.43, 217.58)	233.87 (215.93, 251.80)	6307.23 (5948.41, 6666.06)	4062.77 (3641.68, 4483.85)	5338.02 (4968.21, 5707.84)	687.82 (684.00, 691.64)	621.64 (620.19, 623.08)	656.48 (650.77, 662.20)
Norway	118.81 (93.53, 144.09)	92.56 (76.34, 108.78)	108.80 (90.87, 126.72)	2198.88 (1804.20, 2593.56)	1424.36 (1221.76, 1626.97)	1851.98 (1595.66, 2108.31)	651.43 (634.25, 668.62)	409.33 (384.95, 433.71)	531.91 (517.08, 546.75)
Oman	-106.83 (-229.43, 15.78)	132.38 (-17.37, 282.14)	-15.59 (-123.00, 91.83)	227.17 (-1239.22, 1693.57)	3925.03 (2135.87, 5714.18)	1797.59 (416.00, 3179.19)	1197.47 (1166.31, 1228.63)	984.80 (964.71, 1004.88)	1094.27 (1073.58, 1114.96)
Pakistan	322.08 (309.89, 334.26)	297.09 (281.31, 312.87)	309.86 (296.85, 322.87)	7133.87 (6826.29, 7441.46)	6044.65 (5681.89, 6407.42)	6592.95 (6285.96, 6899.95)	956.91 (902.67, 1011.16)	779.62 (757.37, 801.87)	870.71 (831.98, 909.44)
Palau	431.37 (413.65, 449.08)	252.38 (241.49, 263.28)	344.82 (335.10, 354.54)	10961.09 (10619.40, 11302.78)	5402.47 (5115.47, 5689.46)	8454.99 (8240.10, 8669.89)	719.94 (718.90, 720.98)	620.26 (612.49, 628.03)	671.29 (667.32, 675.27)
Palestine	398.62 (323.02,	288.52 (240.14,	330.39 (277.71,	7384.16 (6253.01,	5197.60 (4377.26,	6192.94 (5328.55,	1132.52 (1123.23,	975.79 (958.34,	1056.11 (1042.03,

	474.21)	336.90)	383.07)	8515.31)	6017.95)	7057.33)	1141.82)	993.23)	1070.20)
Panama	112.36 (70.96, 153.77)	91.90 (51.87, 131.93)	102.58 (70.35, 134.80)	2323.53 (1596.56, 3050.50)	1770.40 (1024.34, 2516.47)	2043.15 (1458.84, 2627.46)	523.74 (520.99, 526.48)	455.45 (453.47, 457.43)	488.31 (486.18, 490.45)
Papua New Guinea	433.80 (418.45, 449.16)	311.54 (300.39, 322.70)	375.21 (361.67, 388.75)	10735.07 (10216.70, 11253.45)	7097.51 (6731.20, 7463.82)	8989.38 (8542.82, 9435.93)	710.92 (701.64, 720.19)	651.51 (648.26, 654.77)	682.74 (677.01, 688.47)
Paraguay	232.16 (173.02, 291.29)	147.88 (100.24, 195.52)	185.93 (134.46, 237.40)	4833.47 (3709.45, 5957.49)	2914.62 (2086.72, 3742.52)	3835.14 (2913.03, 4757.24)	506.90 (498.46, 515.35)	427.30 (423.02, 431.58)	465.24 (459.21, 471.28)
Peru	99.63 (46.78, 152.48)	75.11 (30.78, 119.44)	88.80 (42.64, 134.96)	2130.92 (1062.83, 3199.01)	1453.77 (612.51, 2295.03)	1814.87 (926.29, 2703.45)	331.77 (320.21, 343.34)	302.62 (295.53, 309.70)	316.41 (307.18, 325.65)
Philippines	272.87 (236.53, 309.21)	228.21 (183.83, 272.60)	253.35 (214.70, 291.99)	6113.67 (5334.84, 6892.50)	4186.30 (3495.07, 4877.54)	5184.41 (4466.29, 5902.52)	715.00 (709.39, 720.61)	667.07 (659.81, 674.33)	691.29 (685.24, 697.34)
Poland	266.40 (201.86, 330.95)	186.14 (151.56, 220.72)	224.24 (175.65, 272.84)	5114.38 (3526.68, 6702.09)	2943.40 (2351.72, 3535.08)	3995.98 (2937.95, 5054.02)	535.78 (482.78, 588.78)	479.99 (446.36, 513.61)	507.03 (465.32, 548.74)
Portugal	149.13 (122.20, 176.05)	114.44 (93.29, 135.60)	130.52 (108.00, 153.03)	2672.31 (2232.26, 3112.35)	1735.54 (1436.77, 2034.31)	2174.10 (1824.28, 2523.92)	497.65 (474.41, 520.88)	395.95 (378.73, 413.17)	443.30 (423.23, 463.37)
Puerto Rico	124.36 (96.91, 151.81)	104.13 (76.35, 131.91)	113.78 (86.38, 141.18)	2823.73 (2126.75, 3520.71)	1839.29 (1378.02, 2300.55)	2284.02 (1756.63, 2811.41)	743.06 (704.08, 782.04)	627.03 (613.99, 640.06)	680.46 (655.81, 705.10)
Qatar	265.35	271.68	247.24	3087.26	4819.77	3332.52	956.90	812.70	925.60

	(122.40, 408.30)	(62.17, 481.18)	(214.25, 280.23)	(901.19, 5273.34)	(1932.83, 7706.71)	(1495.03, 5170.01)	(949.96, 963.85)	(793.54, 831.86)	(916.76, 934.44)
Republic of Korea	116.68 (72.30, 161.06)	108.27 (90.99, 125.56)	116.05 (94.08, 138.01)	1960.13 (1414.69, 2505.57)	1656.10 (1485.61, 1826.60)	1878.03 (1596.04, 2160.01)	442.69 (416.55, 468.83)	420.73 (399.54, 441.91)	435.07 (411.61, 458.53)
Republic of Moldova	133.25 (-137.64, 404.14)	-7.16 (-212.07, 197.75)	24.00 (-200.03, 248.03)	2527.62 (-3466.60, 8521.83)	546.10 (-3334.67, 4426.88)	1052.33 (-3742.22, 5846.88)	996.93 (939.27, 1054.60)	790.67 (752.70, 828.64)	887.26 (842.02, 932.50)
Romania	373.20 (209.76, 536.63)	313.90 (234.18, 393.62)	342.45 (231.56, 453.35)	6256.27 (3072.64, 9439.91)	4696.82 (3444.88, 5948.76)	5455.92 (3423.43, 7488.41)	889.79 (830.31, 949.27)	754.94 (723.27, 786.61)	820.94 (777.13, 864.76)
Russian Federation	280.42 (-108.30, 669.13)	272.51 (95.93, 449.09)	288.52 (29.25, 547.80)	5774.78 (-4327.99, 15877.56)	4484.39 (816.08, 8152.70)	5299.16 (-1077.18, 11675.49)	1005.78 (947.95, 1063.60)	935.80 (926.62, 944.99)	984.21 (958.04, 1010.39)
Rwanda	275.31 (250.25, 300.37)	262.57 (240.03, 285.11)	271.78 (248.86, 294.70)	5570.11 (4911.98, 6228.24)	4766.65 (4261.49, 5271.80)	5176.81 (4634.75, 5718.88)	592.87 (584.14, 601.59)	535.26 (528.27, 542.25)	559.20 (551.79, 566.60)
Saint Kitts and Nevis	293.83 (243.79, 343.88)	179.73 (-36.82, 396.27)	271.19 (131.77, 410.60)	8061.00 (6550.99, 9571.00)	3236.41 (234.80, 6238.02)	6079.25 (4029.05, 8129.46)	770.84 (759.57, 782.12)	677.33 (668.45, 686.21)	725.34 (721.57, 729.10)
Saint Lucia	207.17 (148.35, 266.00)	223.04 (184.00, 262.09)	225.22 (193.54, 256.91)	4408.08 (3724.98, 5091.18)	3819.76 (3209.00, 4430.51)	4219.06 (3777.95, 4660.18)	840.12 (827.00, 853.23)	735.74 (726.51, 744.97)	788.28 (784.02, 792.54)
Saint Vincent and the Grenadines	268.17 (230.64, 305.69)	257.95 (199.45, 316.46)	264.63 (226.72, 302.55)	5325.56 (4758.11, 5893.01)	4503.49 (3769.33, 5237.64)	4925.56 (4389.29, 5461.83)	819.78 (801.89, 837.66)	719.19 (714.29, 724.10)	771.99 (764.96, 779.03)

Samoa	398.84 (389.13, 408.54)	355.07 (347.78, 362.35)	380.11 (372.86, 387.37)	9363.82 (9157.42, 9570.22)	7487.98 (7338.74, 7637.22)	8524.75 (8395.42, 8654.09)	761.54 (758.32, 764.75)	711.77 (710.61, 712.92)	737.44 (735.80, 739.08)
San Marino	132.93 (125.50, 140.36)	95.13 (88.06, 102.21)	112.31 (106.70, 117.92)	2433.83 (2297.41, 2570.25)	1562.75 (1458.16, 1667.35)	1965.41 (1872.31, 2058.50)	639.21 (633.09, 645.33)	483.52 (478.54, 488.51)	555.14 (549.88, 560.39)
Sao Tome and Principe	302.04 (261.13, 342.96)	294.12 (265.57, 322.67)	299.23 (274.61, 323.85)	5799.90 (5018.07, 6581.73)	5643.47 (4801.52, 6485.42)	5711.95 (4980.20, 6443.71)	691.82 (687.78, 695.86)	673.76 (666.05, 681.48)	683.28 (677.53, 689.03)
Saudi Arabia	259.25 (248.35, 270.16)	233.23 (214.99, 251.47)	249.73 (237.80, 261.66)	5648.41 (5430.72, 5866.10)	4818.33 (4519.05, 5117.62)	5324.44 (5112.50, 5536.37)	1025.32 (1002.07, 1048.56)	892.40 (874.71, 910.10)	971.42 (950.31, 992.53)
Senegal	210.86 (160.61, 261.11)	215.46 (169.45, 261.47)	213.51 (165.20, 261.83)	4180.42 (2706.38, 5654.46)	3891.36 (2625.32, 5157.41)	4033.80 (2662.72, 5404.88)	640.25 (635.01, 645.50)	581.76 (579.57, 583.95)	609.87 (607.10, 612.64)
Serbia	359.54 (229.99, 489.10)	345.98 (219.03, 472.93)	363.64 (242.30, 484.99)	6433.70 (4159.53, 8707.88)	5272.01 (3277.13, 7266.89)	5947.18 (3904.62, 7989.74)	1018.09 (972.86, 1063.32)	910.41 (866.60, 954.23)	963.96 (919.77, 1008.15)
Seychelles	303.16 (225.95, 380.37)	182.64 (159.06, 206.22)	242.29 (209.50, 275.09)	6382.08 (5227.67, 7536.50)	3477.19 (3107.12, 3847.26)	4924.27 (4377.34, 5471.21)	648.07 (641.16, 654.98)	554.08 (547.08, 561.09)	602.93 (596.27, 609.60)
Sierra Leone	258.78 (239.95, 277.62)	286.63 (271.42, 301.84)	274.32 (258.10, 290.54)	5874.05 (5395.89, 6352.21)	6081.60 (5748.42, 6414.77)	5985.63 (5593.05, 6378.20)	692.97 (689.44, 696.51)	646.62 (635.85, 657.39)	670.79 (663.55, 678.04)
Singapore	86.49 (56.71,	61.16 (35.29,	74.44 (55.01,	1783.23 (1322.94,	1188.93 (770.57,	1466.93 (1143.44,	513.02 (506.59,	453.99 (438.33,	482.38 (472.54,

	116.26)	87.03)	93.87)	2243.51)	1607.29)	1790.42)	519.44)	469.64)	492.21)
Slovakia	300.85 (226.79, 374.91)	245.21 (187.92, 302.49)	275.70 (221.97, 329.43)	5645.09 (4058.24, 7231.94)	3848.95 (2886.02, 4811.89)	4777.80 (3740.00, 5815.59)	666.27 (625.94, 706.59)	603.81 (574.36, 633.25)	639.59 (608.37, 670.80)
Slovenia	170.88 (135.30, 206.46)	120.93 (89.53, 152.34)	146.75 (123.27, 170.23)	2991.42 (2343.70, 3639.14)	1787.12 (1365.46, 2208.77)	2423.83 (2052.81, 2794.85)	964.76 (909.85, 1019.68)	651.35 (623.63, 679.07)	799.83 (759.91, 839.75)
Solomon Islands	970.87 (907.24, 1034.49)	801.45 (735.21, 867.68)	885.13 (820.75, 949.50)	24698.46 (22943.23, 26453.70)	18045.37 (16222.39, 19868.35)	21342.70 (19581.27, 23104.13)	763.65 (737.82, 789.48)	711.93 (706.45, 717.42)	737.10 (721.13, 753.07)
Somalia	345.67 (330.98, 360.37)	321.02 (305.80, 336.23)	336.14 (321.32, 350.97)	7777.89 (7416.30, 8139.48)	6487.09 (6155.32, 6818.86)	7125.64 (6777.14, 7474.15)	698.51 (696.26, 700.75)	616.27 (614.86, 617.69)	648.92 (647.31, 650.54)
South Africa	96.43 (17.47, 175.39)	64.71 (-15.76, 145.17)	77.40 (1.01, 153.78)	2262.38 (469.59, 4055.18)	1326.83 (-348.49, 3002.14)	1734.08 (88.47, 3379.68)	746.36 (730.47, 762.25)	662.67 (657.26, 668.07)	698.35 (691.10, 705.60)
South Sudan	216.66 (207.15, 226.18)	225.96 (217.43, 234.49)	220.86 (211.94, 229.78)	4564.66 (4267.54, 4861.79)	4381.27 (4139.42, 4623.12)	4460.74 (4192.05, 4729.44)	644.50 (641.57, 647.43)	558.83 (557.02, 560.64)	601.85 (599.75, 603.95)
Spain	113.99 (95.01, 132.97)	93.24 (78.12, 108.35)	104.30 (87.83, 120.76)	2139.44 (1852.90, 2425.98)	1414.67 (1236.60, 1592.74)	1773.85 (1554.72, 1992.98)	633.08 (623.13, 643.04)	464.12 (458.32, 469.91)	546.01 (538.44, 553.58)
Sri Lanka	188.05 (116.27, 259.83)	140.51 (101.20, 179.82)	163.36 (118.68, 208.04)	4156.22 (2544.26, 5768.19)	2617.52 (1913.92, 3321.12)	3337.26 (2386.90, 4287.61)	543.19 (515.98, 570.40)	472.88 (454.97, 490.78)	504.94 (483.15, 526.73)
Sudan	489.03	445.24	470.09	9464.53	8006.79	8787.31	1122.69	1018.51	1073.58

	(476.81, 501.25)	(437.54, 452.93)	(464.08, 476.09)	(9184.31, 9744.74)	(7789.34, 8224.25)	(8647.15, 8927.47)	(1106.84, 1138.53)	(1008.28, 1028.74)	(1060.76, 1086.40)
Suriname	257.85 (96.59, 419.11)	213.31 (141.68, 284.94)	237.52 (130.61, 344.44)	6423.60 (2753.23, 10093.98)	4284.30 (2912.97, 5655.63)	5354.23 (2925.65, 7782.80)	907.23 (884.99, 929.46)	774.92 (770.64, 779.20)	836.49 (824.44, 848.53)
Sweden	154.29 (136.90, 171.68)	112.72 (96.88, 128.56)	137.00 (122.14, 151.87)	2735.51 (2455.09, 3015.92)	1701.99 (1504.91, 1899.07)	2257.06 (2046.01, 2468.11)	625.02 (609.43, 640.60)	431.27 (421.31, 441.23)	527.54 (515.07, 540.01)
Switzerland	112.45 (90.68, 134.23)	83.02 (68.33, 97.71)	98.70 (82.90, 114.49)	1739.55 (1518.95, 1960.16)	1228.96 (1068.74, 1389.18)	1532.71 (1364.21, 1701.21)	446.36 (411.44, 481.28)	417.85 (412.99, 422.72)	433.00 (414.43, 451.58)
Syrian Arab Republic	500.93 (406.19, 595.66)	516.86 (411.58, 622.14)	496.59 (410.79, 582.39)	10249.54 (8349.96, 12149.12)	8432.22 (6551.65, 10312.79)	9248.92 (7486.88, 11010.96)	1165.22 (1131.48, 1198.96)	990.26 (949.47, 1031.04)	1080.77 (1047.88, 1113.66)
Taiwan (Province of China)	115.92 (91.31, 140.54)	76.50 (60.48, 92.52)	94.40 (75.80, 112.99)	2643.96 (2153.06, 3134.86)	1609.72 (1346.80, 1872.64)	2101.04 (1755.14, 2446.94)	569.02 (559.10, 578.94)	544.88 (536.75, 553.00)	554.03 (545.31, 562.76)
Tajikistan	227.41 (80.86, 373.96)	426.60 (171.72, 681.47)	394.72 (200.14, 589.30)	5798.59 (3232.92, 8364.26)	7446.64 (4451.81, 10441.47)	7146.22 (4677.74, 9614.69)	1316.25 (1301.16, 1331.34)	964.35 (959.02, 969.69)	1129.43 (1120.61, 1138.26)
Thailand	155.47 (109.15, 201.80)	95.15 (72.31, 118.00)	121.15 (88.67, 153.63)	3718.85 (2799.83, 4637.86)	1914.58 (1539.21, 2289.94)	2731.15 (2149.69, 3312.61)	543.22 (533.86, 552.58)	462.35 (455.19, 469.51)	499.80 (493.70, 505.91)
Timor-Leste	448.60 (428.31, 468.89)	364.38 (350.76, 377.99)	406.19 (389.80, 422.57)	9247.77 (8718.64, 9776.91)	6906.11 (6519.49, 7292.73)	8077.10 (7629.64, 8524.55)	688.52 (682.47, 694.56)	637.09 (635.79, 638.39)	662.81 (660.22, 665.41)

Togo	271.53 (250.45, 292.60)	244.58 (233.02, 256.13)	256.36 (242.06, 270.67)	5576.55 (5061.03, 6092.08)	4689.48 (4453.22, 4925.74)	5098.31 (4771.84, 5424.77)	681.82 (669.62, 694.02)	588.14 (584.83, 591.45)	629.01 (622.02, 636.01)
Tokelau	271.78 (268.45, 275.12)	359.95 (355.83, 364.07)	313.44 (309.09, 317.79)	6422.65 (6358.33, 6486.97)	7625.65 (7542.77, 7708.54)	6988.79 (6916.34, 7061.25)	664.93 (661.98, 667.88)	615.74 (613.52, 617.96)	642.21 (639.45, 644.96)
Tonga	265.67 (189.70, 341.64)	164.31 (141.98, 186.64)	211.53 (170.66, 252.39)	6139.23 (4722.83, 7555.62)	3457.22 (3175.34, 3739.11)	4728.53 (3981.92, 5475.14)	707.46 (695.24, 719.68)	660.73 (650.48, 670.97)	683.45 (672.28, 694.62)
Trinidad and Tobago	281.94 (214.99, 348.89)	200.48 (150.93, 250.03)	242.08 (187.61, 296.56)	5815.16 (4396.04, 7234.28)	3835.00 (2832.81, 4837.19)	4871.00 (3739.94, 6002.05)	873.83 (848.36, 899.31)	758.47 (750.77, 766.18)	814.10 (800.54, 827.67)
Tunisia	319.60 (283.58, 355.63)	230.50 (202.94, 258.07)	276.27 (253.96, 298.57)	6035.84 (5552.51, 6519.17)	3975.75 (3585.28, 4366.22)	4983.21 (4641.29, 5325.12)	1005.33 (1000.59, 1010.07)	860.41 (852.46, 868.36)	931.10 (927.40, 934.81)
Turkmenistan	678.10 (465.29, 890.92)	487.14 (359.91, 614.37)	574.34 (428.41, 720.27)	13770.75 (8692.51, 18848.98)	8995.07 (6533.94, 11456.20)	11295.68 (8044.48, 14546.89)	1357.43 (1337.93, 1376.94)	978.17 (969.60, 986.75)	1154.52 (1141.21, 1167.84)
Tuvalu	477.00 (454.73, 499.27)	392.68 (378.21, 407.15)	438.95 (425.24, 452.65)	11706.30 (11137.91, 12274.69)	8143.33 (7748.50, 8538.16)	10051.28 (9678.15, 10424.41)	699.33 (692.71, 705.95)	641.64 (640.25, 643.03)	670.88 (667.70, 674.06)
Uganda	248.07 (238.23, 257.92)	222.23 (207.80, 236.65)	236.18 (224.47, 247.89)	5143.66 (4854.77, 5432.56)	4016.88 (3688.66, 4345.11)	4557.08 (4259.86, 4854.29)	634.33 (622.66, 645.99)	566.27 (554.23, 578.30)	594.63 (582.87, 606.39)
Ukraine	778.68 (505.37,	460.12 (346.90,	568.99 (392.03,	12772.45 (5242.98,	7272.19 (5100.79,	9317.48 (4900.55,	1249.06 (1212.20,	1017.02 (991.11,	1125.19 (1099.00,

	1051.98)	573.34)	745.94)	20301.92)	9443.58)	13734.41)	1285.91)	1042.92)	1151.38)
United Arab Emirates	282.89 (172.68, 393.09)	570.82 (114.20, 1027.44)	375.89 (214.19, 537.58)	5859.43 (4114.42, 7604.44)	9249.46 (2720.01, 15778.92)	7183.86 (4693.44, 9674.27)	1167.61 (1163.00, 1172.22)	994.24 (967.15, 1021.32)	1134.65 (1125.81, 1143.48)
United Kingdom	167.21 (145.55, 188.87)	109.21 (94.47, 123.95)	135.64 (119.30, 151.98)	3088.22 (2812.56, 3363.89)	1786.10 (1631.38, 1940.83)	2418.11 (2216.98, 2619.24)	688.10 (671.77, 704.42)	523.55 (515.83, 531.26)	604.76 (593.15, 616.37)
United Republic of Tanzania	275.88 (260.88, 290.89)	239.05 (226.38, 251.73)	255.94 (242.33, 269.56)	5678.26 (5278.17, 6078.35)	4566.12 (4309.20, 4823.04)	5077.28 (4760.26, 5394.30)	676.51 (668.28, 684.74)	579.31 (574.55, 584.06)	624.89 (618.29, 631.49)
United States Virgin Islands	324.26 (202.12, 446.39)	215.12 (200.11, 230.14)	262.35 (201.15, 323.56)	6602.79 (4187.41, 9018.17)	3536.73 (3278.69, 3794.76)	4893.53 (3695.43, 6091.64)	729.88 (719.15, 740.61)	616.36 (609.77, 622.95)	667.16 (664.98, 669.33)
United States of America	204.91 (189.92, 219.89)	120.02 (106.83, 133.20)	158.40 (144.77, 172.02)	4149.37 (3854.89, 4443.86)	2368.40 (2180.80, 2555.99)	3198.36 (2968.60, 3428.12)	786.62 (779.47, 793.77)	637.70 (628.35, 647.06)	705.86 (697.90, 713.82)
Uruguay	196.62 (149.43, 243.81)	127.65 (99.19, 156.11)	154.56 (123.71, 185.41)	3647.86 (2914.20, 4381.52)	2149.68 (1793.52, 2505.83)	2788.58 (2326.24, 3250.93)	631.87 (625.47, 638.27)	520.25 (508.34, 532.17)	572.87 (564.13, 581.61)
Uzbekistan	723.16 (567.30, 879.03)	447.82 (316.48, 579.17)	566.95 (426.69, 707.21)	12678.12 (9554.75, 15801.49)	8296.46 (6139.07, 10453.84)	10292.72 (7771.54, 12813.90)	1518.10 (1414.10, 1622.10)	1247.14 (1165.60, 1328.67)	1367.26 (1273.29, 1461.22)
Vanuatu	636.88 (593.78, 679.98)	488.54 (444.67, 532.41)	566.19 (522.84, 609.54)	16103.40 (14817.00, 17389.80)	10606.55 (9283.75, 11929.35)	13455.07 (12153.99, 14756.14)	823.94 (817.16, 830.72)	768.80 (762.14, 775.45)	798.27 (791.53, 805.00)
Venezuela	359.44	187.73	271.50	7388.47	3362.90	5345.16	500.04	454.75	476.36

(Bolivarian Republic of)	(244.84, 474.04)	(143.15, 232.32)	(211.63, 331.37)	(5345.63, 9431.32)	(2510.76, 4215.04)	(4276.06, 6414.25)	(485.92, 514.17)	(447.86, 461.63)	(468.16, 484.56)
Viet Nam	386.98 (380.84, 393.13)	181.98 (178.99, 184.98)	262.94 (259.83, 266.05)	7537.55 (7361.01, 7714.09)	3217.98 (3157.22, 3278.74)	5109.36 (5013.05, 5205.66)	730.31 (728.22, 732.40)	557.59 (551.99, 563.20)	634.32 (630.28, 638.36)
Yemen	603.86 (584.46, 623.27)	513.27 (502.72, 523.82)	557.51 (542.90, 572.12)	12686.54 (12179.86, 13193.22)	10138.47 (9802.18, 10474.77)	11384.13 (10967.76, 11800.50)	1144.14 (1134.69, 1153.59)	994.25 (990.00, 998.51)	1067.25 (1059.83, 1074.67)
Zambia	333.11 (309.89, 356.33)	280.25 (264.27, 296.24)	307.84 (289.33, 326.36)	6853.81 (6319.69, 7387.94)	5122.79 (4777.37, 5468.22)	6014.28 (5603.54, 6425.03)	617.55 (614.50, 620.59)	550.23 (544.64, 555.82)	581.70 (578.23, 585.18)
Zimbabwe	318.72 (302.36, 335.07)	244.42 (190.48, 298.37)	275.52 (240.22, 310.82)	6767.73 (6280.96, 7254.50)	4585.21 (3488.85, 5681.56)	5512.09 (4770.35, 6253.83)	690.18 (683.88, 696.49)	634.93 (611.42, 658.44)	658.51 (644.76, 672.26)
Germany	154.16 (126.61, 181.72)	116.52 (95.20, 137.85)	136.95 (113.87, 160.03)	2741.94 (2361.25, 3122.63)	1770.14 (1520.71, 2019.57)	2248.59 (1944.85, 2552.34)	552.52 (514.79, 590.26)	384.17 (355.43, 412.90)	464.26 (431.14, 497.37)
Ghana	253.50 (238.90, 268.10)	306.64 (289.54, 323.74)	288.32 (273.60, 303.03)	4948.44 (4615.91, 5280.97)	5459.43 (5083.14, 5835.72)	5289.86 (4968.97, 5610.74)	668.27 (662.70, 673.84)	627.16 (613.09, 641.23)	644.65 (636.30, 653.01)

STable 7. The EAPC of projected age-standardized rates of CVD from 2020-2030 and the EAPC from 1990-2019, by different countries and genders.

	1990-2019			2020-2030		
location	DALYs (95%CI)	Deaths(95%CI)	Incidence(95%CI)	DALYs (95%CI)	Deaths(95%CI)	Incidence(95%CI)
Afghanistan	-1.08 (-1.27, -0.89)	-0.87 (-1.02, -0.71)	-0.33 (-0.38, -0.27)	-0.83 (-0.84, -0.82)	-0.52 (-0.52, -0.52)	0.01 (0.01, 0.01)
Albania	-0.72 (-0.88, -0.56)	-0.54 (-0.72, -0.36)	-0.18 (-0.20, -0.15)	-0.74 (-0.74, -0.73)	-0.63 (-0.63, -0.62)	0.14 (0.14, 0.14)
Algeria	-2.06 (-2.13, -1.99)	-1.77 (-1.83, -1.70)	-0.67 (-0.72, -0.62)	-1.06 (-1.07, -1.05)	-0.92 (-0.93, -0.91)	0.08 (0.08, 0.08)
American Samoa	-0.57 (-0.66, -0.48)	-0.57 (-0.65, -0.48)	0.09 (0.01, 0.17)	-0.59 (-0.59, -0.59)	-0.69 (-0.69, -0.68)	0.38 (0.38, 0.39)
Andorra	-1.62 (-1.79, -1.45)	-1.52 (-1.71, -1.33)	-0.93 (-1.02, -0.83)	-0.42 (-0.43, -0.42)	-0.51 (-0.51, -0.51)	0.47 (0.47, 0.47)
Angola	-1.15 (-1.25, -1.05)	-0.82 (-0.92, -0.72)	-0.39 (-0.42, -0.36)	0.01 (0.01, 0.01)	0.46 (0.46, 0.46)	0.07 (0.07, 0.07)
Antigua and Barbuda	-1.48 (-1.69, -1.28)	-1.20 (-1.40, -0.99)	-0.22 (-0.24, -0.19)	-1.36 (-1.37, -1.34)	-1.48 (-1.50, -1.46)	0.21 (0.21, 0.21)
Argentina	-2.34 (-2.52, -2.16)	-2.16 (-2.37, -1.96)	-1.06 (-1.15, -0.98)	-0.13 (-0.13, -0.13)	-0.01 (-0.01, -0.01)	0.28 (0.28, 0.28)
Armenia	-1.92 (-2.11, -1.73)	-1.94 (-2.16, -1.73)	-0.56 (-0.61, -0.52)	-0.37 (-0.38, -0.37)	-0.29 (-0.29, -0.29)	0.25 (0.24, 0.25)
Australia	-3.60 (-3.81, -3.40)	-3.61 (-3.80, -3.43)	-0.89 (-0.98, -0.80)	-0.27 (-0.27, -0.27)	-0.15 (-0.15, -0.15)	0.27 (0.27, 0.27)
Austria	-3.20 (-3.37, -3.03)	-3.01 (-3.15,	-0.64 (-0.69,	-1.60 (-1.62, -1.58)	-1.48 (-1.50, -1.46)	-2.10 (-2.15,

		-2.87)	-0.60)			-2.06)
Azerbaijan	0.10 (-0.11, 0.32)	1.08 (0.85, 1.30)	0.37 (0.32, 0.41)	-3.43 (-3.53, -3.32)	-5.45 (-5.73, -5.18)	-0.07 (-0.07, -0.07)
Bahamas	-0.95 (-1.06, -0.84)	-1.02 (-1.14, -0.89)	-0.06 (-0.08, -0.04)	-0.14 (-0.14, -0.14)	-0.26 (-0.26, -0.26)	0.21 (0.21, 0.22)
Bahrain	-3.83 (-4.04, -3.62)	-3.22 (-3.47, -2.98)	-0.38 (-0.41, -0.35)	1.19 (1.18, 1.20)	-7.26 (-7.74, -6.77)	0.32 (0.32, 0.32)
Bangladesh	-0.41 (-0.73, -0.09)	-0.20 (-0.64, 0.23)	0.04 (-0.04, 0.12)	-1.19 (-1.20, -1.18)	-0.76 (-0.76, -0.75)	-0.36 (-0.36, -0.36)
Barbados	-1.66 (-1.85, -1.46)	-1.70 (-1.91, -1.48)	-0.03 (-0.06, -0.01)	0.65 (0.64, 0.65)	0.78 (0.78, 0.79)	0.15 (0.15, 0.15)
Belarus	-0.64 (-1.09, -0.20)	-0.52 (-0.88, -0.16)	-0.09 (-0.21, 0.03)	-0.64 (-0.65, -0.64)	-1.21 (-1.22, -1.20)	-0.49 (-0.49, -0.49)
Belgium	-2.98 (-3.08, -2.88)	-2.90 (-3.00, -2.79)	-1.19 (-1.23, -1.15)	0.02 (0.02, 0.02)	-0.13 (-0.13, -0.13)	-0.34 (-0.35, -0.34)
Belize	-1.41 (-1.77, -1.06)	-1.50 (-1.87, -1.13)	0.01 (-0.02, 0.05)	1.22 (1.20, 1.23)	1.57 (1.55, 1.59)	0.20 (0.20, 0.21)
Benin	-0.62 (-0.69, -0.56)	-0.49 (-0.55, -0.44)	-0.07 (-0.09, -0.05)	-1.78 (-1.81, -1.75)	-1.52 (-1.54, -1.50)	0.00 (0.00, 0.00)
Bermuda	-3.37 (-3.67, -3.07)	-3.29 (-3.56, -3.01)	-0.31 (-0.34, -0.28)	-0.78 (-0.79, -0.78)	-0.79 (-0.79, -0.78)	0.36 (0.35, 0.36)
Bhutan	-0.92 (-0.94, -0.89)	-0.49 (-0.51, -0.47)	-0.19 (-0.20, -0.18)	-0.69 (-0.69, -0.68)	-0.34 (-0.34, -0.34)	0.07 (0.07, 0.07)
Bolivia (Plurinational State of)	-1.59 (-1.78, -1.41)	-1.22 (-1.42, -1.02)	-0.37 (-0.44, -0.30)	-0.63 (-0.64, -0.63)	-0.21 (-0.21, -0.21)	-0.23 (-0.23, -0.23)
Bosnia and Herzegovina	-1.58 (-1.73, -1.43)	-1.14 (-1.33,	0.06 (0.04, 0.09)	-1.66 (-1.68, -1.63)	-2.09 (-2.13, -2.05)	0.19 (0.19, 0.19)

		-0.94)				
Botswana	-0.79 (-1.13, -0.44)	-0.59 (-0.87, -0.30)	-0.12 (-0.13, -0.11)	-1.73 (-1.76, -1.70)	-1.62 (-1.64, -1.59)	-0.08 (-0.08, -0.08)
Brazil	-2.41 (-2.48, -2.34)	-2.44 (-2.54, -2.35)	-0.81 (-0.87, -0.75)	-1.87 (-1.90, -1.83)	-1.79 (-1.82, -1.76)	-0.03 (-0.03, -0.03)
Brunei Darussalam	-1.99 (-2.13, -1.86)	-1.70 (-1.82, -1.57)	-1.15 (-1.24, -1.05)	-0.31 (-0.31, -0.31)	-0.94 (-0.95, -0.93)	0.13 (0.13, 0.13)
Bulgaria	-1.60 (-1.90, -1.29)	-1.55 (-1.85, -1.25)	-0.95 (-1.07, -0.82)	-0.03 (-0.03, -0.03)	0.04 (0.04, 0.04)	0.17 (0.17, 0.17)
Burkina Faso	0.47 (0.39, 0.56)	0.69 (0.55, 0.84)	0.06 (0.05, 0.08)	-1.18 (-1.19, -1.16)	-1.38 (-1.40, -1.36)	-0.08 (-0.08, -0.08)
Burundi	-1.67 (-1.79, -1.55)	-1.33 (-1.41, -1.24)	-0.56 (-0.60, -0.51)	0.39 (0.39, 0.39)	0.37 (0.37, 0.37)	0.15 (0.15, 0.15)
Cabo Verde	-0.55 (-0.78, -0.31)	-0.24 (-0.56, 0.08)	0.02 (0.01, 0.03)	4.08 (3.93, 4.23)	4.82 (4.61, 5.04)	0.08 (0.08, 0.08)
Cambodia	-1.03 (-1.10, -0.96)	-0.60 (-0.66, -0.53)	-0.29 (-0.32, -0.26)	-0.28 (-0.28, -0.28)	0.12 (0.12, 0.12)	0.02 (0.02, 0.02)
Cameroon	-0.04 (-0.25, 0.17)	-0.03 (-0.21, 0.15)	0.25 (0.15, 0.35)	-1.90 (-1.94, -1.87)	-1.69 (-1.72, -1.67)	-0.10 (-0.10, -0.10)
Canada	-3.02 (-3.22, -2.82)	-3.24 (-3.45, -3.03)	-1.41 (-1.53, -1.29)	0.62 (0.61, 0.62)	0.97 (0.97, 0.98)	0.09 (0.09, 0.09)
Central African Republic	-0.36 (-0.40, -0.32)	-0.21 (-0.25, -0.18)	-0.01 (-0.02, -0.00)	-0.70 (-0.70, -0.69)	-0.42 (-0.42, -0.41)	0.09 (0.09, 0.09)
Chad	0.03 (-0.10, 0.15)	0.07 (-0.03, 0.17)	0.11 (0.08, 0.13)	-0.88 (-0.89, -0.87)	-0.81 (-0.82, -0.81)	-0.04 (-0.04, -0.04)
Chile	-2.26 (-2.38, -2.15)	-2.33 (-2.46,	-0.85 (-1.05,	-0.68 (-0.68, -0.68)	-0.12 (-0.12, -0.12)	1.68 (1.66, 1.71)

		-2.21)	-0.65)			
China	-1.26 (-1.35, -1.17)	-0.89 (-1.01, -0.77)	0.02 (-0.01, 0.05)	-1.60 (-1.62, -1.58)	-1.65 (-1.67, -1.62)	0.07 (0.07, 0.07)
Colombia	-2.91 (-3.05, -2.76)	-2.83 (-2.97, -2.69)	-0.98 (-1.05, -0.92)	-1.98 (-2.01, -1.94)	-1.76 (-1.78, -1.73)	-0.33 (-0.33, -0.33)
Comoros	-1.17 (-1.34, -1.00)	-0.92 (-1.05, -0.80)	-0.51 (-0.57, -0.46)	-2.28 (-2.32, -2.23)	-1.06 (-1.08, -1.05)	0.07 (0.07, 0.07)
Congo	-1.45 (-1.57, -1.32)	-1.09 (-1.20, -0.98)	-0.42 (-0.46, -0.38)	-0.78 (-0.78, -0.77)	-0.59 (-0.59, -0.58)	0.10 (0.10, 0.10)
Cook Islands	-1.41 (-1.61, -1.21)	-1.41 (-1.60, -1.23)	-0.03 (-0.05, -0.00)	-0.95 (-0.96, -0.94)	-0.95 (-0.96, -0.94)	0.43 (0.43, 0.44)
Costa Rica	-1.98 (-2.20, -1.76)	-2.19 (-2.43, -1.96)	-0.30 (-0.32, -0.29)	0.15 (0.14, 0.15)	0.34 (0.34, 0.35)	0.04 (0.04, 0.04)
Croatia	-2.51 (-2.65, -2.37)	-2.10 (-2.24, -1.97)	-0.72 (-0.84, -0.61)	-1.59 (-1.62, -1.57)	-1.99 (-2.03, -1.95)	0.42 (0.41, 0.42)
Cuba	-1.77 (-1.95, -1.58)	-1.75 (-1.95, -1.55)	-0.52 (-0.58, -0.47)	-0.18 (-0.18, -0.18)	-0.04 (-0.04, -0.04)	0.42 (0.41, 0.42)
Cyprus	-3.33 (-3.49, -3.17)	-3.27 (-3.41, -3.12)	-1.08 (-1.15, -1.01)	-0.10 (-0.10, -0.10)	-0.83 (-0.84, -0.83)	1.14 (1.12, 1.15)
Czechia	-3.33 (-3.45, -3.22)	-3.07 (-3.18, -2.96)	-1.03 (-1.13, -0.93)	-0.78 (-0.79, -0.77)	-0.72 (-0.72, -0.71)	0.06 (0.06, 0.06)
Côte d'Ivoire	-0.76 (-0.91, -0.61)	-0.62 (-0.74, -0.49)	-0.11 (-0.17, -0.05)	-2.42 (-2.47, -2.36)	-2.07 (-2.11, -2.03)	-0.01 (-0.01, -0.01)
Democratic People's Republic of Korea	-0.01 (-0.18, 0.16)	0.02 (-0.18, 0.23)	0.17 (0.12, 0.22)	-1.34 (-1.36, -1.33)	-1.34 (-1.36, -1.33)	0.00 (0.00, 0.00)
Democratic Republic of the	-0.60 (-0.65, -0.54)	-0.45 (-0.50,	-0.27 (-0.29,	-1.07 (-1.09, -1.06)	-0.12 (-0.12, -0.12)	0.16 (0.16, 0.16)

Congo		-0.39)	-0.24)			
Denmark	-4.08 (-4.27, -3.89)	-3.87 (-4.05, -3.68)	-1.38 (-1.43, -1.33)	-0.20 (-0.20, -0.20)	-0.71 (-0.71, -0.70)	0.05 (0.05, 0.05)
Djibouti	-0.54 (-0.59, -0.50)	-0.36 (-0.40, -0.33)	-0.14 (-0.15, -0.12)	0.05 (0.05, 0.05)	0.34 (0.34, 0.34)	0.13 (0.13, 0.13)
Dominica	-0.85 (-1.03, -0.67)	-0.81 (-0.99, -0.63)	0.04 (0.03, 0.06)	-0.32 (-0.32, -0.32)	-0.34 (-0.34, -0.34)	0.09 (0.09, 0.09)
Dominican Republic	1.20 (0.96, 1.45)	1.44 (1.16, 1.73)	0.30 (0.28, 0.33)	-3.50 (-3.61, -3.39)	-3.40 (-3.50, -3.29)	0.35 (0.35, 0.36)
Ecuador	-1.02 (-1.27, -0.77)	-0.61 (-0.91, -0.30)	-0.08 (-0.13, -0.02)	-0.15 (-0.15, -0.15)	-0.35 (-0.35, -0.35)	0.39 (0.39, 0.39)
Egypt	-0.64 (-0.74, -0.54)	-0.35 (-0.46, -0.24)	0.09 (0.02, 0.16)	-2.90 (-2.98, -2.82)	-2.95 (-3.03, -2.87)	-0.17 (-0.17, -0.17)
El Salvador	-1.71 (-2.00, -1.43)	-1.34 (-1.58, -1.10)	-0.13 (-0.15, -0.10)	-0.96 (-0.97, -0.95)	-0.63 (-0.63, -0.63)	0.15 (0.15, 0.15)
Equatorial Guinea	-2.58 (-2.88, -2.29)	-1.94 (-2.19, -1.70)	-0.72 (-0.79, -0.65)	0.38 (0.38, 0.38)	0.62 (0.62, 0.63)	0.17 (0.17, 0.17)
Eritrea	-0.61 (-0.65, -0.56)	-0.21 (-0.26, -0.17)	-0.34 (-0.37, -0.31)	-0.80 (-0.81, -0.79)	-0.43 (-0.43, -0.43)	0.23 (0.23, 0.23)
Estonia	-3.52 (-3.81, -3.23)	-3.18 (-3.40, -2.95)	-0.48 (-0.55, -0.42)	-1.13 (-1.14, -1.12)	-0.87 (-0.88, -0.86)	0.79 (0.79, 0.80)
Eswatini	0.13 (-0.27, 0.53)	0.19 (-0.16, 0.54)	-0.01 (-0.06, 0.05)	-1.52 (-1.55, -1.50)	-1.24 (-1.26, -1.23)	-0.05 (-0.05, -0.05)
Ethiopia	-2.38 (-2.50, -2.26)	-1.77 (-1.87, -1.67)	-0.37 (-0.40, -0.35)	-0.04 (-0.04, -0.04)	0.25 (0.24, 0.25)	0.20 (0.20, 0.20)
Fiji	-0.98 (-1.09, -0.88)	-0.92 (-1.05, -0.78)	-0.01 (-0.06, 0.04)	-0.87 (-0.88, -0.87)	-0.96 (-0.96, -0.95)	-0.02 (-0.02, -0.02)

Finland	-2.89 (-3.00, -2.78)	-2.48 (-2.57, -2.39)	-1.35 (-1.41, -1.29)	-0.31 (-0.31, -0.31)	-0.41 (-0.41, -0.41)	0.13 (0.13, 0.13)
France	-2.74 (-2.84, -2.64)	-2.85 (-2.96, -2.74)	-1.21 (-1.28, -1.14)	-0.32 (-0.32, -0.32)	-0.04 (-0.04, -0.04)	0.19 (0.19, 0.19)
Gabon	-1.02 (-1.12, -0.91)	-0.78 (-0.87, -0.69)	-0.14 (-0.15, -0.12)	-1.43 (-1.45, -1.41)	-0.98 (-0.99, -0.97)	0.11 (0.11, 0.11)
Gambia	0.24 (0.10, 0.38)	0.35 (0.25, 0.46)	0.13 (0.12, 0.15)	0.67 (0.67, 0.68)	0.74 (0.74, 0.75)	-0.02 (-0.02, -0.02)
Georgia	-1.75 (-3.01, -0.47)	-1.75 (-3.07, -0.41)	-1.09 (-1.57, -0.61)	-2.52 (-2.58, -2.46)	-2.47 (-2.53, -2.41)	-1.31 (-1.33, -1.29)
Germany	-3.08 (-3.31, -2.84)	-2.84 (-3.08, -2.61)	-1.72 (-1.82, -1.61)	-1.31 (-1.32, -1.29)	-1.35 (-1.37, -1.33)	-2.47 (-2.52, -2.41)
Ghana	-0.01 (-0.11, 0.10)	0.10 (0.01, 0.18)	0.17 (0.14, 0.20)	-1.47 (-1.49, -1.45)	-0.68 (-0.68, -0.67)	-0.10 (-0.10, -0.10)
Greece	-2.06 (-2.18, -1.94)	-2.27 (-2.45, -2.08)	-1.21 (-1.27, -1.14)	1.65 (1.63, 1.68)	2.54 (2.48, 2.60)	-0.01 (-0.01, -0.01)
Greenland	-2.78 (-2.96, -2.59)	-2.85 (-3.09, -2.61)	-1.15 (-1.18, -1.12)	-1.16 (-1.17, -1.14)	-1.26 (-1.28, -1.25)	-0.40 (-0.40, -0.40)
Grenada	-1.66 (-1.90, -1.41)	-1.60 (-1.83, -1.38)	-0.06 (-0.09, -0.03)	-1.86 (-1.89, -1.83)	-1.75 (-1.78, -1.72)	0.10 (0.10, 0.10)
Guam	-0.84 (-1.08, -0.60)	-1.58 (-1.94, -1.22)	0.25 (0.23, 0.28)	-0.30 (-0.30, -0.30)	-0.10 (-0.11, -0.10)	0.43 (0.43, 0.43)
Guatemala	-2.06 (-2.37, -1.75)	-1.73 (-1.98, -1.48)	-0.22 (-0.27, -0.16)	0.02 (0.02, 0.02)	0.33 (0.32, 0.33)	-0.13 (-0.13, -0.13)
Guinea	0.40 (0.31, 0.49)	0.44 (0.35, 0.54)	0.36 (0.31, 0.40)	-2.34 (-2.39, -2.29)	-1.85 (-1.88, -1.82)	-0.07 (-0.07, -0.07)

Guinea-Bissau	-0.24 (-0.32, -0.15)	-0.07 (-0.14, 0.00)	-0.02 (-0.03, -0.00)	-2.08 (-2.12, -2.04)	-1.46 (-1.48, -1.44)	-0.04 (-0.04, -0.04)
Guyana	-1.45 (-1.61, -1.28)	-1.40 (-1.58, -1.22)	-0.31 (-0.33, -0.29)	-1.83 (-1.86, -1.80)	-1.79 (-1.82, -1.76)	0.16 (0.16, 0.16)
Haiti	-0.92 (-1.06, -0.77)	-0.73 (-0.84, -0.62)	-0.13 (-0.15, -0.11)	-1.08 (-1.09, -1.07)	-0.79 (-0.80, -0.79)	0.18 (0.18, 0.18)
Honduras	0.39 (0.25, 0.53)	0.93 (0.73, 1.14)	-0.04 (-0.06, -0.02)	-0.25 (-0.25, -0.24)	-0.17 (-0.17, -0.17)	-0.13 (-0.13, -0.13)
Hungary	-2.50 (-2.62, -2.39)	-2.16 (-2.25, -2.06)	-0.85 (-0.88, -0.81)	-0.71 (-0.72, -0.71)	-1.07 (-1.08, -1.06)	0.22 (0.22, 0.22)
Iceland	-3.37 (-3.50, -3.24)	-3.24 (-3.36, -3.13)	-0.92 (-0.97, -0.88)	-0.67 (-0.67, -0.66)	-0.58 (-0.59, -0.58)	-2.13 (-2.17, -2.08)
India	-0.96 (-1.06, -0.86)	-1.02 (-1.13, -0.92)	-0.10 (-0.16, -0.04)	-0.80 (-0.80, -0.79)	-0.74 (-0.75, -0.74)	-0.01 (-0.01, -0.01)
Indonesia	0.26 (0.18, 0.34)	0.61 (0.52, 0.69)	0.08 (0.01, 0.16)	-1.29 (-1.31, -1.28)	-0.92 (-0.93, -0.91)	-0.11 (-0.11, -0.11)
Iran (Islamic Republic of)	-2.25 (-2.40, -2.11)	-2.06 (-2.22, -1.90)	-0.40 (-0.46, -0.34)	-1.57 (-1.59, -1.54)	-1.70 (-1.73, -1.68)	0.92 (0.91, 0.93)
Iraq	-1.22 (-1.34, -1.10)	-0.90 (-0.99, -0.82)	-0.39 (-0.41, -0.36)	0.49 (0.49, 0.50)	1.09 (1.08, 1.10)	0.01 (0.01, 0.01)
Ireland	-4.17 (-4.40, -3.95)	-3.88 (-4.10, -3.66)	-1.67 (-1.79, -1.56)	-0.76 (-0.77, -0.76)	-1.00 (-1.01, -0.99)	0.26 (0.25, 0.26)
Israel	-4.32 (-4.59, -4.05)	-4.23 (-4.48, -3.97)	-1.37 (-1.45, -1.29)	1.15 (1.14, 1.16)	1.19 (1.18, 1.20)	0.08 (0.08, 0.08)
Italy	-2.94 (-3.09, -2.79)	-2.78 (-2.90, -2.65)	-0.96 (-1.08, -0.83)	-0.36 (-0.36, -0.36)	-0.48 (-0.48, -0.48)	0.82 (0.81, 0.82)

Jamaica	-0.69 (-1.08, -0.30)	-0.79 (-1.17, -0.40)	0.32 (0.29, 0.35)	-4.82 (-5.03, -4.61)	-3.95 (-4.09, -3.81)	0.42 (0.42, 0.42)
Japan	-2.48 (-2.60, -2.36)	-3.13 (-3.32, -2.95)	-0.84 (-0.94, -0.73)	-0.14 (-0.14, -0.13)	0.18 (0.18, 0.18)	1.04 (1.03, 1.05)
Jordan	-2.75 (-2.99, -2.50)	-2.59 (-2.82, -2.36)	-0.49 (-0.54, -0.44)	0.86 (0.86, 0.87)	1.07 (1.06, 1.08)	0.64 (0.64, 0.65)
Kazakhstan	-1.17 (-1.85, -0.50)	-0.84 (-1.40, -0.27)	-0.69 (-0.92, -0.47)	-1.87 (-1.90, -1.84)	-2.70 (-2.77, -2.64)	-0.37 (-0.37, -0.37)
Kenya	0.29 (0.11, 0.47)	0.37 (0.23, 0.50)	-0.19 (-0.21, -0.16)	-0.23 (-0.23, -0.23)	0.09 (0.09, 0.09)	0.17 (0.17, 0.17)
Kiribati	-0.80 (-0.85, -0.74)	-0.55 (-0.61, -0.50)	0.03 (0.02, 0.05)	-0.58 (-0.58, -0.57)	-0.35 (-0.35, -0.35)	-0.04 (-0.04, -0.04)
Kuwait	-1.43 (-1.78, -1.08)	-1.35 (-1.69, -1.01)	0.06 (-0.00, 0.13)	-0.92 (-0.93, -0.92)	-0.45 (-0.45, -0.45)	0.47 (0.47, 0.47)
Kyrgyzstan	-0.54 (-0.96, -0.11)	0.19 (-0.28, 0.66)	-0.14 (-0.21, -0.07)	-3.48 (-3.59, -3.37)	-5.36 (-5.62, -5.09)	-0.24 (-0.24, -0.24)
Lao People's Democratic Republic	-0.82 (-0.94, -0.69)	-0.57 (-0.68, -0.46)	-0.42 (-0.46, -0.39)	-0.48 (-0.48, -0.48)	0.18 (0.18, 0.18)	-0.07 (-0.07, -0.07)
Latvia	-2.14 (-2.46, -1.82)	-1.93 (-2.17, -1.69)	-0.48 (-0.53, -0.43)	-1.75 (-1.77, -1.72)	-1.78 (-1.81, -1.75)	0.52 (0.52, 0.52)
Lebanon	-1.18 (-1.38, -0.97)	-1.07 (-1.22, -0.91)	-0.03 (-0.12, 0.06)	-1.61 (-1.63, -1.59)	-1.89 (-1.92, -1.85)	0.03 (0.03, 0.03)
Lesotho	1.55 (1.25, 1.85)	1.52 (1.24, 1.80)	0.28 (0.24, 0.32)	-2.09 (-2.13, -2.05)	-1.80 (-1.83, -1.77)	-0.13 (-0.13, -0.13)
Liberia	-0.77 (-0.89, -0.64)	-0.58 (-0.68, -0.48)	-0.02 (-0.05, 0.02)	-0.72 (-0.73, -0.72)	-0.79 (-0.79, -0.78)	-0.01 (-0.01, -0.01)

Libya	-0.61 (-0.77, -0.46)	-0.34 (-0.50, -0.17)	0.19 (0.16, 0.22)	-0.93 (-0.94, -0.92)	-0.85 (-0.86, -0.84)	0.68 (0.67, 0.68)
Lithuania	-1.56 (-1.84, -1.28)	-1.37 (-1.59, -1.16)	-0.44 (-0.48, -0.41)	-5.37 (-5.64, -5.11)	-4.25 (-4.41, -4.08)	0.22 (0.22, 0.22)
Luxembourg	-3.78 (-3.87, -3.69)	-3.70 (-3.77, -3.62)	-0.84 (-0.92, -0.76)	0.75 (0.75, 0.76)	0.87 (0.87, 0.88)	-0.91 (-0.92, -0.91)
Madagascar	-0.46 (-0.55, -0.37)	-0.27 (-0.39, -0.14)	-0.06 (-0.10, -0.03)	-0.67 (-0.67, -0.66)	-0.15 (-0.15, -0.15)	-0.10 (-0.10, -0.10)
Malawi	-0.83 (-0.99, -0.68)	-0.64 (-0.77, -0.52)	-0.20 (-0.24, -0.16)	-0.38 (-0.38, -0.38)	-0.10 (-0.10, -0.10)	0.18 (0.18, 0.18)
Malaysia	-1.34 (-1.46, -1.23)	-1.40 (-1.59, -1.20)	-0.53 (-0.57, -0.49)	0.23 (0.23, 0.23)	-0.48 (-0.49, -0.48)	-0.17 (-0.17, -0.17)
Maldives	-3.72 (-3.97, -3.47)	-3.30 (-3.51, -3.08)	-0.86 (-0.93, -0.79)	-0.17 (-0.17, -0.17)	-0.26 (-0.26, -0.26)	0.13 (0.13, 0.13)
Mali	-0.71 (-0.81, -0.62)	-0.51 (-0.57, -0.44)	-0.13 (-0.17, -0.09)	-1.43 (-1.45, -1.41)	-1.16 (-1.17, -1.15)	-0.05 (-0.05, -0.05)
Malta	-2.98 (-3.06, -2.89)	-2.88 (-2.96, -2.80)	-0.91 (-1.05, -0.77)	-0.91 (-0.92, -0.90)	-0.76 (-0.77, -0.76)	-2.30 (-2.35, -2.25)
Marshall Islands	-0.07 (-0.17, 0.04)	-0.05 (-0.15, 0.04)	0.01 (-0.03, 0.05)	-0.49 (-0.49, -0.49)	-0.38 (-0.38, -0.38)	-0.04 (-0.04, -0.04)
Mauritania	-1.59 (-1.71, -1.47)	-1.38 (-1.49, -1.26)	-0.31 (-0.34, -0.27)	-1.08 (-1.09, -1.06)	-0.44 (-0.45, -0.44)	0.00 (0.00, 0.00)
Mauritius	-3.70 (-4.05, -3.34)	-3.59 (-3.94, -3.23)	-1.00 (-1.08, -0.93)	-0.69 (-0.69, -0.68)	0.15 (0.15, 0.15)	0.09 (0.09, 0.09)
Mexico	-0.93 (-1.10, -0.76)	-0.97 (-1.17, -0.77)	-0.50 (-0.56, -0.44)	-0.67 (-0.67, -0.66)	-1.23 (-1.24, -1.21)	0.07 (0.07, 0.07)

Micronesia (Federated States of)	-0.32 (-0.39, -0.26)	-0.18 (-0.24, -0.13)	-0.23 (-0.24, -0.22)	-0.12 (-0.12, -0.12)	0.01 (0.01, 0.01)	-0.03 (-0.03, -0.03)
Monaco	-2.16 (-2.39, -1.92)	-2.14 (-2.36, -1.91)	-0.77 (-0.85, -0.69)	-1.37 (-1.39, -1.35)	-1.42 (-1.43, -1.40)	0.42 (0.42, 0.43)
Mongolia	-1.28 (-1.69, -0.87)	-0.95 (-1.37, -0.53)	-0.60 (-0.73, -0.47)	0.41 (0.40, 0.41)	0.43 (0.43, 0.43)	0.21 (0.21, 0.21)
Montenegro	-0.12 (-0.34, 0.10)	0.31 (0.15, 0.48)	-0.01 (-0.04, 0.01)	-3.38 (-3.48, -3.27)	-5.43 (-5.70, -5.16)	0.24 (0.24, 0.24)
Morocco	-0.70 (-0.80, -0.60)	-0.42 (-0.60, -0.25)	-0.20 (-0.24, -0.17)	-1.00 (-1.01, -0.99)	-1.01 (-1.02, -1.00)	0.08 (0.08, 0.08)
Mozambique	0.78 (0.59, 0.97)	0.72 (0.55, 0.89)	0.19 (0.17, 0.22)	-1.08 (-1.09, -1.07)	-0.84 (-0.84, -0.83)	0.12 (0.12, 0.12)
Myanmar	-1.66 (-1.77, -1.54)	-1.24 (-1.33, -1.14)	-0.50 (-0.52, -0.48)	-1.05 (-1.06, -1.04)	-0.51 (-0.52, -0.51)	-0.08 (-0.08, -0.08)
Namibia	-1.01 (-1.28, -0.75)	-0.78 (-1.02, -0.53)	-0.50 (-0.54, -0.46)	-0.86 (-0.86, -0.85)	-0.48 (-0.49, -0.48)	0.03 (0.03, 0.03)
Nauru	-0.15 (-0.51, 0.20)	-0.11 (-0.41, 0.20)	0.01 (-0.05, 0.08)	-1.49 (-1.51, -1.47)	-1.35 (-1.37, -1.33)	0.03 (0.03, 0.03)
Nepal	-0.68 (-0.88, -0.48)	-0.28 (-0.46, -0.09)	-0.16 (-0.18, -0.15)	-0.02 (-0.02, -0.02)	0.39 (0.39, 0.39)	0.08 (0.08, 0.08)
Netherlands	-3.62 (-3.88, -3.37)	-3.29 (-3.52, -3.05)	-1.42 (-1.51, -1.32)	0.20 (0.20, 0.20)	-0.07 (-0.07, -0.07)	0.66 (0.65, 0.66)
New Zealand	-3.33 (-3.52, -3.14)	-3.00 (-3.16, -2.85)	-1.21 (-1.28, -1.14)	0.40 (0.40, 0.40)	0.48 (0.48, 0.49)	-1.04 (-1.05, -1.03)
Nicaragua	-0.46 (-0.67, -0.25)	0.10 (-0.23, 0.44)	-0.17 (-0.19, -0.15)	-1.56 (-1.58, -1.54)	-1.76 (-1.79, -1.73)	-0.18 (-0.18, -0.18)
Niger	-0.71 (-0.81, -0.60)	-0.47 (-0.54,	-0.06 (-0.09,	-0.71 (-0.71, -0.70)	-0.59 (-0.60, -0.59)	0.03 (0.03, 0.03)

		-0.39)	-0.03)			
Nigeria	-1.37 (-1.51, -1.22)	-1.28 (-1.41, -1.14)	-0.14 (-0.18, -0.11)	-1.63 (-1.66, -1.61)	-1.43 (-1.45, -1.41)	0.13 (0.13, 0.13)
Niue	-0.84 (-0.92, -0.76)	-0.74 (-0.81, -0.67)	-0.03 (-0.06, 0.01)	-0.63 (-0.63, -0.63)	-0.69 (-0.70, -0.69)	0.19 (0.19, 0.19)
North Macedonia	-0.98 (-1.19, -0.77)	-0.39 (-0.65, -0.12)	-0.24 (-0.30, -0.18)	-0.99 (-1.00, -0.98)	-1.06 (-1.07, -1.05)	-0.12 (-0.12, -0.12)
Northern Mariana Islands	-0.51 (-0.62, -0.40)	-0.47 (-0.61, -0.34)	-0.09 (-0.13, -0.05)	-1.48 (-1.50, -1.46)	-1.60 (-1.62, -1.58)	0.25 (0.25, 0.25)
Norway	-3.77 (-3.89, -3.66)	-3.59 (-3.71, -3.47)	-0.97 (-1.01, -0.93)	-0.20 (-0.20, -0.20)	-0.19 (-0.19, -0.19)	-1.51 (-1.53, -1.49)
Oman	-1.56 (-1.71, -1.41)	-0.98 (-1.12, -0.84)	0.18 (0.12, 0.23)	-12.90 (-14.47, -11.30)	-22.98 (-28.06, -17.55)	-0.17 (-0.17, -0.17)
Pakistan	0.07 (-0.19, 0.33)	0.09 (-0.14, 0.32)	0.21 (0.12, 0.31)	-1.73 (-1.76, -1.71)	-1.30 (-1.32, -1.29)	-0.45 (-0.45, -0.44)
Palau	-0.42 (-0.47, -0.38)	-0.43 (-0.48, -0.38)	0.08 (0.04, 0.11)	-0.32 (-0.32, -0.32)	-0.41 (-0.42, -0.41)	0.19 (0.19, 0.19)
Palestine	-1.49 (-1.61, -1.38)	-1.26 (-1.38, -1.13)	-0.06 (-0.09, -0.02)	-0.93 (-0.93, -0.92)	-1.33 (-1.35, -1.31)	0.31 (0.30, 0.31)
Panama	-1.70 (-1.85, -1.54)	-1.84 (-1.98, -1.69)	-0.27 (-0.28, -0.26)	-1.60 (-1.62, -1.58)	-1.72 (-1.75, -1.69)	0.04 (0.04, 0.04)
Papua New Guinea	0.38 (0.27, 0.49)	0.44 (0.35, 0.54)	0.06 (0.03, 0.09)	-0.04 (-0.04, -0.04)	0.03 (0.03, 0.03)	-0.05 (-0.05, -0.05)
Paraguay	-0.94 (-1.08, -0.79)	-0.90 (-1.06, -0.74)	-0.22 (-0.24, -0.19)	0.05 (0.05, 0.05)	-0.15 (-0.15, -0.15)	-0.14 (-0.14, -0.14)
Peru	-2.76 (-3.05, -2.47)	-2.72 (-3.01,	-0.43 (-0.50,	-0.03 (-0.04, -0.03)	-0.00 (-0.00, -0.00)	-0.25 (-0.25,

		-2.43)	-0.36)			-0.25)
Philippines	2.46 (1.91, 3.02)	1.84 (1.37, 2.32)	-0.07 (-0.14, 0.00)	-2.38 (-2.43, -2.33)	-1.74 (-1.77, -1.71)	0.14 (0.14, 0.14)
Poland	-3.11 (-3.22, -2.99)	-2.88 (-2.99, -2.78)	-1.61 (-1.77, -1.45)	-0.40 (-0.41, -0.40)	-0.43 (-0.43, -0.43)	-0.38 (-0.38, -0.37)
Portugal	-4.12 (-4.34, -3.90)	-4.15 (-4.37, -3.93)	-1.87 (-1.98, -1.75)	0.14 (0.14, 0.14)	0.21 (0.21, 0.21)	0.20 (0.20, 0.20)
Puerto Rico	-2.80 (-2.95, -2.65)	-3.05 (-3.21, -2.89)	-0.08 (-0.12, -0.05)	0.43 (0.43, 0.43)	0.56 (0.56, 0.57)	0.58 (0.58, 0.59)
Qatar	-2.50 (-2.74, -2.26)	-1.73 (-1.99, -1.47)	-0.37 (-0.47, -0.28)	-3.54 (-3.65, -3.42)	-2.67 (-2.74, -2.61)	0.21 (0.21, 0.21)
Republic of Korea	-5.39 (-5.61, -5.16)	-5.19 (-5.39, -4.98)	-2.34 (-2.48, -2.20)	1.09 (1.08, 1.10)	1.71 (1.68, 1.74)	0.27 (0.27, 0.27)
Republic of Moldova	-1.54 (-1.85, -1.22)	-1.72 (-2.00, -1.44)	-0.49 (-0.54, -0.44)	-15.70 (-18.07, -13.26)	-20.94 (-25.38, -16.23)	-0.42 (-0.42, -0.42)
Romania	-2.14 (-2.39, -1.89)	-2.06 (-2.27, -1.85)	-0.92 (-0.98, -0.86)	-1.72 (-1.75, -1.69)	-0.88 (-0.89, -0.88)	0.57 (0.57, 0.58)
Russian Federation	-1.33 (-1.95, -0.70)	-1.45 (-1.97, -0.92)	-0.45 (-0.55, -0.35)	-4.04 (-4.19, -3.89)	-3.50 (-3.61, -3.38)	-0.00 (-0.00, -0.00)
Rwanda	-2.91 (-3.23, -2.60)	-2.28 (-2.54, -2.03)	-1.02 (-1.11, -0.93)	0.24 (0.24, 0.24)	0.53 (0.53, 0.53)	0.13 (0.13, 0.13)
Saint Kitts and Nevis	-2.70 (-2.98, -2.43)	-2.25 (-2.49, -2.00)	-0.40 (-0.44, -0.37)	1.04 (1.03, 1.05)	-0.78 (-0.78, -0.77)	0.14 (0.14, 0.14)
Saint Lucia	-2.30 (-2.67, -1.94)	-2.57 (-2.98, -2.17)	-0.25 (-0.29, -0.22)	-0.04 (-0.04, -0.04)	0.11 (0.11, 0.11)	0.15 (0.15, 0.15)
Saint Vincent and the	-1.08 (-1.33, -0.83)	-0.94 (-1.24,	0.13 (0.11, 0.14)	-0.89 (-0.90, -0.89)	-0.90 (-0.91, -0.90)	0.18 (0.18, 0.18)

Grenadines		-0.63)				
Samoa	-0.41 (-0.43, -0.39)	-0.40 (-0.44, -0.36)	-0.09 (-0.13, -0.06)	-0.67 (-0.67, -0.66)	-0.60 (-0.61, -0.60)	0.12 (0.12, 0.12)
San Marino	-1.30 (-1.45, -1.15)	-1.31 (-1.44, -1.17)	-0.89 (-0.97, -0.81)	-0.43 (-0.43, -0.42)	-0.68 (-0.68, -0.67)	0.30 (0.30, 0.30)
Sao Tome and Principe	0.09 (-0.06, 0.23)	0.33 (0.24, 0.42)	0.19 (0.15, 0.23)	-0.99 (-0.99, -0.98)	-0.63 (-0.63, -0.62)	-0.02 (-0.02, -0.02)
Saudi Arabia	-0.65 (-0.85, -0.45)	-0.87 (-1.04, -0.69)	0.33 (0.22, 0.43)	-2.33 (-2.38, -2.28)	-2.56 (-2.62, -2.50)	-0.04 (-0.04, -0.04)
Senegal	-0.48 (-0.59, -0.36)	-0.34 (-0.43, -0.25)	-0.19 (-0.20, -0.17)	-2.16 (-2.20, -2.12)	-1.57 (-1.59, -1.54)	0.06 (0.06, 0.06)
Serbia	-1.68 (-1.94, -1.42)	-1.04 (-1.27, -0.81)	-0.40 (-0.46, -0.34)	-1.27 (-1.28, -1.25)	-1.85 (-1.88, -1.82)	0.48 (0.48, 0.48)
Seychelles	-1.61 (-1.70, -1.52)	-1.43 (-1.51, -1.35)	-0.22 (-0.24, -0.20)	-0.58 (-0.59, -0.58)	-0.29 (-0.29, -0.29)	0.30 (0.30, 0.30)
Sierra Leone	-0.08 (-0.21, 0.04)	-0.03 (-0.16, 0.09)	-0.00 (-0.02, 0.01)	-0.33 (-0.33, -0.33)	-0.57 (-0.57, -0.57)	-0.04 (-0.04, -0.04)
Singapore	-3.98 (-4.08, -3.87)	-3.89 (-4.01, -3.77)	-1.36 (-1.45, -1.28)	-2.28 (-2.32, -2.23)	-2.05 (-2.09, -2.01)	0.05 (0.05, 0.05)
Slovakia	-2.29 (-2.45, -2.14)	-1.88 (-2.03, -1.73)	-0.94 (-0.99, -0.88)	-0.64 (-0.64, -0.64)	-0.73 (-0.73, -0.72)	-0.85 (-0.86, -0.84)
Slovenia	-3.57 (-3.71, -3.42)	-3.31 (-3.44, -3.19)	-0.65 (-0.75, -0.55)	-0.40 (-0.40, -0.40)	-0.25 (-0.26, -0.25)	1.80 (1.78, 1.83)
Solomon Islands	0.03 (-0.02, 0.08)	0.16 (0.12, 0.21)	-0.08 (-0.11, -0.06)	0.48 (0.48, 0.48)	0.69 (0.69, 0.70)	-0.22 (-0.22, -0.22)
Somalia	-0.44 (-0.52, -0.37)	-0.21 (-0.29,	-0.24 (-0.26,	-0.56 (-0.57, -0.56)	-0.37 (-0.37, -0.37)	0.09 (0.09, 0.09)

		-0.12)	-0.21)			
South Africa	-0.61 (-1.09, -0.13)	-0.09 (-0.59, 0.40)	-0.30 (-0.39, -0.22)	-8.04 (-8.64, -7.44)	-9.29 (-10.09, -8.48)	0.26 (0.26, 0.27)
South Sudan	-0.97 (-1.10, -0.84)	-0.80 (-0.92, -0.69)	-0.09 (-0.12, -0.07)	-0.64 (-0.64, -0.64)	-0.43 (-0.44, -0.43)	0.17 (0.17, 0.17)
Spain	-3.03 (-3.15, -2.90)	-3.02 (-3.15, -2.89)	-1.38 (-1.46, -1.29)	-0.29 (-0.29, -0.29)	-0.20 (-0.20, -0.20)	0.51 (0.50, 0.51)
Sri Lanka	-1.62 (-1.78, -1.45)	-1.38 (-1.54, -1.21)	-0.43 (-0.49, -0.37)	-2.05 (-2.09, -2.02)	-2.47 (-2.53, -2.42)	-0.39 (-0.39, -0.39)
Sudan	-1.35 (-1.38, -1.32)	-1.10 (-1.15, -1.06)	-0.26 (-0.29, -0.23)	-0.60 (-0.60, -0.60)	0.06 (0.06, 0.06)	0.10 (0.10, 0.10)
Suriname	-1.34 (-1.63, -1.06)	-1.33 (-1.65, -1.00)	0.10 (0.05, 0.15)	-0.13 (-0.13, -0.13)	-0.56 (-0.56, -0.56)	0.28 (0.28, 0.28)
Sweden	-2.77 (-2.86, -2.68)	-2.64 (-2.72, -2.56)	-0.88 (-0.93, -0.82)	-0.30 (-0.30, -0.30)	-0.13 (-0.13, -0.13)	-1.12 (-1.13, -1.11)
Switzerland	-3.24 (-3.36, -3.12)	-2.92 (-3.02, -2.82)	-0.94 (-1.04, -0.84)	-0.61 (-0.61, -0.60)	-0.70 (-0.71, -0.70)	-1.56 (-1.59, -1.54)
Syrian Arab Republic	-1.57 (-1.80, -1.35)	-1.07 (-1.26, -0.88)	-0.22 (-0.29, -0.15)	-0.01 (-0.01, -0.01)	0.08 (0.08, 0.08)	-0.14 (-0.14, -0.14)
Taiwan (Province of China)	-2.79 (-3.01, -2.56)	-3.18 (-3.43, -2.92)	-0.76 (-0.80, -0.72)	-0.54 (-0.54, -0.54)	-0.74 (-0.74, -0.73)	0.34 (0.34, 0.34)
Tajikistan	1.03 (0.83, 1.23)	1.77 (1.50, 2.05)	0.49 (0.47, 0.52)	-4.69 (-4.89, -4.49)	-5.11 (-5.35, -4.87)	0.03 (0.03, 0.03)
Thailand	-2.37 (-2.55, -2.18)	-2.65 (-2.81, -2.48)	-0.80 (-0.86, -0.74)	-0.01 (-0.01, -0.01)	0.22 (0.22, 0.22)	0.11 (0.11, 0.11)
Timor-Leste	0.73 (0.60, 0.87)	0.95 (0.84, 1.05)	0.05 (0.04, 0.07)	0.81 (0.81, 0.82)	0.87 (0.86, 0.88)	0.01 (0.01, 0.01)
Togo	-0.29 (-0.37, -0.22)	-0.24 (-0.30,	-0.00 (-0.01,	-1.60 (-1.62, -1.58)	-1.19 (-1.21, -1.18)	-0.12 (-0.12,

		-0.18)	0.01)			-0.12)
Tokelau	-0.75 (-0.80, -0.69)	-0.64 (-0.69, -0.58)	-0.10 (-0.12, -0.07)	-1.06 (-1.07, -1.05)	-1.05 (-1.06, -1.04)	0.11 (0.11, 0.11)
Tonga	-0.26 (-0.37, -0.14)	-0.20 (-0.34, -0.06)	-0.07 (-0.12, -0.02)	-0.86 (-0.87, -0.86)	-0.81 (-0.82, -0.80)	0.27 (0.27, 0.27)
Trinidad and Tobago	-2.69 (-2.94, -2.45)	-2.73 (-2.98, -2.49)	-0.36 (-0.40, -0.33)	0.40 (0.40, 0.41)	0.53 (0.53, 0.53)	0.26 (0.26, 0.26)
Tunisia	-1.07 (-1.14, -0.99)	-0.92 (-1.01, -0.84)	-0.09 (-0.12, -0.06)	-1.26 (-1.28, -1.25)	-1.31 (-1.33, -1.30)	0.26 (0.26, 0.26)
Turkmenistan	-0.84 (-1.23, -0.45)	-0.90 (-1.26, -0.53)	-0.16 (-0.22, -0.10)	-0.13 (-0.13, -0.13)	0.29 (0.29, 0.29)	0.22 (0.22, 0.22)
Tuvalu	-0.59 (-0.63, -0.56)	-0.48 (-0.50, -0.46)	-0.04 (-0.06, -0.01)	-0.75 (-0.75, -0.74)	-0.65 (-0.65, -0.65)	0.09 (0.09, 0.10)
Uganda	-0.75 (-0.99, -0.51)	-0.57 (-0.78, -0.37)	-0.41 (-0.48, -0.34)	-0.83 (-0.84, -0.82)	-0.37 (-0.37, -0.37)	0.30 (0.30, 0.30)
Ukraine	-0.12 (-0.49, 0.25)	-0.35 (-0.68, -0.02)	-0.07 (-0.22, 0.07)	-1.74 (-1.77, -1.72)	-0.34 (-0.35, -0.34)	-0.10 (-0.10, -0.10)
United Arab Emirates	-1.91 (-2.28, -1.53)	-2.05 (-2.55, -1.55)	-0.03 (-0.05, -0.01)	0.79 (0.78, 0.79)	1.15 (1.14, 1.17)	0.11 (0.11, 0.11)
United Kingdom	-3.52 (-3.73, -3.30)	-3.44 (-3.66, -3.23)	-1.30 (-1.47, -1.13)	0.27 (0.27, 0.27)	0.28 (0.28, 0.28)	0.77 (0.76, 0.78)
United Republic of Tanzania	-0.49 (-0.57, -0.42)	-0.23 (-0.31, -0.15)	0.10 (0.08, 0.13)	-0.51 (-0.51, -0.50)	-0.39 (-0.39, -0.39)	0.30 (0.30, 0.30)
United States Virgin Islands	-0.26 (-0.44, -0.08)	-0.14 (-0.31, 0.03)	0.12 (0.11, 0.13)	-1.14 (-1.16, -1.13)	-1.10 (-1.11, -1.09)	0.09 (0.09, 0.09)
United States of America	-1.90 (-2.03, -1.77)	-2.12 (-2.28,	-1.23 (-1.30,	-0.06 (-0.06, -0.06)	0.13 (0.13, 0.13)	0.15 (0.15, 0.15)

		-1.97)	-1.16)			
Uruguay	-2.42 (-2.52, -2.31)	-2.32 (-2.43, -2.21)	-0.80 (-0.84, -0.77)	-0.70 (-0.71, -0.70)	-0.35 (-0.35, -0.35)	0.44 (0.44, 0.45)
Uzbekistan	1.70 (1.13, 2.27)	2.35 (1.71, 3.00)	1.24 (0.98, 1.49)	-3.93 (-4.07, -3.79)	-4.67 (-4.87, -4.47)	-0.74 (-0.74, -0.73)
Vanuatu	0.00 (-0.11, 0.12)	0.03 (-0.07, 0.12)	-0.11 (-0.12, -0.09)	-0.07 (-0.07, -0.07)	-0.03 (-0.03, -0.03)	-0.03 (-0.03, -0.03)
Venezuela (Bolivarian Republic of)	-1.46 (-1.62, -1.29)	-1.37 (-1.54, -1.19)	-0.69 (-0.77, -0.61)	1.86 (1.83, 1.89)	1.96 (1.92, 1.99)	-0.10 (-0.10, -0.10)
Viet Nam	-0.40 (-0.47, -0.32)	-0.26 (-0.35, -0.18)	0.10 (0.09, 0.11)	-1.31 (-1.33, -1.29)	-1.31 (-1.33, -1.30)	0.30 (0.30, 0.30)
Yemen	-1.01 (-1.10, -0.92)	-0.78 (-0.86, -0.71)	-0.19 (-0.22, -0.16)	0.85 (0.85, 0.86)	0.76 (0.76, 0.77)	0.06 (0.06, 0.06)
Zambia	-0.24 (-0.37, -0.12)	0.03 (-0.05, 0.11)	-0.27 (-0.31, -0.23)	-1.28 (-1.29, -1.26)	-0.91 (-0.92, -0.91)	0.13 (0.13, 0.13)
Zimbabwe	1.05 (0.81, 1.29)	0.89 (0.67, 1.12)	0.42 (0.34, 0.50)	-1.57 (-1.59, -1.55)	-1.35 (-1.37, -1.34)	-0.19 (-0.19, -0.19)
Zambia	-0.29 (-0.26, -0.31)	-0.53 (-0.49, -0.58)	-0.11 (-0.10, -0.12)	0.06 (0.07, 0.04)	-0.26 (-0.26, -0.27)	-0.45 (-0.44, -0.45)
Zimbabwe	0.86 (0.92, 0.80)	-0.22 (-0.18, -0.26)	0.18 (0.18, 0.17)	-0.08 (-0.07, -0.09)	0.73 (0.74, 0.72)	-0.18 (-0.17, -0.18)

STable 8. Buttom 10 countries of the projected age-standardized rates of CVD in 2030.

Rank	DALYs Country	DALYs Value(95%UI)	Deaths Country	Deaths Value(95%UI)	Incidence Country	Incidence Value(95%UI)
1	Limpopo	736.38 (-261.20, 1733.95)	Oman	-15.59 (-123.00, 91.83)	Peru	316.41 (307.18, 325.65)
2	Eastern Cape	933.38 (-1133.72, 3000.48)	Limpopo	8.32 (-38.43, 55.07)	Bolivia (Plurinational State of)	364.90 (356.44, 373.35)
3	Mpumalanga	985.63 (-1884.64, 3855.90)	Republic of Moldova	24.00 (-200.03, 248.03)	Ecuador	384.93 (372.49, 397.36)
4	Republic of Moldova	1052.33 (-3742.22, 5846.88)	Mpumalanga	37.64 (-87.91, 163.19)	Malta	406.85 (389.03, 424.67)
5	Kagoshima	1228.51 (1077.91, 1379.12)	Eastern Cape	45.11 (-45.21, 135.44)	Colombia	409.66 (401.46, 417.85)
6	Fukui	1300.22 (1122.40, 1478.04)	Kagoshima	57.18 (48.14, 66.23)	Amazonas	424.44 (420.15, 428.74)
7	Kanagawa	1321.11 (1123.39, 1518.83)	Hiroshima	59.54 (50.49, 68.59)	Switzerland	433.00 (414.43, 451.58)
8	Ōsaka	1322.66 (1103.23, 1542.09)	Ōsaka	60.21 (46.09, 74.33)	Republic of Korea	435.07 (411.61, 458.53)
9	Quintana Roo	1323.16 (865.69, 1780.63)	KwaZulu-Natal	60.27 (-29.19, 149.73)	Goiás	439.88 (430.38, 449.38)
10	Shiga	1324.69 (1168.61, 1480.76)	Hokkaidō	61.62 (50.59, 72.64)	Distrito Federal	440.32 (427.25, 453.40)

STable 9. Top 10 countries of the projected age-standardized rates of CVD in 2030.

Rank	DALYs (Disability-Adjusted Life Years) Country	DALYs (Disability-Adjusted Life Years) Value(95%UI)	Deaths Country	Deaths Value(95%UI)	Incidence Country	Incidence Value(95%UI)
1	Solomon Islands	21342.70 (19581.27, 23104.13)	Solomon Islands	885.13 (820.75, 949.50)	Ardebil	1429.77 (1374.28, 1485.25)
2	Kiribati	13583.75 (13030.69, 14136.81)	Mongolia	597.69 (477.19, 718.19)	North Khorasan	1425.34 (1367.83, 1482.85)
3	Vanuatu	13455.07 (12153.99, 14756.14)	Turkmenistan	574.34 (428.41, 720.27)	Khuzestan	1420.53 (1367.53, 1473.52)
4	Nauru	13047.98 (12409.61, 13686.34)	Ukraine	568.99 (392.03, 745.94)	Golestan	1408.22 (1351.64, 1464.80)
5	Micronesia (Federated States of)	12964.83 (12742.03, 13187.62)	Uzbekistan	566.95 (426.69, 707.21)	Markazi	1390.26 (1323.03, 1457.48)
6	Marshall Islands	12228.17 (10773.60, 13682.73)	Vanuatu	566.19 (522.84, 609.54)	Semnan	1368.68 (1322.21, 1415.15)
7	Mongolia	11662.18 (9838.85, 13485.50)	Micronesia (Federated States of)	560.59 (551.10, 570.09)	Kermansha h	1367.91 (1310.07, 1425.74)
8	Afghanistan	11439.64 (10613.11, 12266.17)	Yemen	557.51 (542.90, 572.12)	Uzbekistan	1367.26 (1273.29, 1461.22)
9	Yemen	11384.13 (10967.76, 11800.50)	Afghanistan	550.98 (521.82, 580.14)	Alborz	1363.11 (1316.54, 1409.68)
10	Turkmenistan	11295.68 (8044.48, 14546.89)	Nauru	548.82 (525.72, 571.91)	Kerman	1360.22 (1298.36, 1422.08)

STable 10. Buttom 10 countries of the projected EAPC of age-standardized rates of CVD in 2030.

Rank	DALYs Country	DALYs Value(95%CI)	Deaths Country	Deaths Value(95%CI)	Incidence Country	Incidence Value(95%CI)
1	Republic of Moldova	-15.70 (-18.07, -13.26)	Oman	-22.98 (-28.06, -17.55)	Germany	-2.47 (-2.52, -2.41)
2	Oman	-12.90 (-14.47, -11.30)	Republic of Moldova	-20.94 (-25.38, -16.23)	Malta	-2.30 (-2.35, -2.25)
3	South Africa	-8.04 (-8.64, -7.44)	South Africa	-9.29 (-10.09, -8.48)	Iceland	-2.13 (-2.17, -2.08)
4	Lithuania	-5.37 (-5.64, -5.11)	Bahrain	-7.26 (-7.74, -6.77)	Austria	-2.10 (-2.15, -2.06)
5	Jamaica	-4.82 (-5.03, -4.61)	Azerbaijan	-5.45 (-5.73, -5.18)	Switzerland	-1.56 (-1.59, -1.54)
6	Tajikistan	-4.69 (-4.89, -4.49)	Montenegro	-5.43 (-5.70, -5.16)	Norway	-1.51 (-1.53, -1.49)
7	Russian Federation	-4.04 (-4.19, -3.89)	Kyrgyzstan	-5.36 (-5.62, -5.09)	Georgia	-1.31 (-1.33, -1.29)
8	Uzbekistan	-3.93 (-4.07, -3.79)	Tajikistan	-5.11 (-5.35, -4.87)	Sweden	-1.12 (-1.13, -1.11)
9	Qatar	-3.54 (-3.65, -3.42)	Uzbekistan	-4.67 (-4.87, -4.47)	New Zealand	-1.04 (-1.05, -1.03)
10	Dominican Republic	-3.50 (-3.61, -3.39)	Lithuania	-4.25 (-4.41, -4.08)	Luxembourg	-0.91 (-0.92, -0.91)

STable 11. Top 10 countries of the projected EAPC of age-standardized rates of CVD in 2030.

Rank	DALYs Country	DALYs Value(95%CI)	Deaths Country	Deaths Value(95%CI)	Incidence Country	Incidence Value(95%CI)
1	Cabo Verde	4.08 (3.93, 4.23)	Cabo Verde	4.82 (4.61, 5.04)	Slovenia	1.80 (1.78, 1.83)
2	Venezuela (Bolivarian Republic of)	1.86 (1.83, 1.89)	Greece	2.54 (2.48, 2.60)	Chile	1.68 (1.66, 1.71)
3	Greece	1.65 (1.63, 1.68)	Venezuela (Bolivarian Republic of)	1.96 (1.92, 1.99)	Cyprus	1.14 (1.12, 1.15)
4	Belize	1.22 (1.20, 1.23)	Republic of Korea	1.71 (1.68, 1.74)	Japan	1.04 (1.03, 1.05)
5	Bahrain	1.19 (1.18, 1.20)	Belize	1.57 (1.55, 1.59)	Iran (Islamic Republic of)	0.92 (0.91, 0.93)
6	Israel	1.15 (1.14, 1.16)	Israel	1.19 (1.18, 1.20)	Italy	0.82 (0.81, 0.82)
7	Republic of Korea	1.09 (1.08, 1.10)	United Arab Emirates	1.15 (1.14, 1.17)	Estonia	0.79 (0.79, 0.80)
8	Saint Kitts and Nevis	1.04 (1.03, 1.05)	Iraq	1.09 (1.08, 1.10)	United Kingdom	0.77 (0.76, 0.78)
9	Jordan	0.86 (0.86, 0.87)	Jordan	1.07 (1.06, 1.08)	Libya	0.68 (0.67, 0.68)
10	Yemen	0.85 (0.85, 0.86)	Canada	0.97 (0.97, 0.98)	Netherlands	0.66 (0.65, 0.66)
